# Promoting Photocatalytic Direct C–H Difluoromethylation of Heterocycles using Synergistic Dual-Active-Centered Covalent Organic Frameworks

**DOI:** 10.1021/jacs.3c12880

**Published:** 2024-03-19

**Authors:** Sizhe Li, Wenxin Wei, Kai Chi, Calum T. J. Ferguson, Yan Zhao, Kai A. I. Zhang

**Affiliations:** † Department of Materials Science, Fudan University, 200433 Shanghai, P. R. China; ‡ Max Planck Institute for Polymer Research, 55128 Mainz, Germany; § School of Chemistry, University of Birmingham, University Road W, Birmingham B15 2TT, United Kingdom

## Abstract

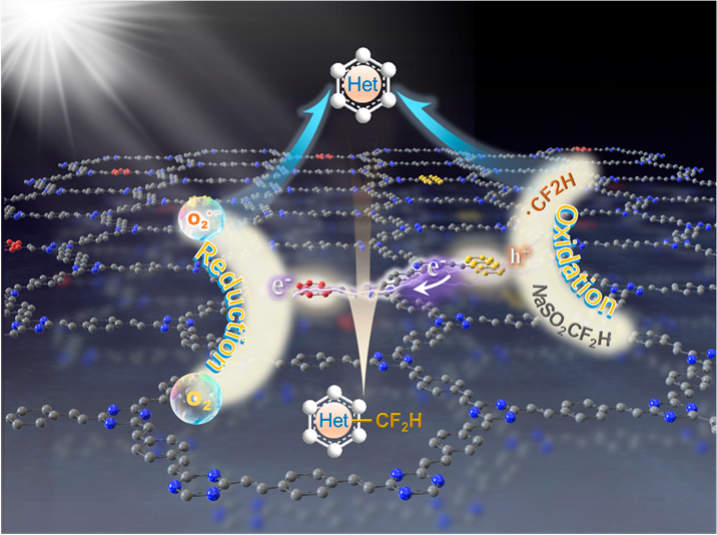

Difluoromethylation reactions are increasingly important for the creation of fluorine-containing heterocycles, which are core groups in a diverse range of biologically and pharmacologically active ingredients. Ideally, this typically challenging reaction could be performed photocatalytically under mild conditions. To achieve this separation of redox processes would be required for the efficient generation of difluoromethyl radicals and the reduction of oxygen. A covalent organic framework photocatalytic material was, therefore, designed with dual reactive centers. Here, anthracene was used as a reduction site and benzothiadiazole was used as an oxidation site, distributed in a tristyryl triazine framework. Efficient charge separation was ensured by the superior electron-donating and -accepting abilities of the dual centers, creating long-lived photogenerated electron–hole pairs. Photocatalytic difluoromethylation of 16 compounds with high yields and remarkable functional group tolerance was demonstrated; compounds included bioactive molecules such as xanthine and uracil. The structure–function relationship of the dual-active-center photocatalyst was investigated through electron spin resonance, femtosecond transient absorption spectroscopy, and density functional theory calculations.

## Introduction

Fluorine-substituted compounds have garnered significant attention in the fields of pharmaceuticals, agrochemicals, and material science due to their capacity to modulate lipophilicity, enhance biological activities, and increase metabolic stability.^1^ Specifically, heterocyclic structures incorporating difluoromethyl groups (CF_2_H) have various applications in pharmaceutical and agrichemical compounds.^2^ Direct photocatalytic C–H difluoromethylation has emerged as an efficient and widely adopted method for the synthesis of these organofluoride molecules.^3^ The oxidation of substrates, the generation of CF_2_H radicals, and the production of reactive oxygen species play pivotal roles in enhancing the efficiency of these reactions. Developing a robust photocatalytic system capable of simultaneously facilitating the generation of CF_2_H radicals and the production of reactive oxygen presents a formidable challenge. From a mechanistic standpoint, the oxygen reduction half-reaction necessitates the involvement of photogenerated electrons, while the CF_2_H radical generation half-reaction relies on photogenerated holes. Therefore, an effective photocatalytic system must not only readily generate photogenerated electron–hole pairs under light excitation but also exhibit excellent charge-separation capabilities. This allows both half-reactions to occur in tandem, while minimizing recombination rates. Currently, only a limited number of photocatalysts have been produced that can efficiently separate electron–hole pairs to perform challenging photocatalytic reaction.^4^

In nature, enzymes exhibit remarkable catalytic proficiency by harnessing multiple catalytic centers within a heterogeneous structure. These active centers work in cooperation to accelerate chemical reactions by several orders of magnitude^5^ (e.g., by 10^5^- to 10^20^-fold^6^). Inspired by natural biosynthetic processes, researchers have designed multicatalytic systems, where complex multicomponent catalytic pathways can be designed to facilitate complex organic reactions.^7^ Therefore, achieving high yields in the photocatalytic direct C–H difluoromethylation of heterocycles may be attainable through a dual-active-center strategy within heterogeneous catalytic materials.

Photocatalytic covalent organic frameworks (COFs) have garnered attention due to their well-defined pore structures and extended organic conjugation.^8^ COFs are often linked by covalent bonds, such as imine or borate bonds, which are often not stable enough for photocatalytic organic transformations due to their reversible nature.^9,10^ However, the newly developed vinylidene-linked COFs (V-COFs) exhibit greater chemical stability.^11^ In recent years, several COFs types and approaches to enhance their photocatalytic activity have emerged, including donor–acceptor COFs,^12^ photosensitive COFs,^13^ and COFs nanohybrids.^14^ COFs are modular systems and can be designed on the molecular level, allowing precise control over the composition of heterogeneous structure, This enables tuning of the electronic energy band structure as well as the number and composition of the catalytic active sites. Nevertheless, the construction and exploration of complete dual-active-center COF catalytic systems has been relatively limited.^10^

**Scheme 1:**
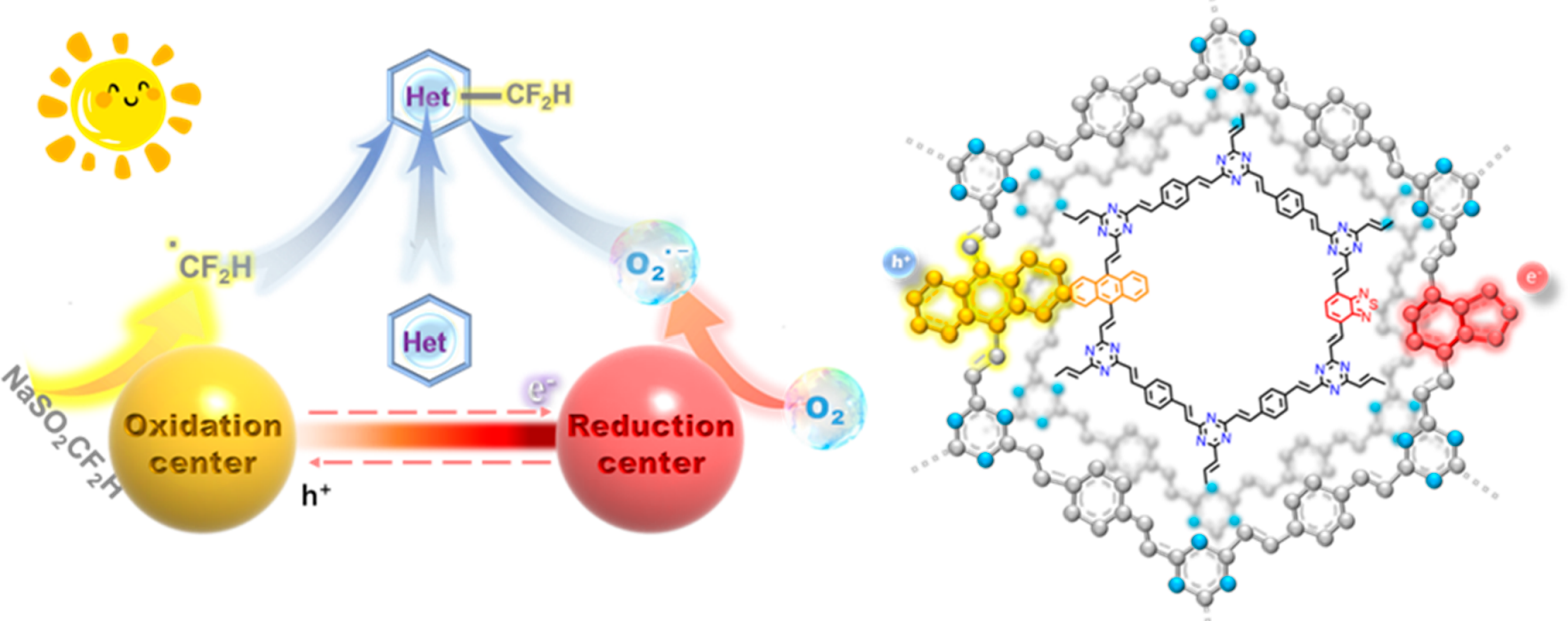
Direct Photocatalytic C–H Difluoromethylation of Heterocycles under Air and Visible Light with Dual-Active-Center COFs; Localization of Photogenerated Holes on Anthracene Units and Photogenerated Electrons on Benzothiadiazole Groups

Herein, we present a strategy for fabricating dual-active-center COFs by incorporating anthracene and benzothiadiazole moieties into a tristyryl triazine framework, thereby enhancing the photocatalytic performance for the direct C–H difluoromethylation of heterocycles (Scheme 1). The dual-active centers establish a charge transfer pathway, facilitating the efficient separation of photoexcited electrons and holes with visible light. Subsequently, the photogenerated electrons and holes are harnessed to activate molecular oxygen and generate CF_2_H radicals, reactants, respectively. These dual-active-center COFs demonstrate remarkable photocatalytic performance in the synthesis of various pharmaceutical products. The identification of radical reaction intermediates elucidated the reaction mechanism. Additionally, employing photoelectrochemical measurements and femtosecond transient absorption spectra (fs-TAS), we confirm that the incorporation of dual-active centers into COFs enhances effective charge separation and significantly increases the lifetime of the photogenerated species. Our approach presents a promising strategy for the design and development of highly efficient COFs-based photocatalysts for organic synthesis.

## Results and Discussion

Dual-active-center COF-based photocatalysts for the photocatalytic direct C–H difluoromethylation of heterocycles where produced. A control framework was initially produced composed of triazine and divinylbenzene, denoted as V-COF-1. Dual-active-center COFs were produced by copolymerizing 5 mol % anthracene-9,10-dicarbaldehyde (ARDA) and 5 mol % benzo[*c*][1,2,5]thiadiazole-4,7-dicarbaldehyde (BTDA) with 2,4,6-trimethyl-1,3,5-triazine (TMTA) and 1,4-diformylbenzene (DFB) via Knoevenagel condensation reaction (V-COF-AR-BT). Two reference COFs, namely V-COF-AR and V-COF-BT, were also produced by doping only 5 mol % of ARDA or BTDA, respectively, to incorporate only one single active site. Additional synthesis procedures and characterization data can be found in the Supporting Information.

**Figure 1:**
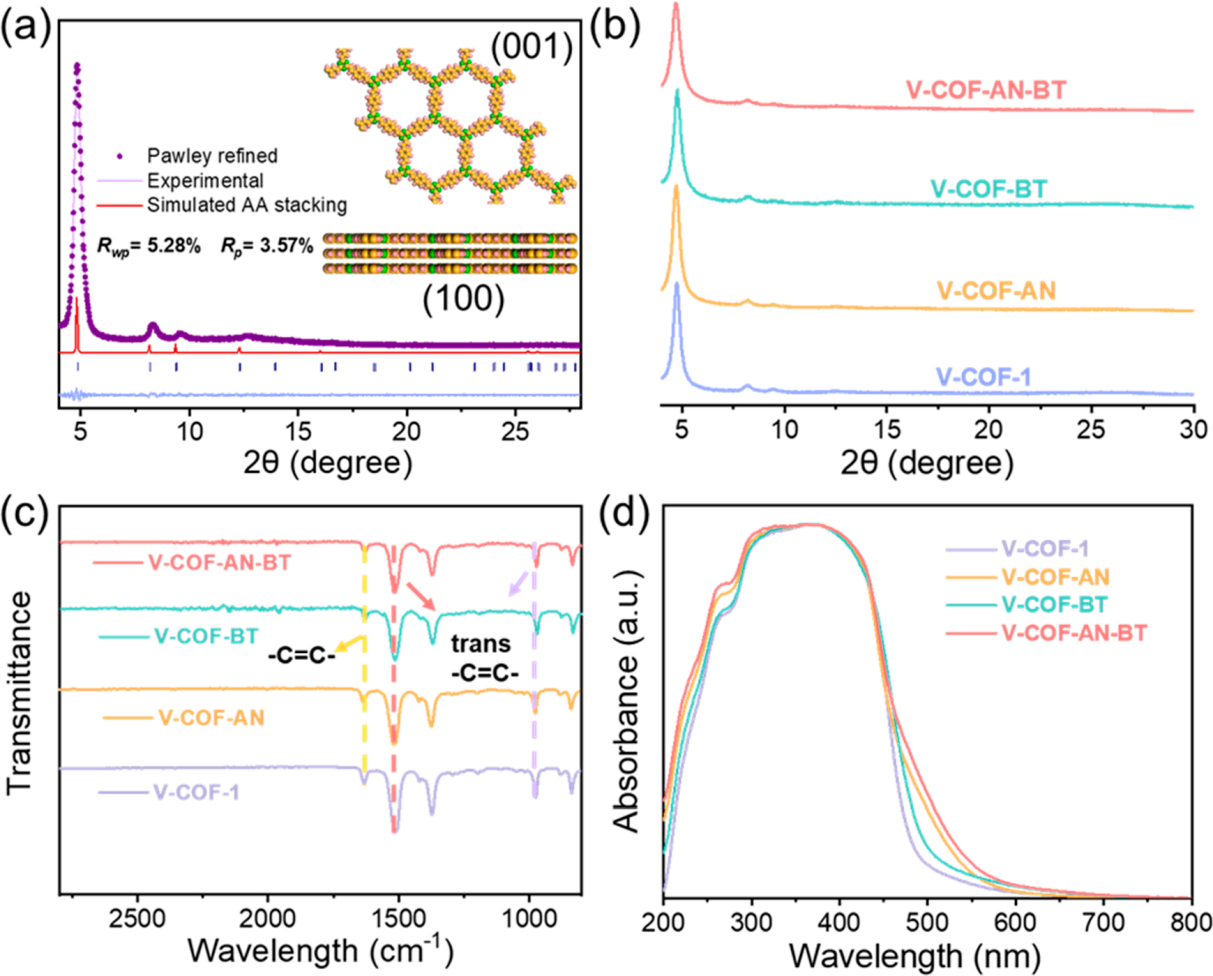
Structure and characterization of COFs. (a) PXRD patterns of V-COF-1: comparison between the experimental and Pawley refined profiles, the simulated patterns for eclipsed (AA) stacking mode, the Bragg positions, and the refinement differences; (b) PXRD patterns; (c) FT-IR spectra; (d) diffuse reflectance UV–vis spectra.

Powder X-ray diffraction (PXRD) analysis revealed reflections at 4.7°, 8.2°, 9.4°, 12.6°, and 26.6°, which were indexed as the (100), (110), (200), (210), and (001) lattices of V-COF-1, respectively (Figure 1a). These peaks were consistent with the reported and calculated patterns using the eclipsed stacking (AA) model in the P6 space group.^15^ The PXRD profiles of the synthesized COFs series (V-COF-AN, V-COF-BT, and V-COF-AN-BT) closely resembled that of undoped V-COF-1, indicating good crystallinity and retention of the same framework structure (Figure 1b). The Fourier transform infrared (FT-IR) spectrum of the COFs exhibited two distinctive peaks at 1636 and 971 cm^–1^, corresponding to the C=C stretching bands in the trans-configuration. Additionally, the characteristic peak of the triazine core was observed at approximately 1517 cm^–1^ (Figure 1c). This spectroscopic evidence reinforces the notion that the C=C connections between the triazine and benzene linkers form a porous hexagonal framework in the COFs. Furthermore, ^13^C NMR spectra of the COFs are presented in Figure S1, with assigned chemical shifts for the corresponding carbon atoms. Peaks at 106 and 153 ppm were attributed to the carbon atoms on vinylene bridges, while the peak at 170 ppm was ascribed to the carbon atoms in the 1,3,5-triazine ring in the COFs. Scanning electron microscope (SEM) and transmission electron microscope (TEM) images displayed a fibrillar morphology for the COFs (Figures S2 and S3). The high-resolution TEM (HRTEM) and the corresponding fast Fourier transformation (FFT) pattern demonstrated the long-range ordered structure of V-COF-AN-BT (Figure S4). The porosity and specific surface area of the COFs were evaluated by N_2_ gas sorption at 77 K (Figure S5). The Brunauer–Emmett–Teller (BET) surface areas of V-COF-1 and the COFs series were measured to be 1268, 1157, 1087, and 1012 m^2^ g^–1^, respectively. An increase in the quantity of other units led to a decrease in specific surface areas as they occupied the pore space of the COFs. Notably, all COFs exhibited a uniform and narrow pore width distribution centered around 1.2 nm (Figure S6). Ultraviolet–visible (UV–vis) light absorption spectra were measured to evaluate the optical properties of the COFs (Figure 1d). With the incorporation of AN and BT molecules, the absorption range of COF is subsequently extended to 600 nm, which is attributed to the larger conjugate structures of AN and BT compared to Ph, and this corresponds to narrowing of the optical bandgap and the redshift of the absorption peaks. Accordingly, the Kubelka–Munk transformed reflectance spectra (Figure S7) showed a band gap of 2.67 eV for V-COF-1, 2.61 eV for V-COF-AN, 2.57 eV for V-COF-BT, and 2.53 eV for V-COF-AN-BT, which is also consistent with the changing pattern of the structure of the COF series. Mott–Schottky (M–S) analysis (Figure S8) was performed to determine the lowest unoccupied molecular orbital (LUMO) levels and locate the lowest unoccupied molecular orbital ranging from −1.18 to −1.51 V versus normal hydrogen electrode (NHE). The corresponding highest occupied molecular orbital (HOMO) levels could be observed to range from +1.49 to +1.02 V versus NHE (Figure S9).

**Figure 2:**
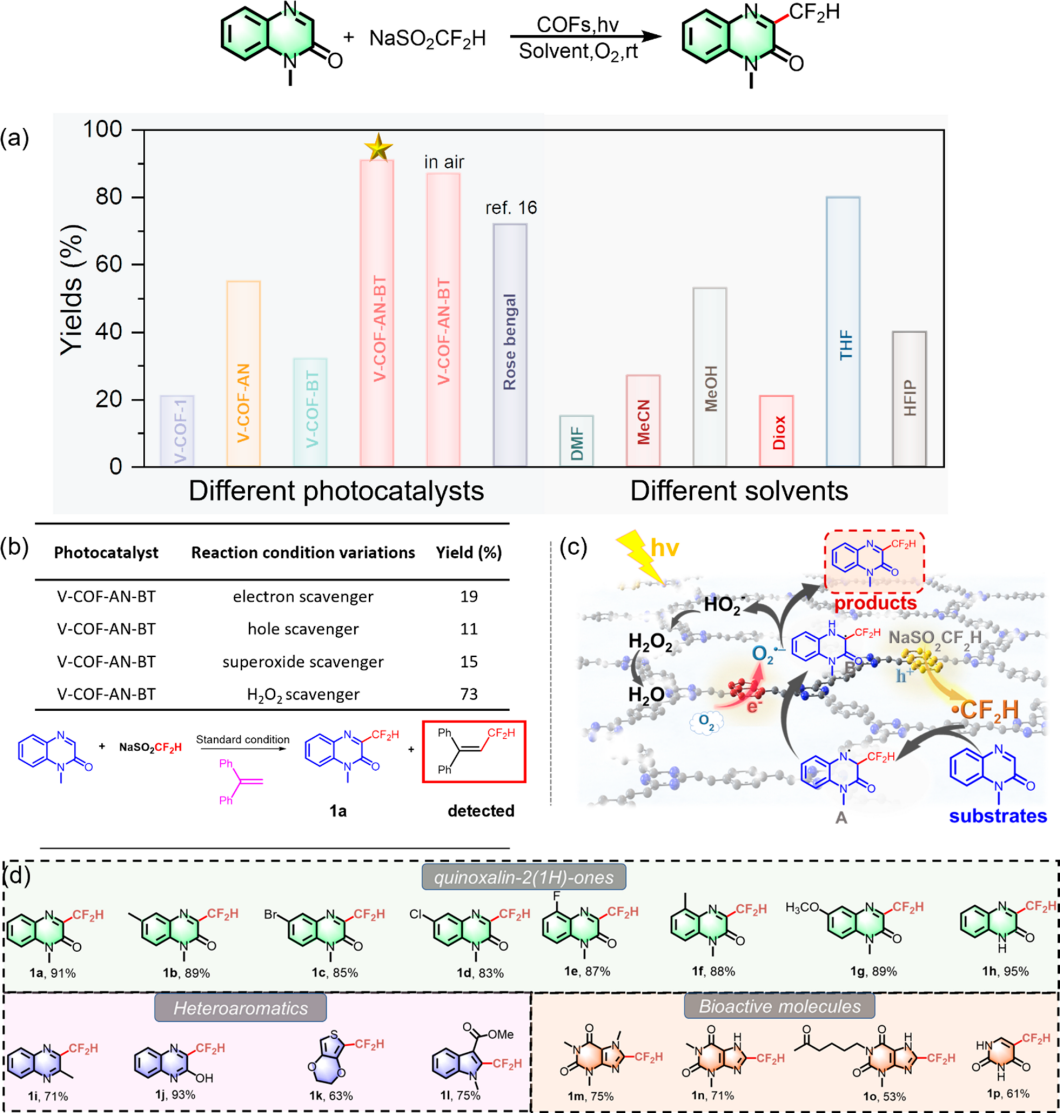
Photocatalytic direct C–H difluoromethylation of 1-methylquinoxolin-2-one using the COFs and substrates scope: (a) efficiency comparison of different photocatalysts under standard conditions and using V-COF-AN-BT over different solvents; (b) free radical and intermediate capture experiments; (c) probable mechanisms; (d) substrate scope for the photocatalytic direct C–H difluoromethylation of heterocycles.

Initial photocatalytic direct C–H difluoromethylation of heterocycles was undertaken with 1-methylquinoxolin as the model substrate, NaSO_2_CF_2_H as the fluorine source, molecular oxygen as the oxidant, and the COFs series as the photocatalyst in DMSO at room temperature under visible light irradiation. Our results, as shown in Figure 2, revealed that the combination of both anthracene and benzothiadiazole units into COFs (V-COF-AN-BT) significantly enhanced photocatalytic activity (91%) compared to the unsubstituted version (V-COF-1; 21%). This enhancement may be attributed to the synergistic function of oxidation and reduction centers providing reaction sites. Photogenerated electrons from anthracene centers may be efficiently transferred to nearby benzothiadiazole centers, thereby enhancing charge transfer and reducing recombination rate of the photogenerated species positively influencing the photocatalytic activity. In contrast, the introduction of anthracene or benzothiadiazole units separately into different COFs led to only slight improvements, with product yields of 55% and 32%, respectively. These observations indicate that although the introduction of photosensitive groups enhanced photocatalytic activity, the combination is needed for the specific promotion of difluoromethyl heterocycle formation. Notably, the photocatalytic activity of V-COF-AN surpasses that of V-COF-BT, possibly because photogenerated holes in V-COF-AN provide abundant reaction sites for the generation of difluoromethyl radicals. More materials for photocatalytic direct C–H difluoromethylation reactions were collected and listed in the Supporting Information (Table S1).

Control experiments conducted in various conditions, such as darkness, in the absence of oxygen, or without COFs as a photocatalyst, yielded only trace amounts of products, emphasizing the indispensable roles of light and the photocatalyst. Moreover, different reaction solvents were assessed, and DMSO emerged as the most effective medium through solvent screening (Figure 2a). To gain mechanistic insights and understand the roles of photogenerated electron/hole pairs, we introduced electron and hole scavengers into the reaction mixture. The results in Figure 2b, with only 19% and 11% yields in the presence of CuCl_2_ as an electron scavenger and KI as a hole scavenger, along with a reduced yield of 15% when 1,4-benzoquinone was used as a superoxide scavenger, suggest the active involvement of both species in the photocatalytic process. Notably, the absence of electron or hole scavengers hindered the reaction, indicating their essential roles. The reduced yield observed when a superoxide scavenger is present suggests that the electron-activated superoxide radical plays a crucial role in the reaction. This observation further supports the proposed mechanism, which involves charge transfer and separation within the COFs. These control experiments provide crucial insights into the mechanism and efficiency of the photocatalytic process, offering opportunities for optimizing reaction conditions and designing improved photocatalysts.

The proposed mechanism for the photocatalytic reaction is corroborated by control experiments and prior literature (Figure 2c).^16^ Upon visible light irradiation, the dual-active centers in the COFs effectively separate photogenerated electrons (e^–^) and holes (h^+^). Electrons localize on benzothiadiazole centers and react with adsorbed oxygen to form O_2_^•–^, while holes react with NaSO_2_CF_2_H to produce the CF_2_H radical. To confirm the presence of the radical intermediate, 1,1-diphenylethylene, a radical scavenger, was introduced into the mixture containing 1-methyl quinoxolin and NaSO_2_CF_2_H. The radical intermediate was detected by ESIHRMS (Figure 2b and Figure S10). Subsequently, the CF_2_H radical adds to the substrate to form intermediate A, which undergoes a 1,2-H shift, generating carbon radical intermediate B. The activated oxygen species O_2_^•–^ oxidizes intermediate B to produce the desired product, and the O_2_^•–^ likely forms first the HO_2_^–^ species, which then gains a hydrogen atom to form hydrogen peroxide (H_2_O_2_). *In situ*^1^H NMR experiments confirmed the generation of H_2_O_2_ during the reaction. However, the H_2_O_2_ peak disappeared in the final product spectra, while the H_2_O peak area increased. This suggests that H_2_O_2_, generated during the O_2_ conversion, participates in the catalytic cycle and is consumed to produce the final product H_2_O (Figure S11). Catalase was employed as a scavenger to consume H_2_O_2_, resulting in a yield of 73% (Figure 2b), which, while lower than the standard model reaction (91%), supports the overall reaction mechanism based on experimental evidence.

To further demonstrate the broad applicability of COFs, we investigated the substrate scope of heterocycles using V-COF-AN-BT as the photocatalyst. As shown in Figure 2d, a variety of substituted quinoxalin-2(1*H*)-ones with electron-donating or -withdrawing substituents performed well under optimal reaction conditions. Heteroaromatic substrates, including quinoxalines, indoles, and thiophenes, also exhibited successful reactions. Notably, nitrogen-containing bioactive molecules such as xanthine derivatives and uracil participated in the reaction with moderate to good yields, highlighting the potential for this transformation in natural product synthesis. In the gram-scale reaction, a product yield of 71% (0.93 g) was achieved with the addition of 60 mg of the photocatalyst, demonstrating the potential application of COFs. Further to difluoromethylation we have also shown that the photocatalytic COF can efficiently undertake trifluoromethylation reactions using CF_3_SO_2_Na as the fluorine source (Figure S27). Additionally, we assessed the stability and reusability of V-COF-AN-BT through repeated experiments, revealing no significant change in the catalytic activity after at least five cycles (Figure S12). Furthermore, FTIR (Figure S13) and SEM (Figure S14) images of V-COF-AN-BT showed minimal changes before and after repeated reactions.

**Figure 3:**
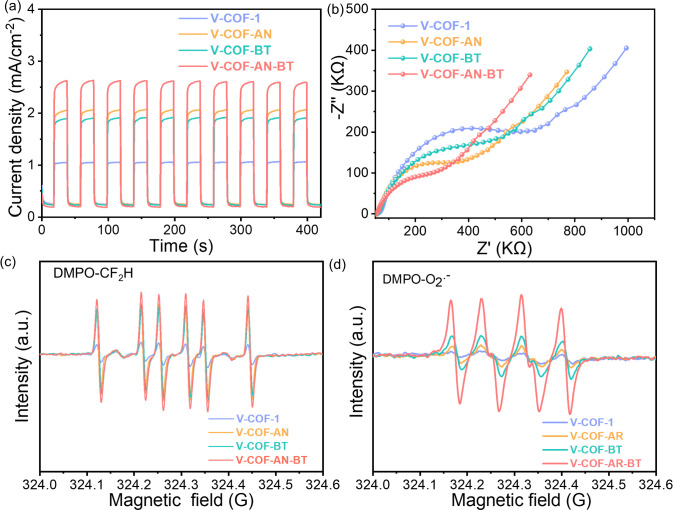
(a) Photocurrent; (b) impedance Nyquist plot; ESR spectra of (c) CF_2_H radical and (d) superoxide radical of the COFs.

The underlying mechanism of the COF series was further characterized by using several methods. Notably, the highest transient photocurrent response was observed for V-COF-AN-BT, indicative of enhanced charge separation compared to other samples (Figure 3a). Additionally, electrochemical impedance spectroscopy measurements unveiled a relatively smaller interfacial resistance for V-COF-AN-BT, suggesting faster interface charge transport (Figure 3b). Interestingly, for V-COF-1 a larger charge transfer resistance was detected, which decreased with substitution of the COF, with the lowest charge transfer resistance corresponding to the system with both Anthracene and benzothiadiazole within it. Time-resolved photoluminescence (TRPL) spectra demonstrated that V-COF-AN-BT possessed a remarkably shorter average lifetime of photogenerated charge carriers (4.90 ns) in comparison to that of other COFs (Figure S15). Decreased fluorescence lifetime has previously been shown to indicate an improved delocalization of photogenerated holes and electrons and more nonradiative recombination of the excitons.^17^ In addition, the lowest photoluminescence emission response was for V-COF-AN-BT which had a low radiative excursion rate as shown by its low degree of charge complexation and high charge transfer capability (Figure S16).^18^

To delve into the key radical intermediates and active species generated during the photocatalytic process, 2,2,6,6-tetramethyl-4-oxo-1-piperidinyloxy (TEMPO) were introduced as spin traps. The signal decreased over time due to its oxidation to TEMPO^+^ via h^+^, as depicted in Figure S17, this confirmed that the primary active species during the photocatalytic process were h^+^. Furthermore, the key radical intermediate, difluoromethyl, was observed in an EPR spin-trapping experiment with DMPO. It was observed that the ability of V-COF-AN-BT to generate difluoromethyl radicals was more pronounced than that of other COFs (Figure 3c). O_2_^•–^ was also monitored by EPR, and the intensity of V-COF-AN-BT-3 showed the highest signal compared with other COFs (Figure 3d).

**Figure 4:**
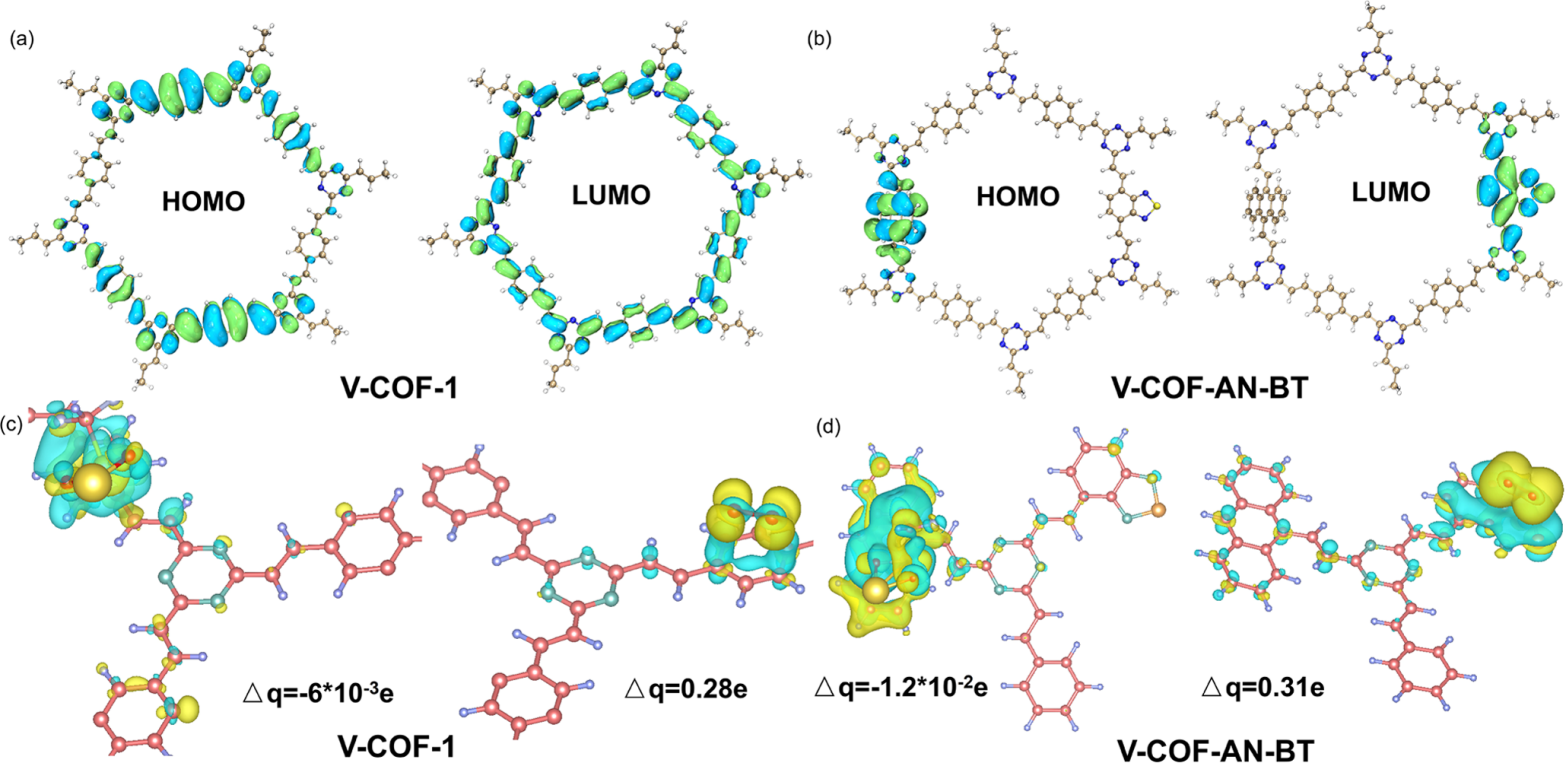
Calculated HOMO and LUMO for (a) the model molecule V-COF-1 and (b) V-COF-AN-BT; charge difference distribution after adsorption of NaSO_2_CF_2_H and oxygen to (c) V-COF-1 and (d) V-COF-AN-BT; Δq = Bader charge.

The charge dynamics in COFs is intimately linked to their electronic structure. In order to gain insight into the role of AR and BT units in promoting exciton dissociation, density functional theory (DFT) calculations using COFs fragments as model molecules were conducted. The electronic structure analysis revealed that in the absence of AR and BT units (Figure 4a), the highest occupied molecular orbital (HOMO) was primarily localized on the benzene rings, while the lowest unoccupied molecular orbital (LUMO) exhibited even delocalization across the conjugated skeleton, overlapping with the HOMO. However, upon the introduction of AR and BT units (Figure 4b), the HOMO became centralized around the AR unit, while the LUMO concentrated around the BT group due to its strong electron-withdrawing capability. Consequently, photoexcitation induced a HOMO–LUMO transition, leading to an electron concentration on the BT unit as the reduction center and hole concentration on the AR unit as the oxidation center. These dual-active centers facilitated efficient intramolecular charge separation within the COF backbone.^19^ Furthermore, based on the method developed by Richard Bader, we visualize the molecular Bader charge distribution through VASP calculations (Figure 4c, d),^20^ of NaSO_2_CF_2_H and AR centers within V-COF-AN-BT, revealing a value of −1.2 × 10^–2^ on the COFs surface, higher than the corresponding sites in V-COF-1 (−6 × 10^–3^). This indicates that the AR centers play a crucial role in facilitating the oxidation of NaSO_2_CF_2_H to form the CF_2_H radical. Similarly, the Bader charges of oxygen molecules were calculated to be 0.31e on the BT centers of V-COF-AN-BT, which was higher than the corresponding sites of V-COF-1 (0.28e). These results underscore the propensity of BT centers in V-COF-AN-BT to promote electron transfer to O_2_ for the production of O_2_^•–^.^21^ Overall, these findings strongly suggest that the presence of dual-active centers in COFs effectively promotes charge separation and transport, facilitating substrate oxidation and molecular oxygen activation, thereby significantly improving the photocatalytic performance

To further corroborate the existence of dual-active centers, we conducted an investigation into the direction of electron transfer during the photocatalytic process. In this regard, we utilized *in situ* X-ray photoelectron spectroscopy (XPS) analysis on V-COF-AN-BT and scrutinized the alterations in the binding energies of various elements in the presence and absence of light (Figure S18). Our findings unveiled a negative shift in the N and S spectra in the “after irradiation” sample, indicating that benzothiadiazole units captured electrons during light irradiation, consistent with our EPR results. Conversely, the binding energy of C exhibited a positive shift when compared to V-COF-AN-BT before irradiation, indicating a decrease in the electron density of anthracene units during light irradiation. These observations substantiate the occurrence of charge transfer from anthracene units to benzothiadiazole units throughout the photocatalytic reaction.^23^

**Figure 5:**
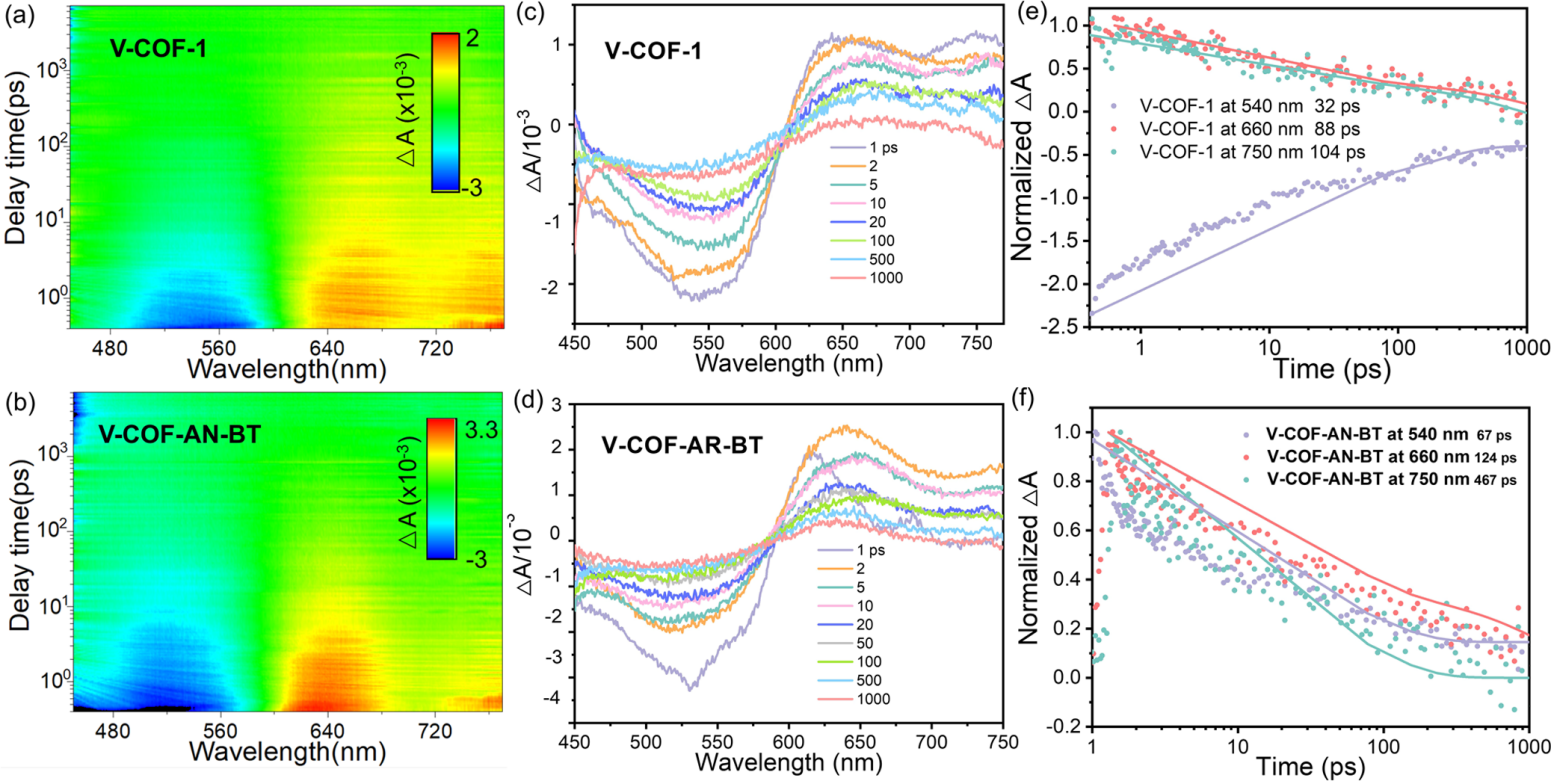
2D transient absorption surface plots of (a) V-COF-1 and (b) V-COF-AN-BT. Transient absorption signals of (c) V-COF-1 and (d) V-COF-AN-BT. The decay signals of (e) V-COF-1 and (f) V-COF-AN-BT.

Finally, the inherent charge-transfer mechanism in COFs was elucidated by using femtosecond transient absorption spectroscopy (fs-TAS) following 400 nm excitation (Figure 5a–f). The fs-TAS spectra of both V-COF-1 and V-COF-AN-BT displayed negative features at 520 and 500 nm, signifying ground-state bleaching. In our two-dimensional (2D) fs-TAS analysis, we observed absorption spectra over time and wavelength, unveiling ground-state bleach (GSB) and stimulated emission (SE) signals in V-COF-1 (Figure 5a) and V-COF-AN-BT (Figure 5b). Notably, V-COF-AN-BT exhibited a more pronounced GSB in comparison to V-COF-1, indicative of a higher population of photogenerated charge carriers in V-COF-AN-BT. The positive signal detected in both materials in the 600–750 nm range, with a peak at 640 nm, could be attributed to photoinduced electron transfer (PET) (Figure 5c, d). This phenomenon led to a new peak at 750 nm, signifying changes in the electron–hole interaction. The observed blueshift phenomenon further corroborated the transfer of photogenerated electrons from the donor unit to the acceptor unit within the COFs. Particularly noteworthy was the more pronounced blueshift observed in V-COF-AN-BT, coinciding with a more pronounced peak at 640 nm. This was attributed to the presence of dual-active centers within V-COF-AN-BT, showcasing precise structural designs that enhanced electron–hole separation.^22^ Notably, charge recombination times at 540, 660, and 750 nm for V-COF-AN-BT (67, 124, and 467 ps) were significantly longer than those of V-COF-1 (32, 88, and 104 ps), underscoring the extension of the excited state lifetime in V-COF-AN-BT (Figure 5e, f).

## Conclusion

In summary, we developed an efficient photocatalytic system for the synthesis of heterocyclic compounds featuring difluoromethyl groups. The rational synthesis of V-COF-AN-BT has allowed us to harness the electron-donating and -accepting capacities intrinsic to the dual centers within this framework. Photogenerated holes, required to produce difluoromethyl radicals, are generated as well as photogenerated electrons, which are essential for the reduction of oxygen. We used these materials for the photocatalytic difluoromethylation of 16 compounds, achieving yields of up to 91% and demonstrating high-functional group tolerance. This encompassed the synthesis of bioactive molecules, such as xanthine and uracil. Furthermore, we have investigated the structure–function relationship through EPR, fs-TAS, and DFT calculations. *In situ* XPS, EPR, and DFT calculations showed that reduction centers produced photogenerated electrons, activating molecular oxygen, while oxidation centers facilitated the oxidation of substrates by transporting photogenerated holes. This research represents a modular design strategy in photocatalytic COFs, where dual reactive centers can be used to create potent photocatalytic systems with a wide-ranging applicability.

## Supporting Information Available

Details of the synthetic route of the COFs, characterization methods, photobiocatalysis, etc. (PDF)
