# Supplementary material for: Promoting Photocatalytic Direct C–H Difluoromethylation of Heterocycles using Synergistic Dual-Active-Centered Covalent Organic Frameworks
Source: J Am Chem Soc. 2024 Mar 19;146(18):12386–94. doi: 10.1021/jacs.3c12880 (PMC11082899; doi:10.1021/jacs.3c12880)
Supplement: Supplementary file 1 — ja3c12880_si_001.pdf [file ja3c12880_si_001.pdf]

Supporting Information

Promoting Photocatalytic Direct C–H Difluoromethylation of Heterocycles using Synergistic Dual Active Centered Covalent Organic Frameworks

Sizhe Li,[a] Wenxin Wei,*[a] Kai Chi, [a] Calum T. J. Ferguson, *[b,c] Yan Zhao,*[a] and Kai A. I. Zhang.[a,b]

[a] Department of Materials Science, Fudan University, 200433 Shanghai, P. R. China

[b] Max Planck Institute for Polymer Research, 55128 Mainz, Germany

[c] School of Chemistry, University of Birmingham, University Rd W, Birmingham B15 2TT, U.K.

Table of Contents

Materials 3

Characterization...3

Preparation of COFs 5

General procedure of the photocatalytic reactions using COFs. 7

An overview of different products...8

Characterization...12

NMR spectra of products...26

Materials

2,4,6-Trimethyl-1,3,5-triazine (TMTA) and 1,3,5-tris(4-formylphenyl)benzene (TFPB) were prepared using reported methods.[1] 1,4-Diformylbenzene (DFB), KOH, sodium methoxide and sodium ethoxide were purchased from Macklin. All the solvents were purchased from Adamas-beta Reagent and used as received without further purification. Column flash chromatography was conducted with silica gel (200–300 mesh).

Characterization

Fourier transform infrared (FT-IR) spectra were collected on a VARIAN 1000 FT-IR spectrometer in the region of 400–4000 cm^{-1} by potassium bromide pressed-disk technique. The morphologies and microstructures were probed utilizing Scanning electron microscope (SEM, Nova NanoSEM 230) and transmission electron microscope (TEM, FEI TECNAI G2 F20). The cumulative apparent surface areas for N_2 were calculated on a Micro ASAP 2020 using a Brunauer–Emmett–Teller (BET) model range from 0.01 to 0.1 bar for all samples. The microporous volumes were calculated using the t-plot method, while the total porous volumes were obtained from the N_2 isotherm at P/P_0=0.99. Pore size distributions were derived from the N_2 adsorption isotherms using DFT methods. UV/Vis/NIR absorption spectra of the polymers were recorded on a UV-2600 spectrometer (Shimadzu, Japan) as powders in the solid state. Electrochemical measurements of these materials were carried out on a Metrohm Autolab PGSTAT204 at room temperature in a three-electrode cell, with the glassy carbon auxiliary electrode as working electrode, the platinum wire as counter electrode. And the Hg/Hg_2Cl_2 electrode was used as reference electrode. The polymer samples were coated onto the glassy carbon electrode. The solid-state ^{13}C NMR spectra were performed on a Bruker AVANCE III 400 MHz NMR spectrometer. ^{1}H and ^{13}C NMR spectra were obtained in deuterated solvents on Bruker AM-400 MHz using tetramethyl silane (TMS) as an internal standard. Electrostatic potential map was carried out by Standard ab initio molecular orbital theory and density functional theory calculations with the Gaussian 09 software package and Gauss View visualization program. The electron spin resonance (ESR) signals of the radicals that were spin-trapped by DMPO or TEMPO were recorded on the JES FA200 spectrometer (JEOL, Japan). Specimens for the ESR measurement were prepared by mixing the photocatalyst with 40mM DMPO or TEMPO solution in a beaker and illuminating it with visible light ($\lambda > 400$ nm). The ultrafast TA spectra of the samples were obtained using a laser system with amplification at a 1 KHz repetition rate and a Helios spectrometer with an ultrafast system. The laser pump pulse signal in the range of 425-900 nm was focused onto the sapphire disk to generate an ultrafast continuous probe beam. The probe light, which contained the sample and reference beam, was collected by the lens of the visible-light responsive fiber silicon or infrared-light responsive InGaAs diode array. The TA decay kinetic trace was measured by recording the average ΔA over the corresponding detection wavelength range for each delay. Thin films were prepared for all photocatalysts. To be specific, 30.0 mg of catalyst were dispersed in 1 mL of ethanol solution by sonication. The above suspension was then sprayed on 15×15×3 mm quartz slides and dried in air for 12 hours. *In situ* ^{1}H NMR: To a 10 mL Schlenk tube equipped with a magnetic stir bar, added quinoxalin-2(1*H*)-ones (0.2 mmol), $NaSO_2CF_2H$ (0.4 mmol) and COFs (8 mg) in DMSO-d_6 (2.0 mL). Then the mixture was stirred and irradiated by the

3W blue LEDs at room temperature for 3, 6 and 24 h. Then, the reaction solution is filtered for ^{1}H NMR characterization. The GC-MS spectra were measured by GCMS QP2020NX (Shimadzu, Japan), with GC process condition (60 to 180 ℃, 50 ℃/min; 180 to 250 ℃, 20 ℃/min, retention time 3 min; column Rtx-5MS) and MS process condition (temperature of ion source: 250 ℃, temperature of interface: 280 ℃, scan rate: 1250).

Preparation of COFs[2]

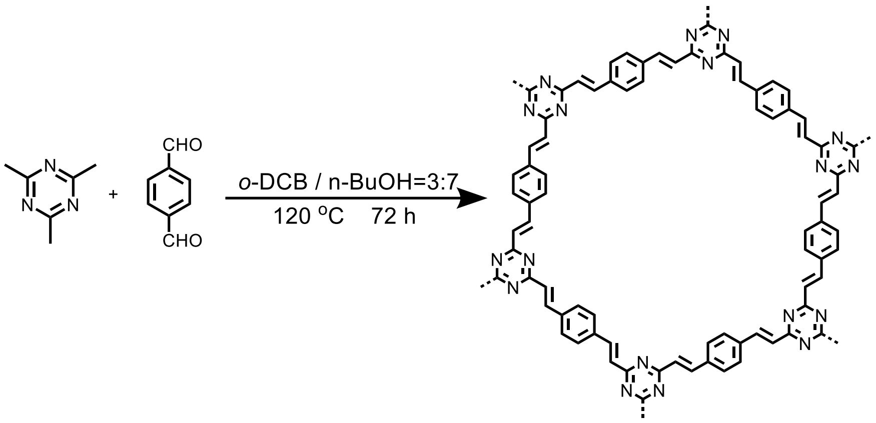

V-COF-1

Synthesis of V-COF-1. 2,4,6-Trimethyl-1,3,5-triazine (TMTA) (61.58 mg, 0.50 mmol), 1,4-diformylbenzene (DFB) (100.60 mg, 0.75 mmol) and KOH (84.15mg, 1.5mmol) were dissolved in a mixed solvent containing 7 mL n-Butanol and 3 mL 1,2-dichlorobenzene; Subsequently, the resulting pale-yellow solution was heated at 120 ℃ for a 3-day reaction. After cooling to room temperature, the precipitate was collected and washed with methanol, tetrahydrofuran, acetone and dichloromethane in sequence for three times (10 mL for each), and then dried under vacuum at 120 °C for 12 h. Finally, pure COF sample was afforded as yellow powder (yield: 79/%).

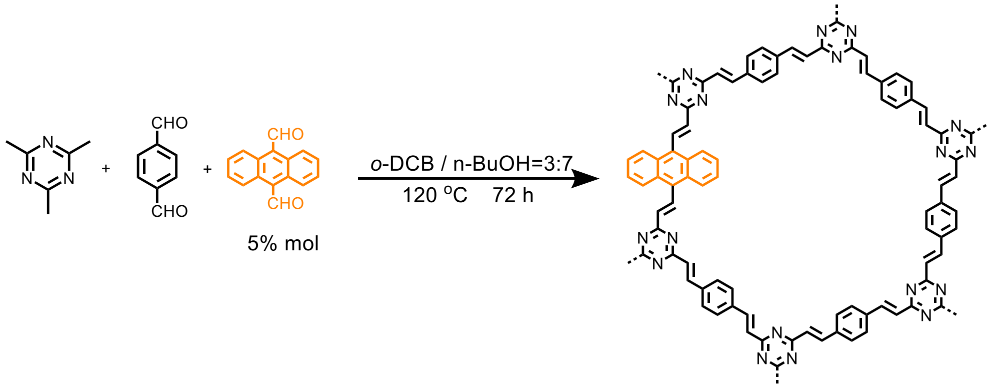

V-COF-AN

Synthesis of V-COF-AN. 2,4,6-Trimethyl-1,3,5-triazine (TMTA) (61.58 mg, 0.50 mmol), 1,4-diformylbenzene (DFB) (100.60 mg, 0.75 mmol), Anthracene-9,10-dicarbaldehyde (AN) (8.78 mg, 0.0375 mmol) and KOH (88.36 mg, 1.575 mmol) were dissolved in a mixed solvent containing 7 mL n-Butanol and 3 mL 1,2-dichlorobenzene; Subsequently, the resulting pale-yellow solution was heated at 120 ℃ for a 3-day reaction. After cooling to room temperature, the precipitate was collected and washed with methanol, tetrahydrofuran, acetone and dichloromethane in sequence for three times (10 mL for each), and then dried under vacuum at 120 °C for 12 h. Finally, pure COF sample was afforded as yellow powder (yield: 75/%, relative to the used amount of monomer).

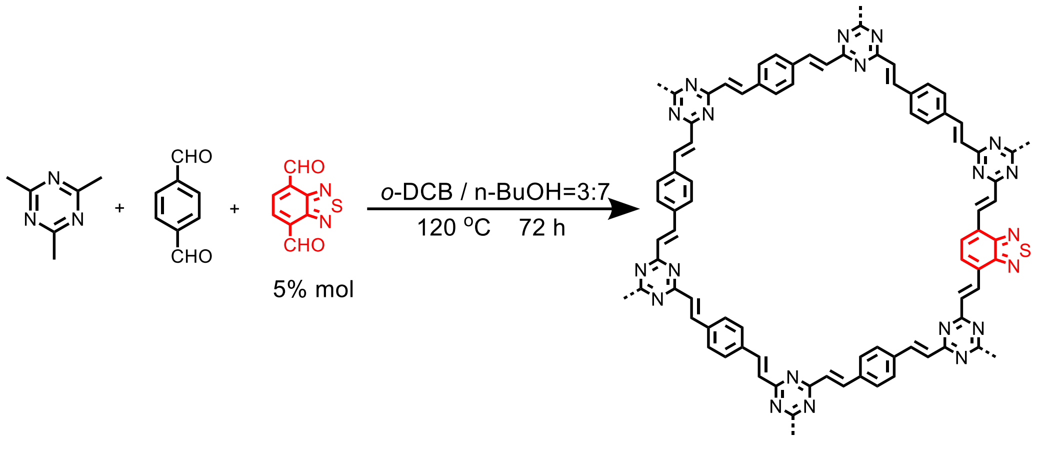

V-COF-BT

Synthesis of V-COF-BT. 2,4,6-Trimethyl-1,3,5-triazine (TMTA) (61.58 mg, 0.50 mmol), 1,4-diformylbenzene (DFB) (100.60 mg, 0.75 mmol), Benzo[c][1,2,5]thiadiazole-4,7-dicarbaldehyde (BT) (7.21 mg, 0.0375 mmol) and KOH (88.36 mg, 1.575 mmol) were dissolved in a mixed solvent containing 7 mL n-Butanol and 3 mL 1,2-dichlorobenzene; Subsequently, the resulting pale-yellow solution was heated at 120 °C for a 3-day reaction. After cooling to room temperature, the precipitate was collected and washed with methanol, tetrahydrofuran, acetone and dichloromethane in sequence for three times (10 mL for each), and then dried under vacuum at 120 °C for 12 h. Finally, pure COF sample was afforded as yellow powder (yield: 73/%, relative to the used amount of monomer).

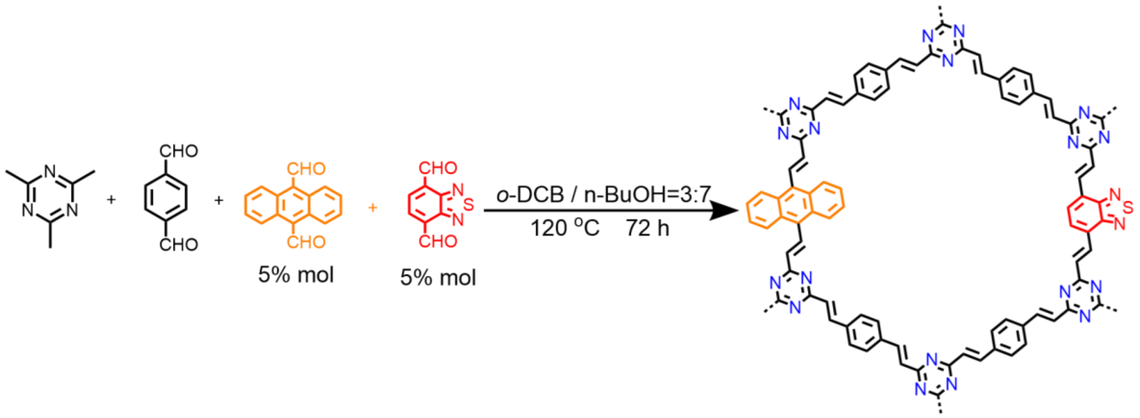

V-COF-AN-BT

Synthesis of V-COF-AN-BT. 2,4,6-Trimethyl-1,3,5-triazine (TMTA) (61.58 mg, 0.50 mmol), 1,4-diformylbenzene (DFB) (100.60 mg, 0.75 mmol), Anthracene-9,10-dicarbaldehyde (AN) (8.78 mg, 0.0375 mmol), Benzo[c][1,2,5]thiadiazole-4,7-dicarbaldehyde (BT) (7.21 mg, 0.0375 mmol) and KOH (92.57 mg, 1.65 mmol) were dissolved in a mixed solvent containing 7 mL n-Butanol and 3 mL 1,2-dichlorobenzene; Subsequently, the resulting pale-yellow solution was heated at 120 °C for a 3-day reaction. After cooling to room temperature, the precipitate was collected and washed with methanol, tetrahydrofuran, acetone and dichloromethane in sequence for three times (10 mL for each), and then dried under vacuum at 120 °C for 12 h. Finally, pure COF sample was afforded as yellow powder (yield: 75/%, relative to the used amount of monomer).

General procedure of the photocatalytic reactions using COFs.[3]

To a 10 mL Schlenk tube equipped with a magnetic stir bar, added quinoxalin-2(1*H*)-ones (0.2 mmol), $NaSO_2CF_2H$ (0.4 mmol) and COFs (8 mg) in DMSO (2.0 mL). Then the mixture was stirred and irradiated by the 3W blue LEDs at room temperature for 24 h. The residue was added water (10 mL) and extracted with ethyl acetate (5 mL x 3). The combined organic phase was dried over Na_2SO_4. The resulting crude residue was purified via column chromatography on silica gel to afford the desired products.

Photocatalytic recycling experiments.

The general procedure was followed for setting up the reactions. After the completion of a reaction cycle after 24h, the reaction mixture was centrifuged at 10 000 rpm for 1 min. Then, the supernatant was removed and fresh CH_2Cl_2 (2mL) was added for washing. The centrifugation was repeated and residual CH_2Cl_2 was removed. The nanoparticles were then dried for the next reaction.

Gram-scale reaction:

To a 100 mL round flask equipped with a magnetic stir bar, added quinoxalin-2(1*H*)-ones (1.0g, 6.25 mol), $NaSO_2CF_2H$ (1.72g, 12.30 mmol) and V-COFs-AN-BT (60 mg) in DMSO (60 mL). Then the mixture was stirred and irradiated by the KESSIL LEDs at room temperature for 24 h. The residue was added water and extracted with ethyl acetate. The combined organic phase was dried over Na_2SO_4. The resulting crude residue was purified via column chromatography on silica gel to afford the desired products (0.93g, 71%).

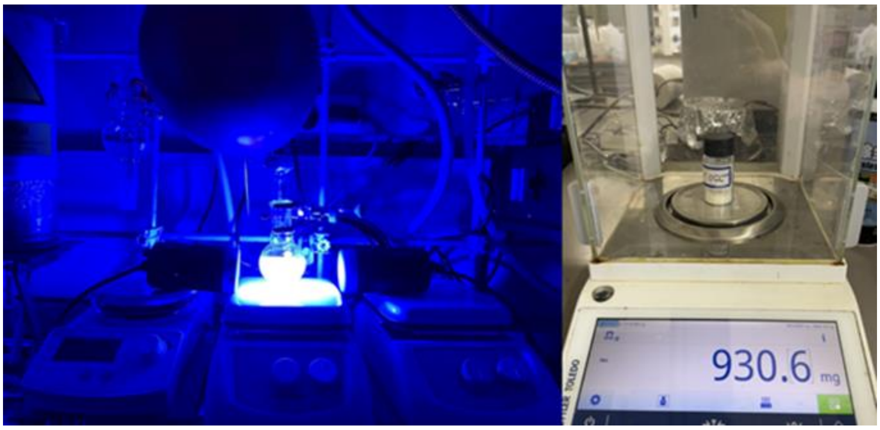

An overview of different products

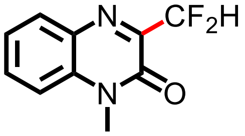

1a: yellow solid, 91% yield; ^{1}H NMR (400 MHz, $CDCl_3$) δ 8.01-7.99 (m, 1H), 7.68-7.64 (m, 1H), 7.42-7.34 (m, 2H), 6.96-6.93 (t, *J*=53.6 Hz, 1H), 3.70 (s, 3H); ^{13}C NMR (101 MHz, $CDCl_3$) δ 153.32, 153.32, 148.89, 148.66, 148.66, 134.12, 134.12, 132.84, 132.83, 132.04, 131.97, 131.49, 124.53, 124.53, 114.13, 114.13, 112.66, 110.25, 107.85, 107.85, 77.55, 77.24, 76.92, 29.11; ^{19}F NMR (376 MHz, $CDCl_3$) δ -125.1 (d, *J* = 58.8 Hz, 2F).

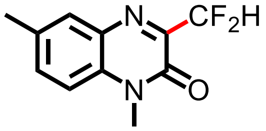

1b: yellow solid, 89% yield; ^{1}H NMR (400 MHz, DMSO-d_6) δ 7.87 – 7.62 (m, 1H), 7.59 – 7.25 (m, 2H), 7.07 (td, *J* = 53.3, 3.7 Hz, 1H), 3.62 (s, 3H), 2.46 (d, *J* = 35.5 Hz, 3H); ^{13}C NMR (101 MHz, $CDCl_3$) δ 153.6, 152.6, 147.5, 147.1, 144.5, 134.1,131.3, 130.3, 126.1, 114.1, 112.9, 110.4, 108.1; ^{19}F NMR (376 MHz, $CDCl_3$) δ -123.51, -123.66.

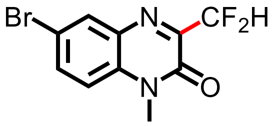

1c: yellow solid, 85% yield; ^{1}H NMR (400 MHz, $CDCl_3$) δ 8.14 (d, J = 2.2 Hz, 1H), 7.76 (dd, *J* = 8.9, 2.2 Hz, 1H), 7.28-7.26 (m, 1H), 6.92 (t, *J* = 53.6 Hz, 1H), 3.72 (s, 3H); ^{13}C NMR (101 MHz, $CDCl_3$) δ 152.8, 149.8 (t, *J* = 22.8 Hz), 135.6, 133.9, 133.3, 132.7, 117.1, 115.6, 112.3, 110.0 (t, *J* = 243.0 Hz), 29.5; ^{19}F NMR (376 MHz, $CDCl_3$) δ -124.59, -124.61.

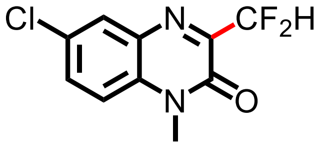

1d: yellow solid, 83% yield; ^{1}HNMR (400 MHz, $CDCl_3$) δ 7.98 (d, *J* = 2.4 Hz, 1H), 7.63 (dd, *J* = 9.0, 2.4 Hz, 1H), 7.32 (d, *J* = 9.0 Hz, 1H), 6.93 (t, *J* = 53.6 Hz, 1H), 3.72 (s, 3H); ^{13}C NMR (101 MHz, $CDCl_3$) δ153.6, 151.2, 149.9 (t, *J* = 22.8 Hz), 132.9, 132.5, 131.1, 130.8, 129.8, 115.2, 107.4 (t, *J* = 242.5 Hz); ^{19}F NMR (376 MHz, $CDCl_3$) δ -124.39, -124.61.

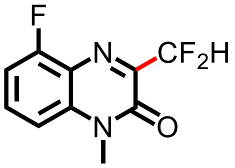

1e: orange solid, 87% yield; ^{1}H NMR (400 MHz, $CDCl_3$) 7.69 – 7.57 (m, 1H), 7.15 (t, *J* = 8.7 Hz, 2H), 6.90 (t, *J* = 53.5 Hz, 1H), 3.73 (s, 3H); ^{13}C NMR (101 MHz, $CDCl_3$) δ160.68, 158.63, 158.08, 157.33, 153.16, 150.21, 135.82, 133.67, 132.11, 122.66, 113.09, 110.77, 109.95, 108.27, 29.68. ^{19}F NMR (376 MHz, $CDCl_3$) δ -119.07, -121.29, -123.91.

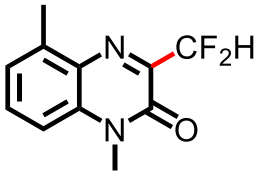

1f: yellow solid, 88% yield; ^{1}H NMR (400 MHz, $CDCl_3$) δ 7.49 (t, J = 8.0 Hz, 1H), 7.19 (s, 1H), 7.14 (d, *J* = 8.5 Hz, 1H), 6.86 (t, *J* = 53.8 Hz, 1H), 3.66 (s, 3H), 2.65 (s, 3H); ^{13}C NMR (101 MHz, $CDCl_3$) δ 153.26, 146.88, 146.64, 146.37, 140.50, 134.37, 132.56, 130.86, 125.79, 113.26, 111.75, 110.76, 108.35, 29.11, 17.55.; ^{19}F NMR (376 MHz, $CDCl_3$) δ -123.55, -123.71.

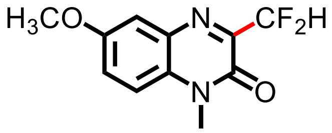

1g: white solid, 89% yield; ^{1}H NMR (400 MHz, $CDCl_3$) δ 7.43 (s, 1H), 7.31 (d, *J* = 1.6 Hz, 2H), 6.98 (t, *J* = 53.8 Hz, 1H), 3.90 (s, 3H), 3.73 (s, 3H); ^{13}C NMR (101 MHz, $CDCl_3$) δ 156.9, 153.2, 149.0 (t, *J* = 22.1 Hz), 132.7, 128.8, 122.5, 115.1, 112.3, 110.0 (t, *J* = 242.4 Hz), 55.6, 29.7; ^{19}F NMR (376 MHz, $CDCl_3$) δ -124.37, -124.52.

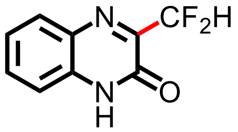

1h: white solid, 95% yield; ^{1}H NMR (400 MHz, DMSO-d_6) δ 12.85 (s, 1H), 7.89-7.87 (m, 1H), 7.70-7.64 (m, 1H), 7.40-7.35 (m, 2H), 6.94 (td, *J* = 53.1, 16.2 Hz, 1H); ^{13}C NMR (101 MHz, DMSO-d_6) δ 153.7, 152.4 (t, *J* = 22.2 Hz), 151.3, 141.7, 132.8, 130.0, 124.4, 116.3, 110.8 (t, *J* = 238.4 Hz); ^{19}F NMR (376 MHz, DMSO-d_6) δ -124.3 (d, *J* = 53.5 Hz, 2F).

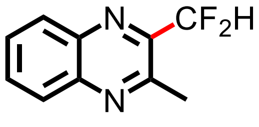

1i: white solid, 71% yield; ^{19}F NMR (376 MHz, DMSO-d_6) δ -117.3 (d, *J* = 53.5 Hz, 2F). Other spectral data of **1i** were consistent with previous reported data.[3]

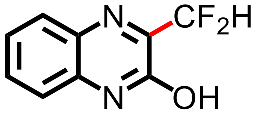

1j: white solid, 93% yield; ^{1}H NMR (400 MHz, DMSO-d_6) δ 12.9 (s, 1H), 7.90 (dd, *J* = 8.1, 1.3 Hz, 1H), 7.66 (td, *J* = 7.7, 1.4 Hz, 1H), 7.41-7.37 (m, 2H), 7.05 (t, *J* = 53.3 Hz, 1H); ^{19}F NMR (376 MHz, DMSO-d_6) δ -119.5 (d, *J* = 53.1 Hz, 2F).

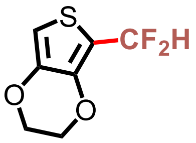

1k: colorless oil, 63% yield; ^{1}H NMR (400 MHz, $CDCl_3$) δ 6.88 (t, J = 55.3, Hz), 6.50 (s, 1H), 4.30-4.23 (m, 4H); ^{19}F NMR (376 MHz, $CDCl_3$) δ -105.24, -106.70. Other spectral data of 1k were consistent with previous reported data.

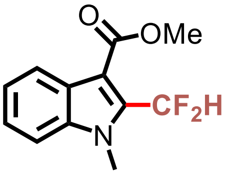

1l: white solid, 75% yield; ^{1}H NMR (400 MHz, $CDCl_3$) δ 8.18 (d, J = 8.0 Hz, 1H), 7.93 (t, J = 52.0 Hz, 1H), 7.40-7.31 (m, 3H), 3.98 (s, 6H); ^{13}C NMR (101 MHz, $CDCl_3$) δ 165.3, 137.7, 135.6 (t, J = 21.6 Hz), 125.2, 123.1, 122.9, 122.2, 110.2, 109.0 (t, J = 235.0 Hz), 107.5 (t, J = 6.4 Hz), 51.7, 32.0; ^{19}F NMR (376 MHz, $CDCl_3$) δ -113.82, -113.87.

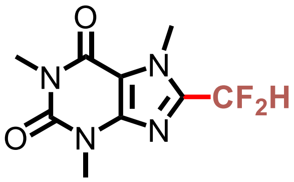

1m: white solid, 75% yield; ^{1}H NMR (400 MHz, DMSO-d_6) δ 7.33 (t, J = 52.9 Hz, 1H), 3.39 (s, 3H), 3.32 (s, 3H), 3.20 (s, 3H); ^{13}C-NMR (101 MHz, DMSO-d_6) δ 155.5, 151.5, 149.3 (t, J = 27.0 Hz), 147.2, 109.1 (t, J = 237.0 Hz), 33.1, 30.1, 29.8, 28.3; ^{19}F NMR (376 MHz, DMSO) δ -115.40.

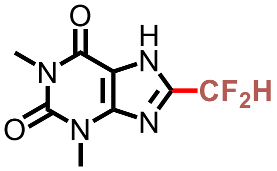

1n: white solid, 71% yield; ^{1}H NMR (400 MHz, DMSO-d_6) δ 14.6 (s, 1H), 7.13 (t, J = 52.9 Hz, 1H), 3.44 (s, 3H), 3.25 (s, 3H); ^{19}F NMR (376 MHz, DMSO-d_6) δ -115.6 (d, J = 53.1 Hz, 2F).

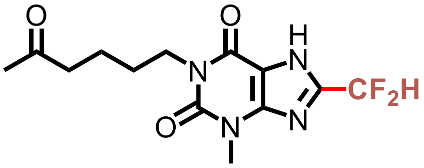

1o: white solid, 53% yield; ^{1}H NMR (400 MHz, $CDCl_3$) δ 6.71 (t, J = 52.2 Hz, 1H), 4.09 (s, 3H), 4.02-3.98 (m, 2H), 3.49 (s, 3H), 2.47 (t, J = 6.9 Hz, 2H), 2.09 (s, 3H), 1.69-1.58 (m, 4H); ^{13}C NMR (101 MHz, $CDCl_3$) δ 208.8, 155.5, 151.3, 147.1, 142.9 (t, J = 26.5 Hz), 109.8 (t, J = 237.0 Hz), 109.6, 43.3, 41.2, 33.0, 29.9, 27.5, 21.2; ^{19}F NMR (376 MHz, $CDCl_3$) δ -115.40.

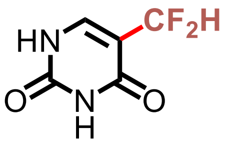

1p: white solid, 61% yield; ^{1}H NMR (400 MHz, DMSO-d_6) δ11.43 (s, 1H), 7.79 (s, 1H), 6.67 (t, *J* = 54.6 Hz, 1H); ^{13}C NMR (101 MHz, DMSO-d_6) δ 162.1 (t, *J* = 3.1 Hz), 151.3, 143.1 (t, *J* = 7.5 Hz), 112.7 (t, *J* = 233.4 Hz), 106.4 (t, *J* = 23.1 Hz); ^{19}F NMR (376 MHz, DMSO-d_6) δ -114.90 (d, *J* = 54.7 Hz, 2F).

Characterization

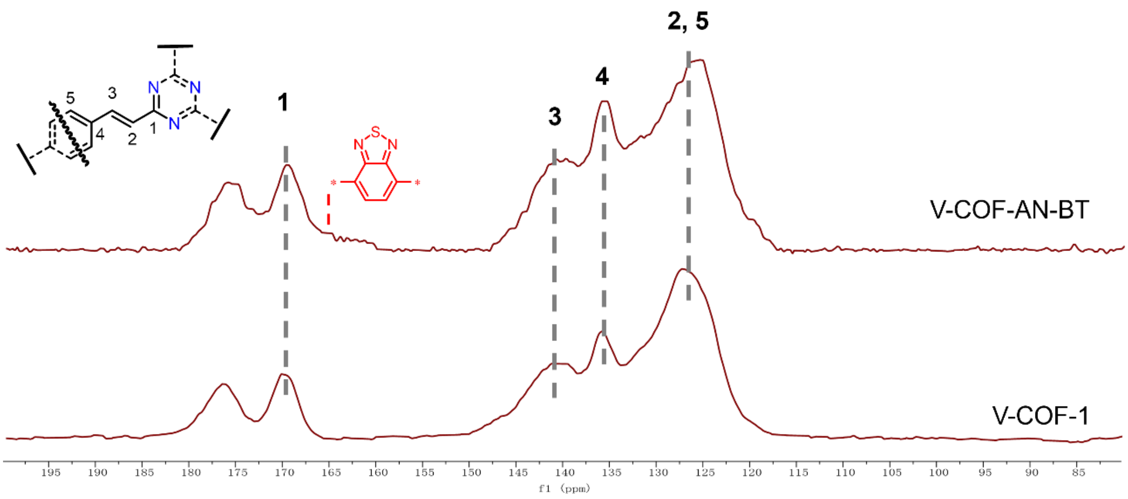

Figure. S1 Solid-state ^{13}C CP-MAS NMR of V-COF-1 and V-COF-AN-BT.

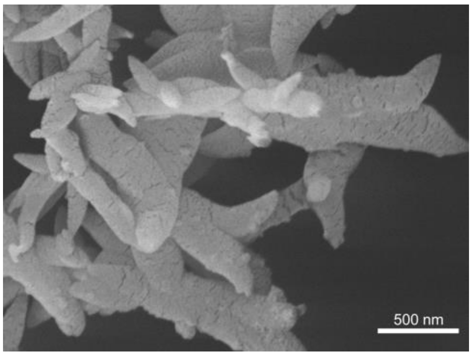

Figure. S2 SEM image of V-COF-AN-BT.

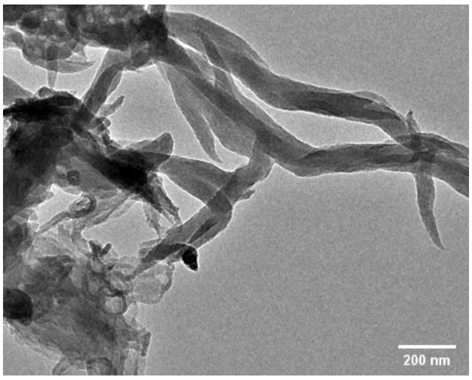

Figure. S3 TEM image of V-COF-AN-BT.

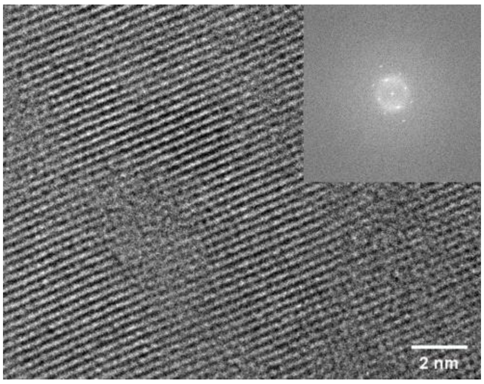

Figure. S4 High-resolution TEM image of V-COF-AN-BT magnified, Insets: fast Fourier transformed (FFT) image.

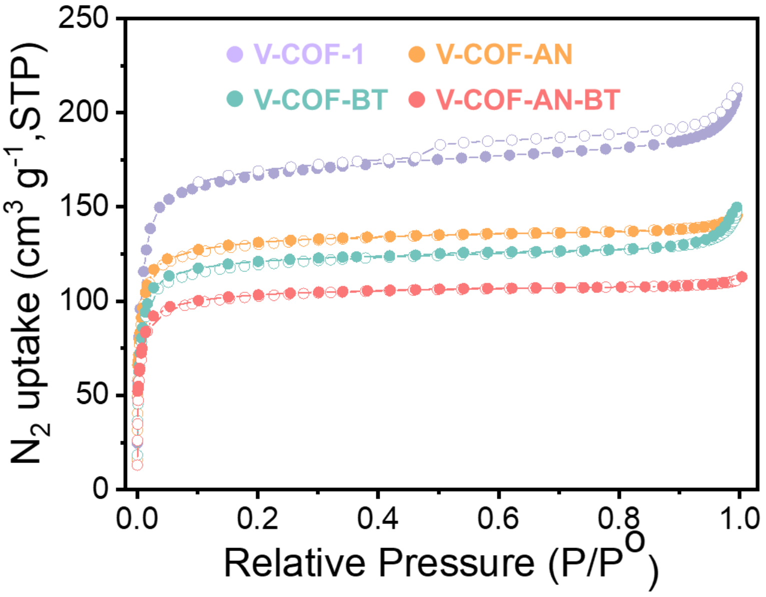

Figure. S5 Nitrogen sorption and desorption isotherms of COFs.

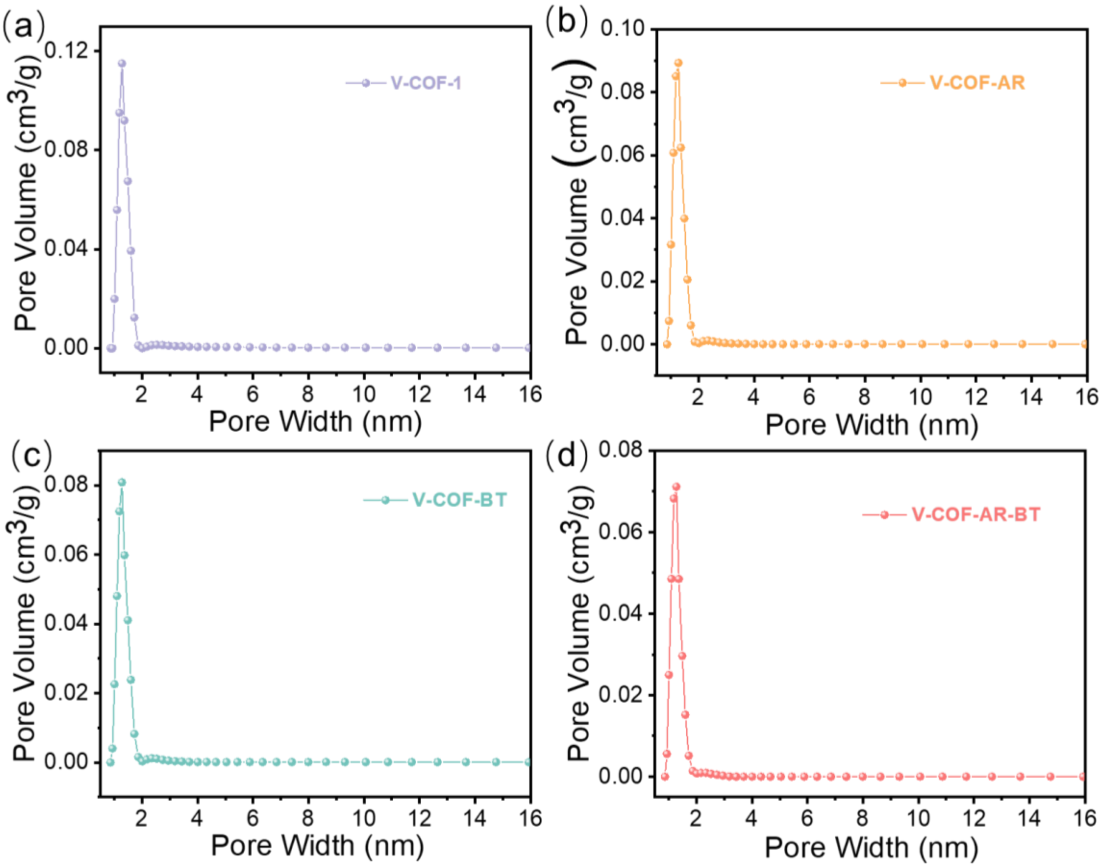

Figure. S6 Pore size distributions of COFs.

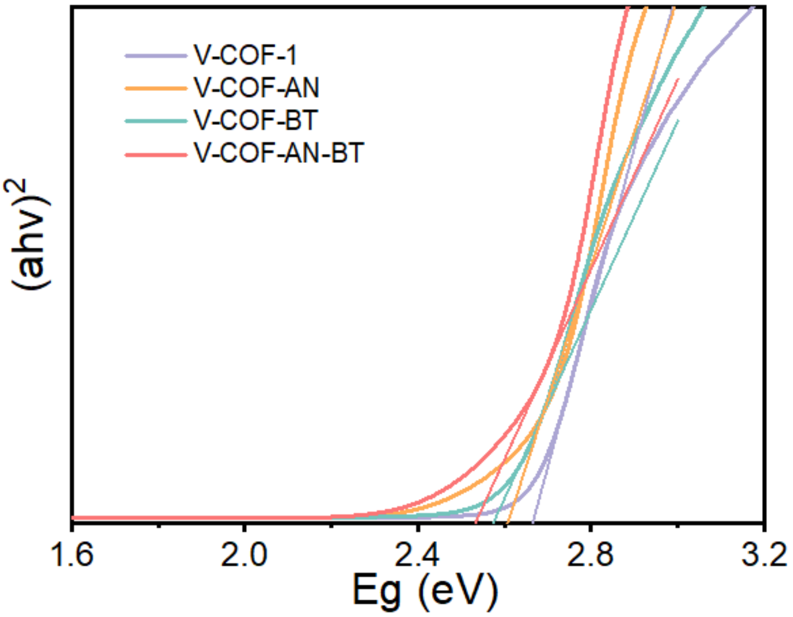

Figure. S7 Kubelka-Munk transformed UV/Vis reflectance spectra of COFs

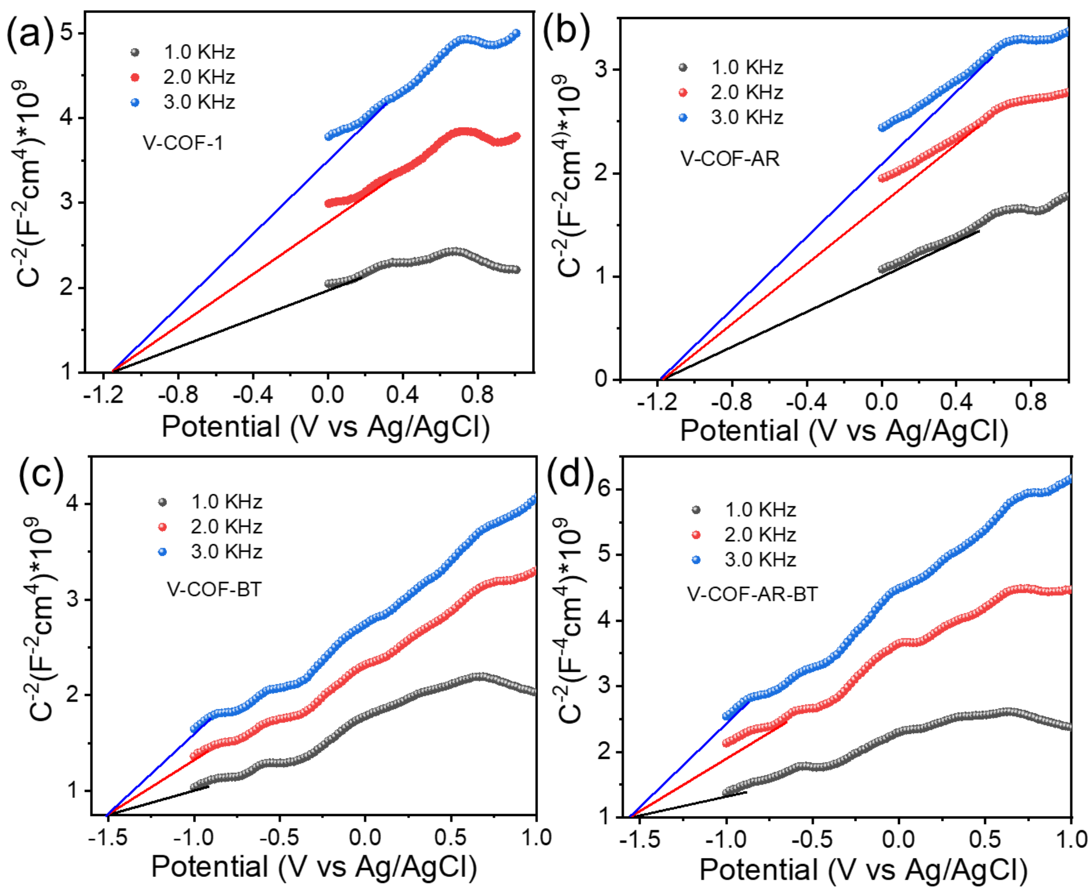

Figure. S8 Mott−Schottky plot and flat band potentials of the COFs.

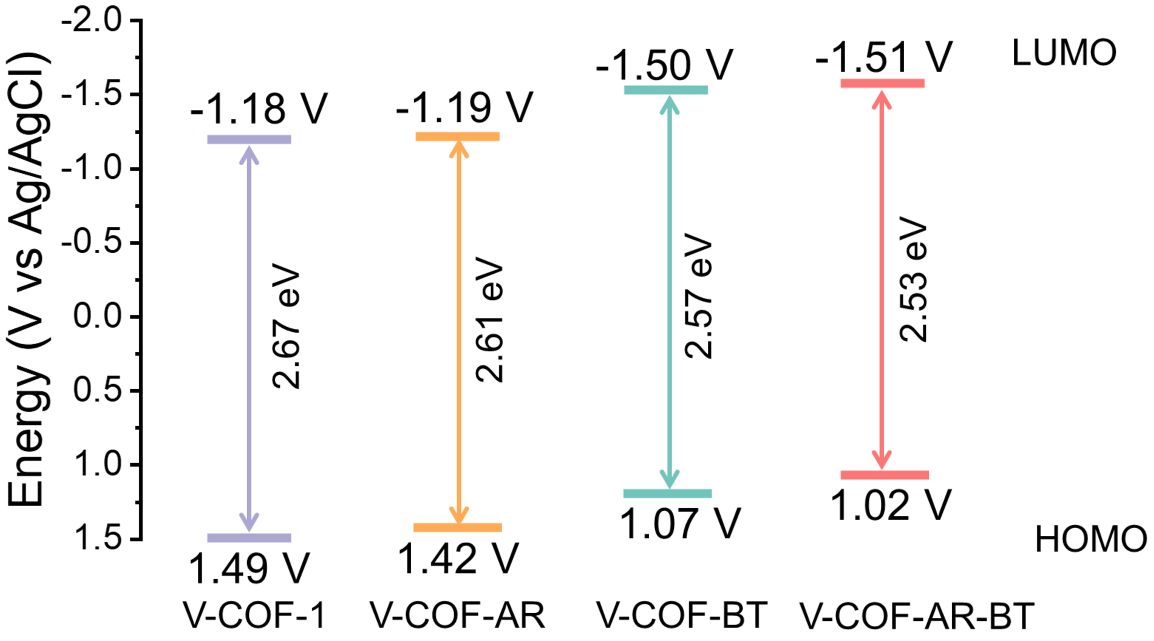

Figure. S9 HOMO/LUMO band positions of the COFs.

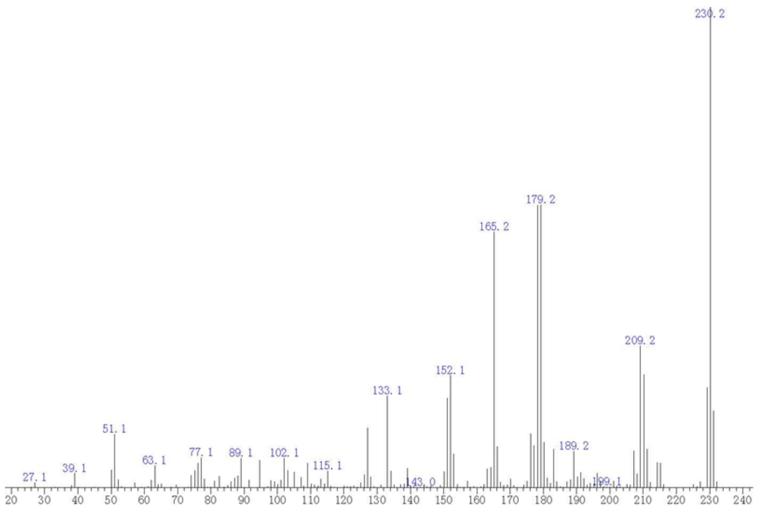

Figure. S10 Radical trapping experiment for **1a** and $NaSO_2CF_2H$ under standard conditions with ethene-1,1-diyldibenzene (2.0 equiv).

The radical trapping experiments were conducted with and $NaSO_2CF_2H$ under the standard conditions with a trapping agent 1,1-diphenylethylene (2.0 equiv) to capture the radical intermediate expected in our system, and the products were detected by HRMS techniques. Figure S10 showed that 1,1-diphenylethylene, the most common trapping agent, captured diarylmethane radical with 1,1-diphenylethylene-trapped (3,3-difluoroprop-1-ene-1,1-diyl)dibenzene observed. HRMS (ESI): compound 17, HRMS (ESI) calcd for $C_{15}H_{13}F_2$ $[M+H]^+$: 231.0980, found: 230.2.

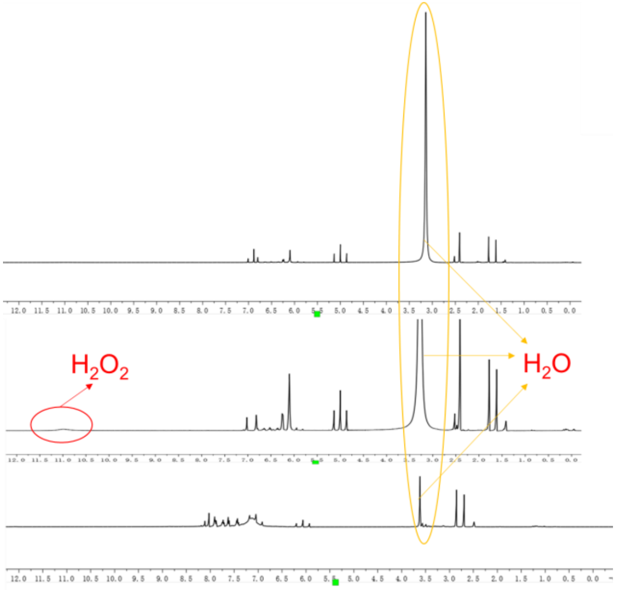

Figure. S11 ^1H NMR spectrum of detection of hydrogen peroxide.

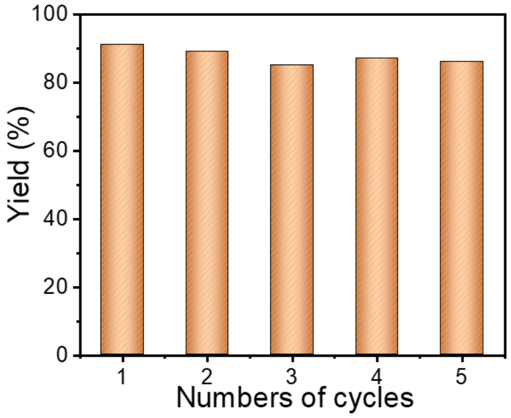

Figure. S12 Recyclability tests of V-COF-AN-BT in photocatalytic reaction.

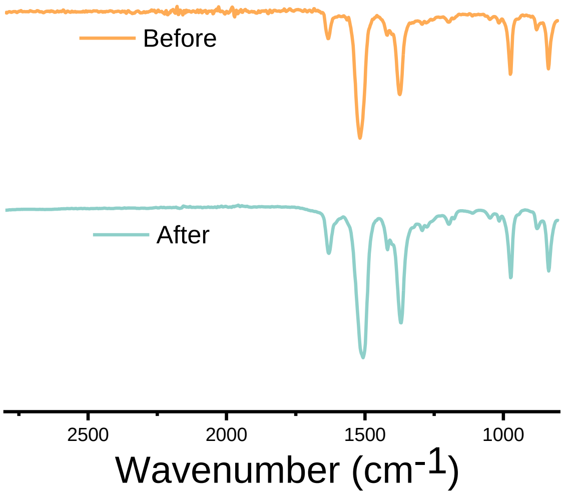

Figure. S13 FT-IR spectra of V-COF-AN-BT and recovered after 5 cycles.

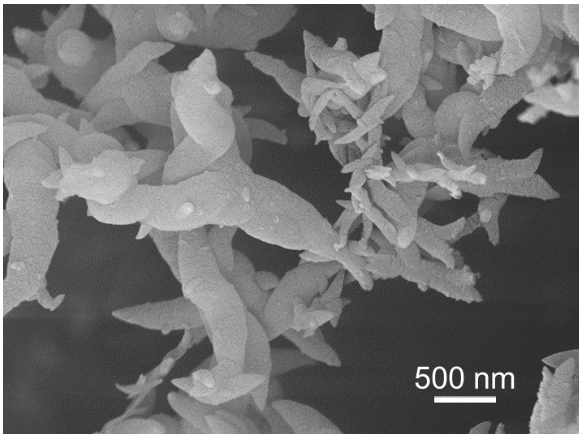

Figure. S14 SEM images of V-COF-AN-BT recovered after 5 cycles.

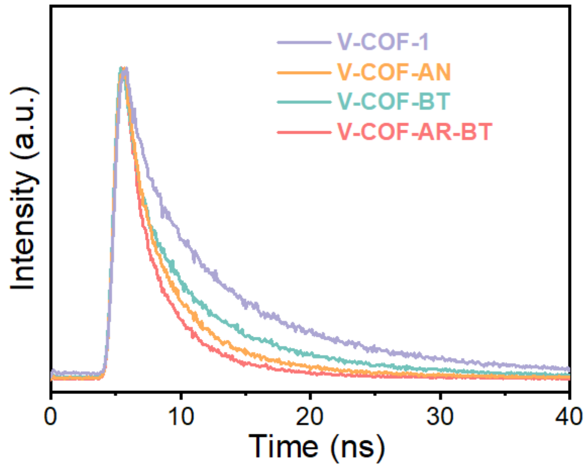

Figure.S15. Time-resolved PL spectra of the COFs.

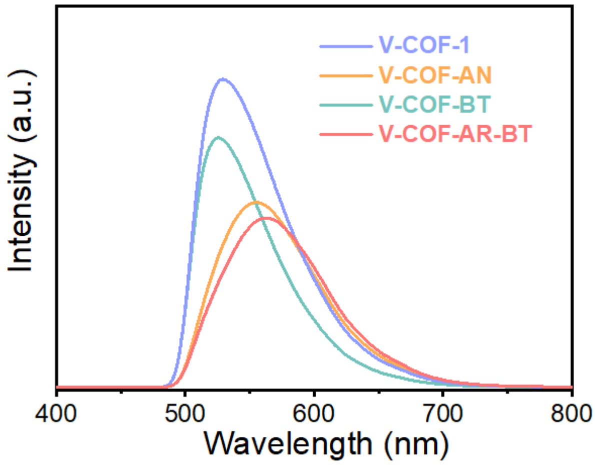

Figure.S16 Steady state photoluminescence (PL) spectra with λ_{exc} = 375 nm of the COFs.

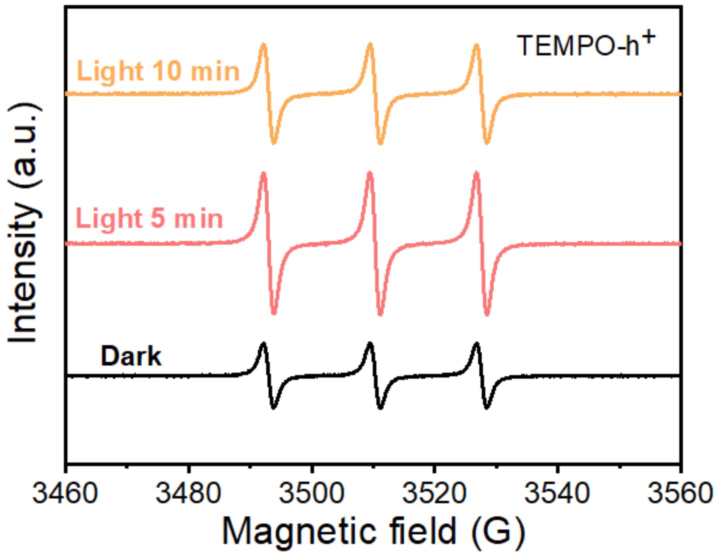

Figure.S17 ESR spectrometer of holes (TEMPO-h^+) in acetonitrile dispersion of V-COF-AN-BT at dark, 5 min irradiation, and 10 min irradiation.

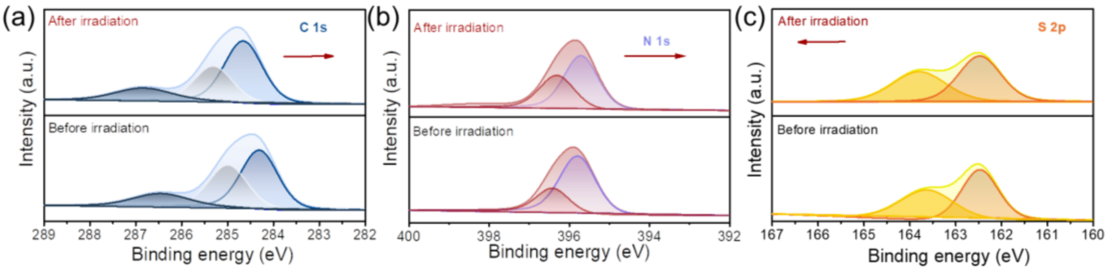

Figure. S18 *In situ* XPS spectra of (a) C 1s; (b) N 1s; and (c) S 2p for the V-COF-AN-BT.

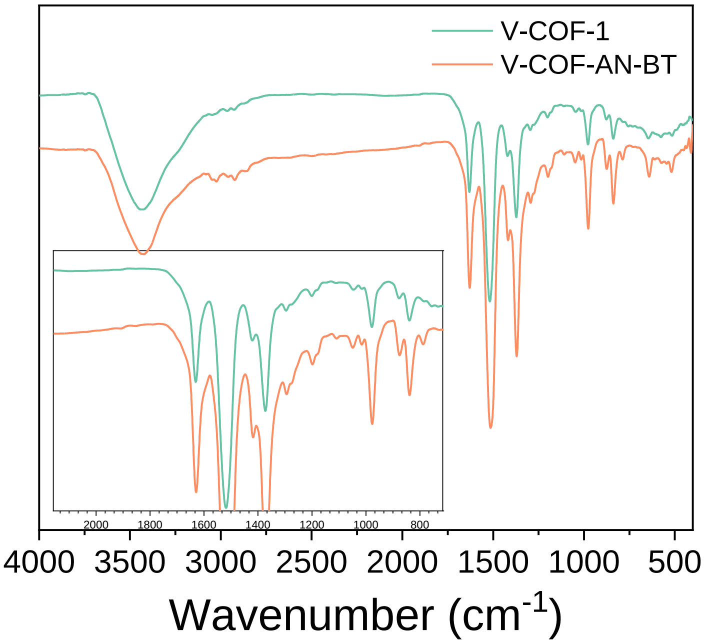

Figure. S19 FT-IR spectra of V-COF-1 and V-COF-AN-BT prepared by KBr disc method; Zoom in: 2000-800 cm^{-1}.

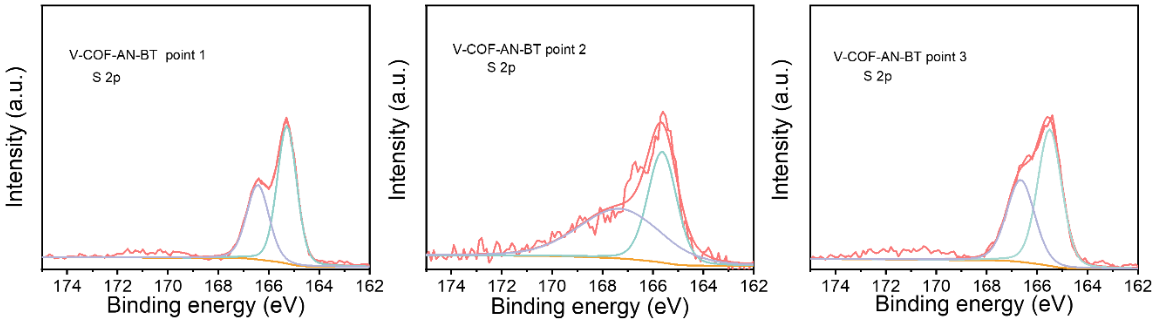

Figure. S20 X-ray photoelectron spectroscopy (XPS) analysis was conducted on V-COF-AN-BT to investigate its surface chemical composition.

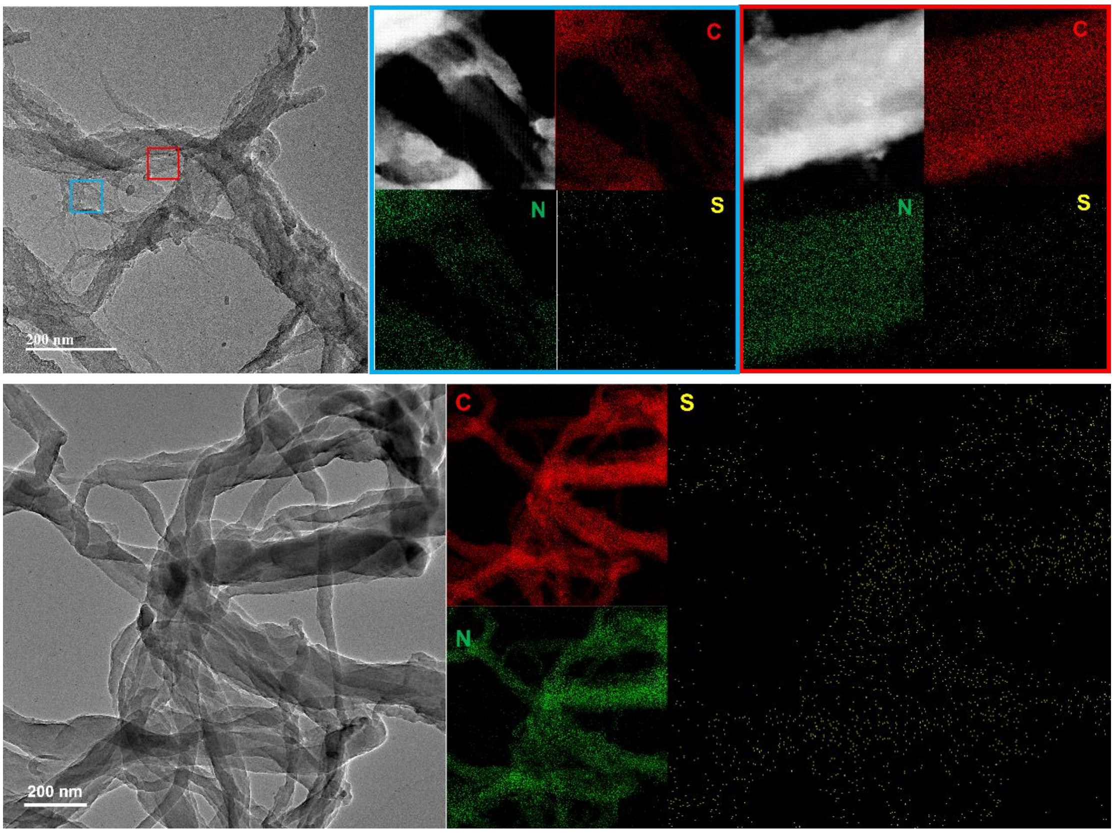

Figure. S21 Scanning transmission electron microscopy (STEM) and energy dispersive X-ray spectroscopy (EDS).

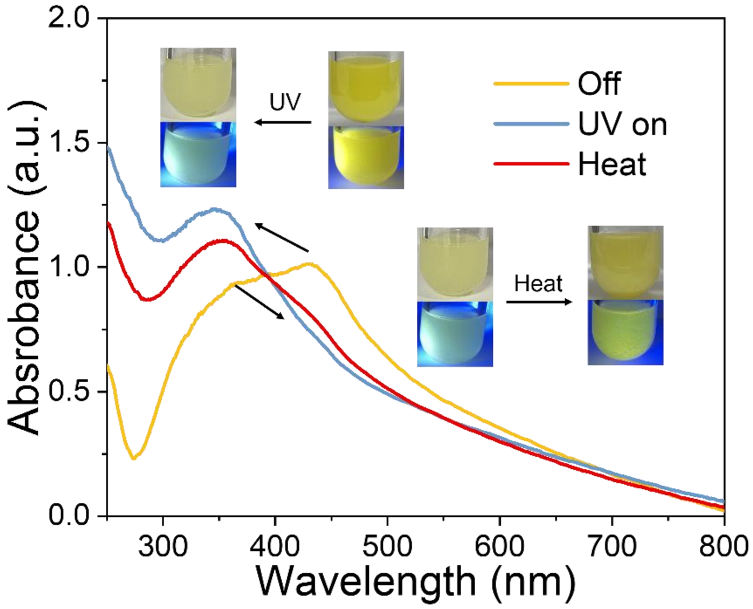

Figure. S22 Photochromic progress of V-COF-AN-BT (measurement methods were taken from previous literature[2, 4]).

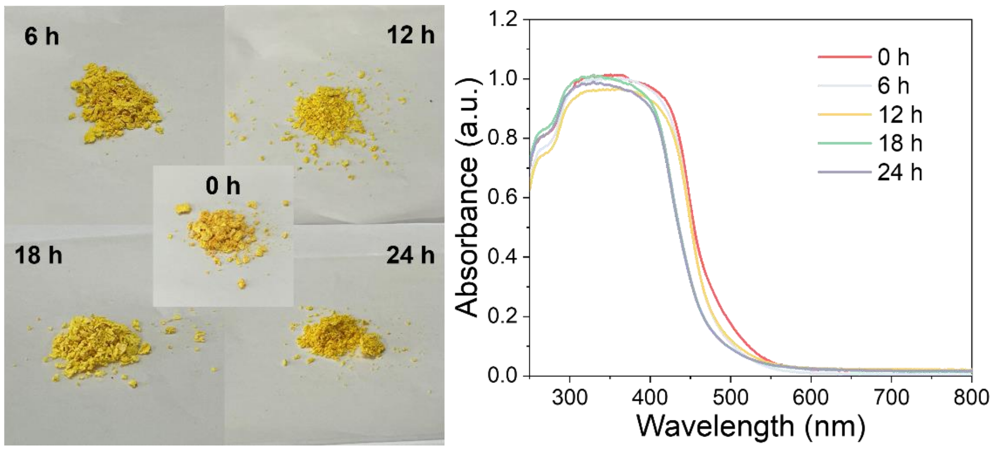

Figure. S23 The photos of V-COF-AN-BT during photocatalytic reaction.

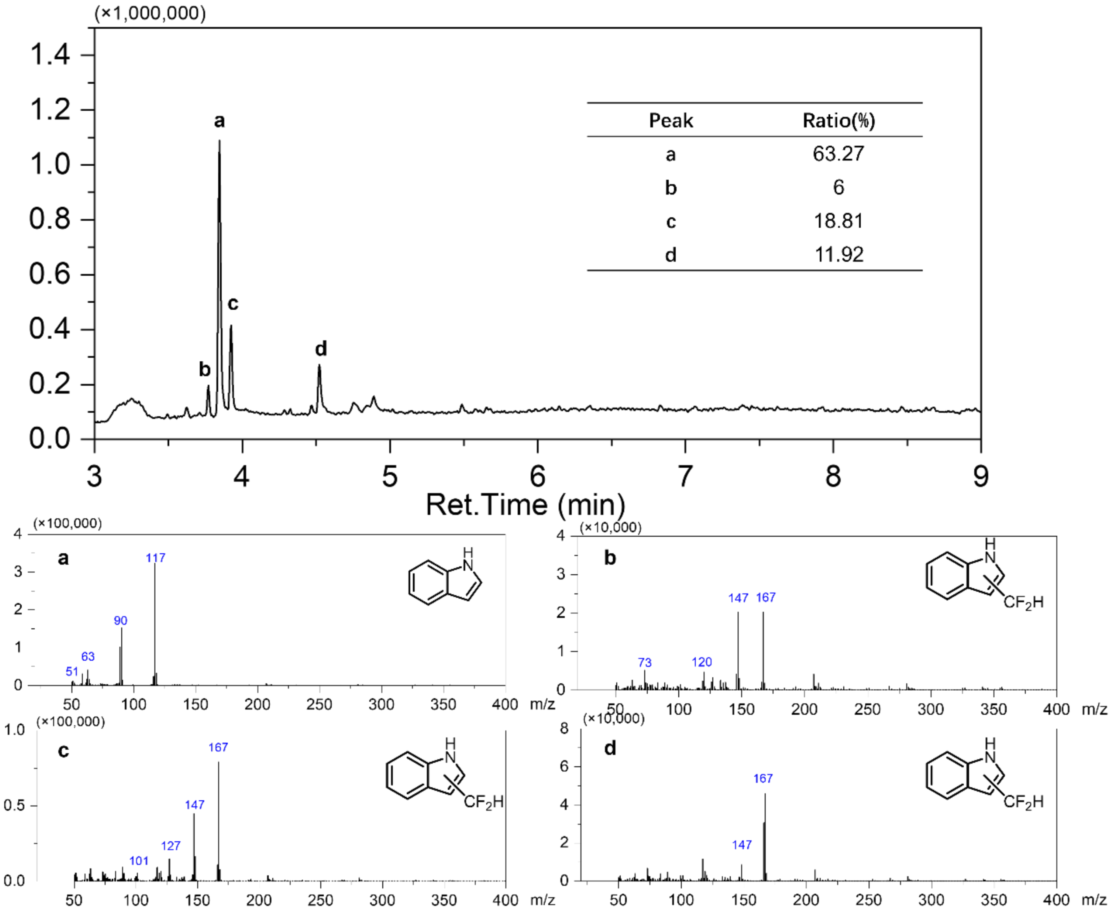

Figure. S24 The GC-MS spectra of V-COF-AN-BT photocatalyzed indole difluoromethylation.

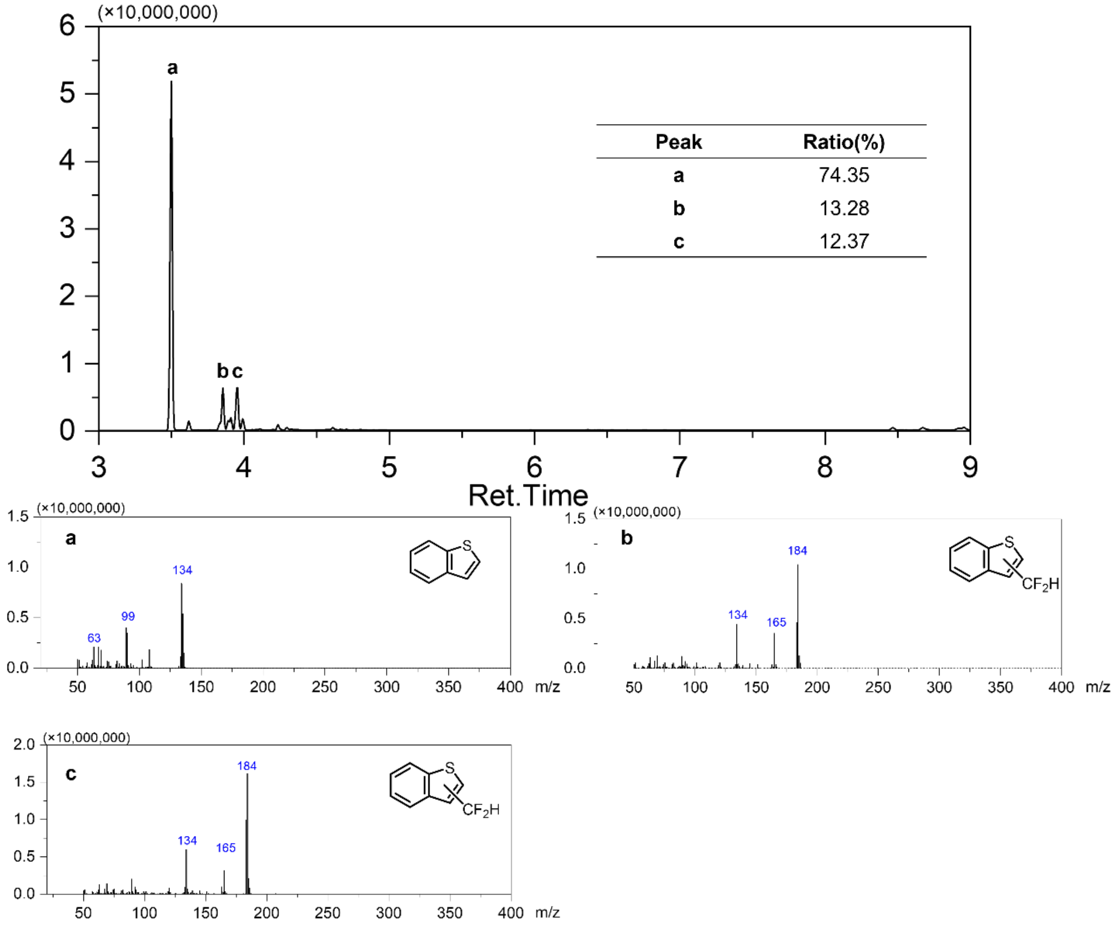

Figure. S25 The GC-MS spectra of V-COF-AN-BT photocatalyzed thianaphthene difluoromethylation.

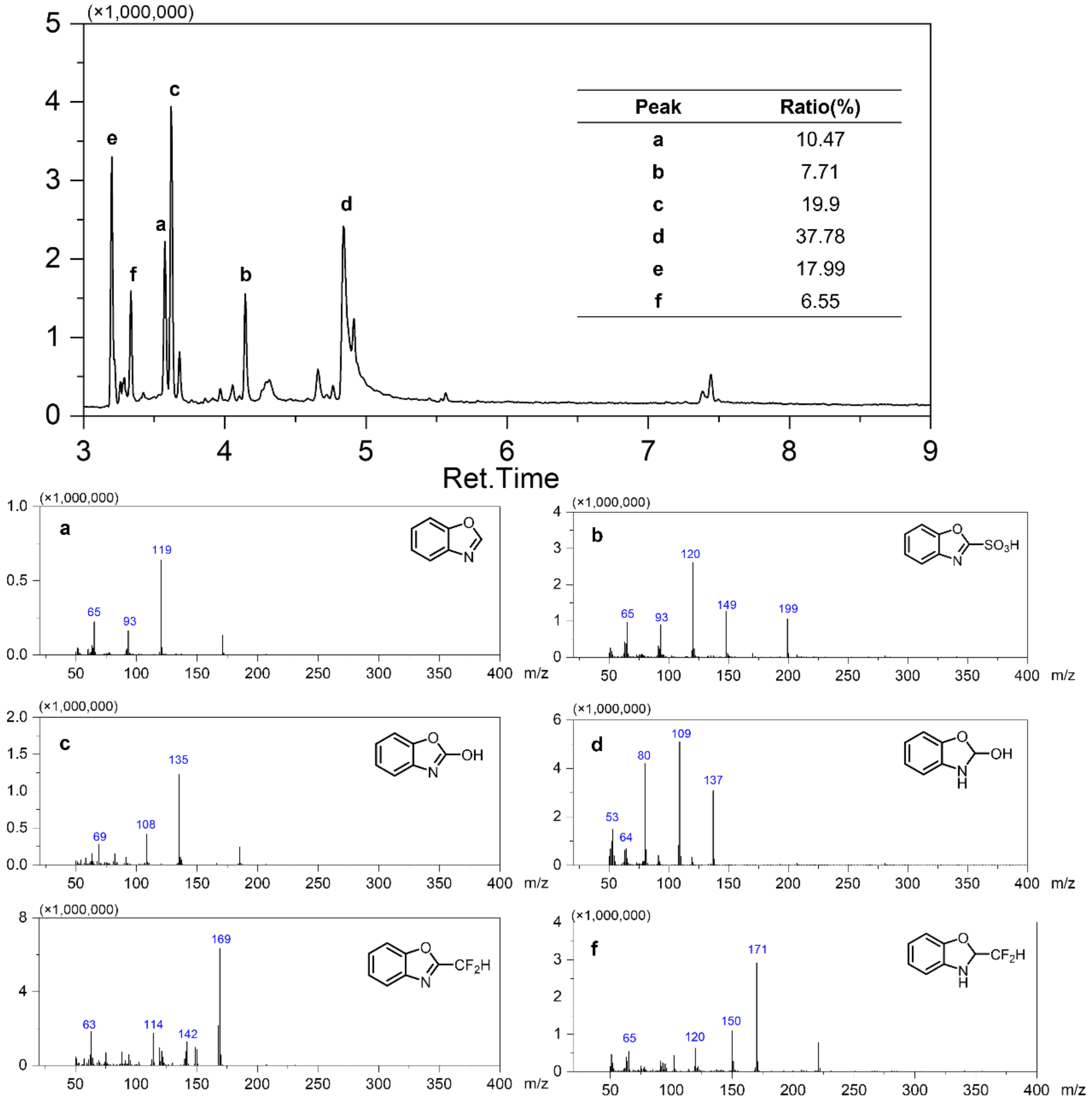

Figure. S26 The GC-MS spectra of V-COF-AN-BT photocatalyzed benzoxazole difluoromethylation.

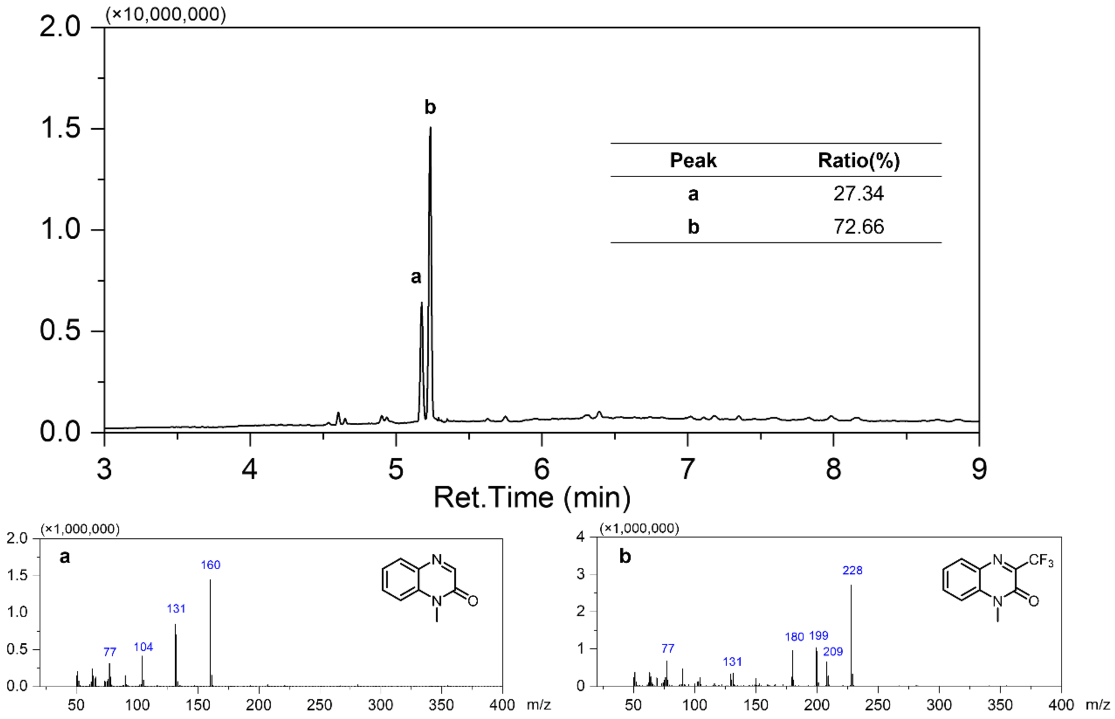

Figure. S27 The GC-MS spectra of V-COF-AN-BT photocatalyzed quinoxalin-2(1*H*)-ones trifluoromethylation with $NaSO_2CF_3$.

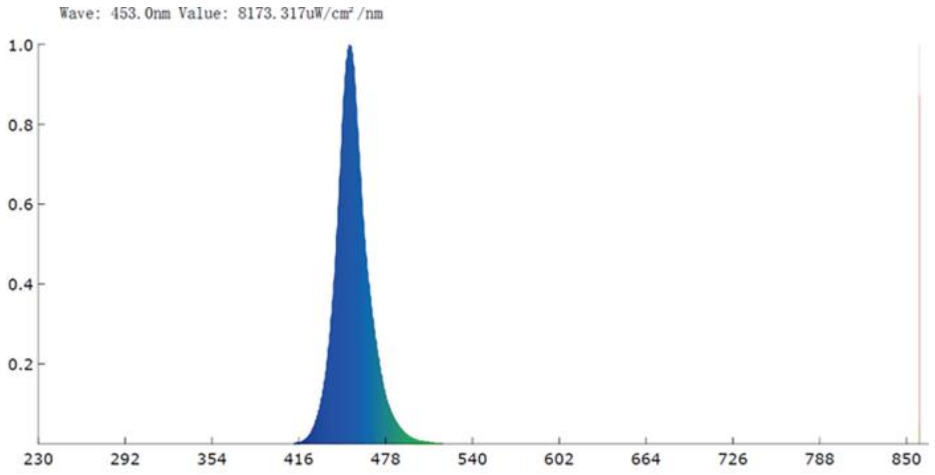

Figure. S28 The information of blue LEDs

Table S1 Some reported materials on photocatalytic direct C-H difluoromethylation

Photocatalyst	Reaction	Yield
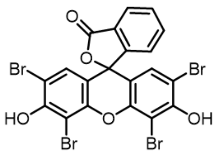	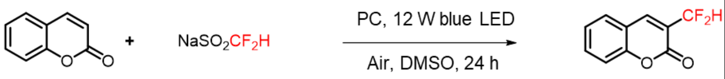	33-82%[5]

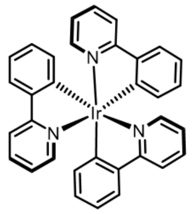	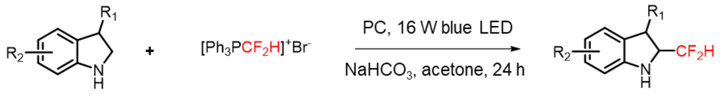	15-47%[6]
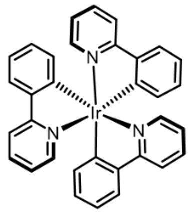	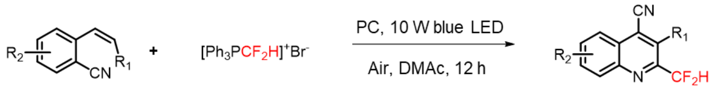	25-83%[7]
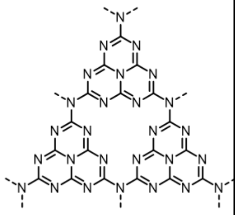	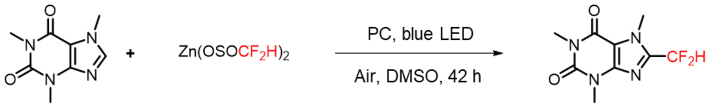	54%[8]
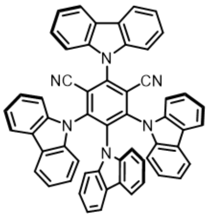	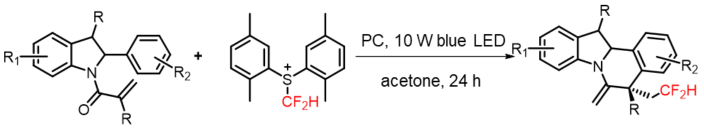	34-66%[9]
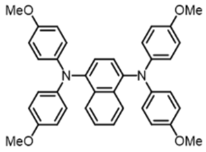	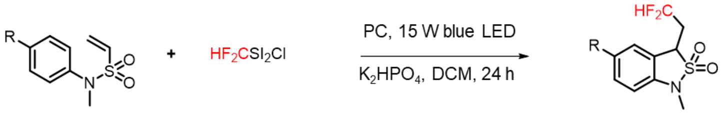	80-86%[10]
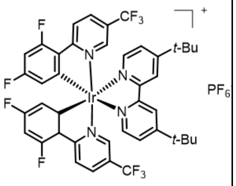	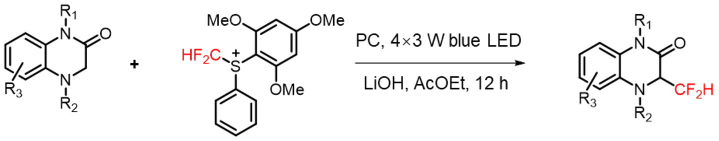	34-66%[11]
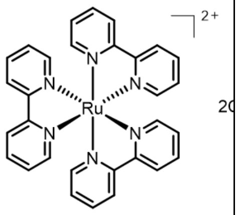	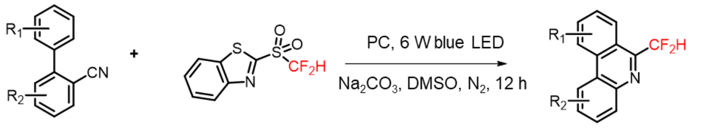	20-82%[12]

NMR spectra of products

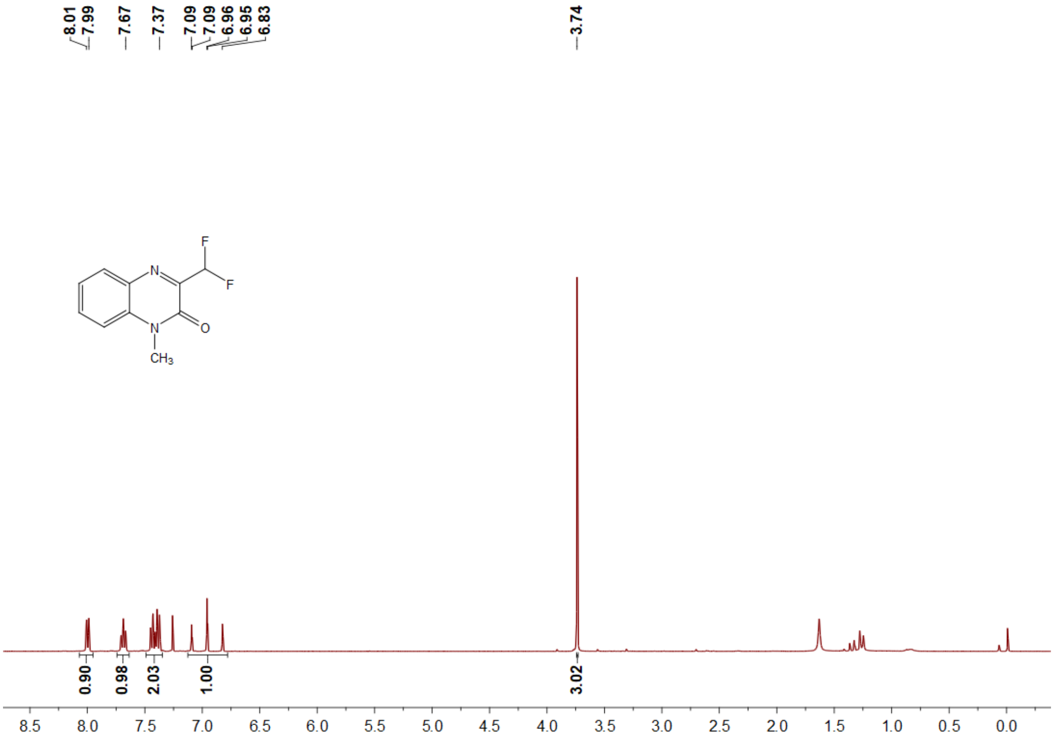

The ^{1}H-NMR Spectrum of **1a**.

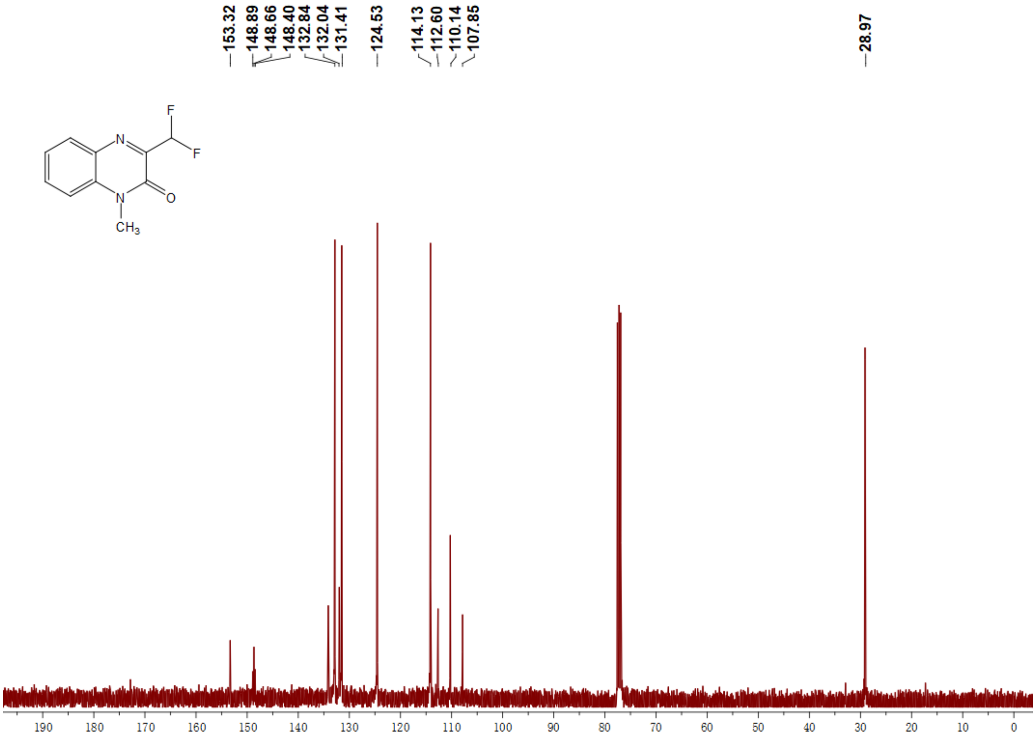

The ^{13}C-NMR Spectrum of **1a**.

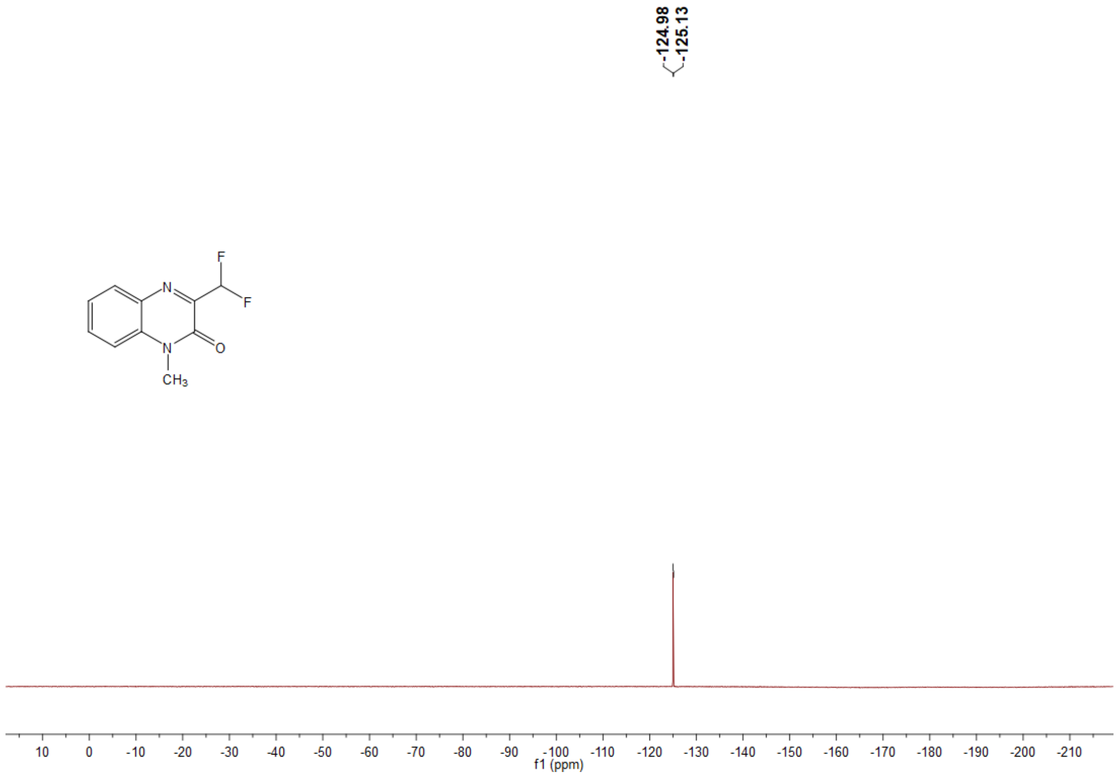

^{19}F NMR Spectrum of **1a**.

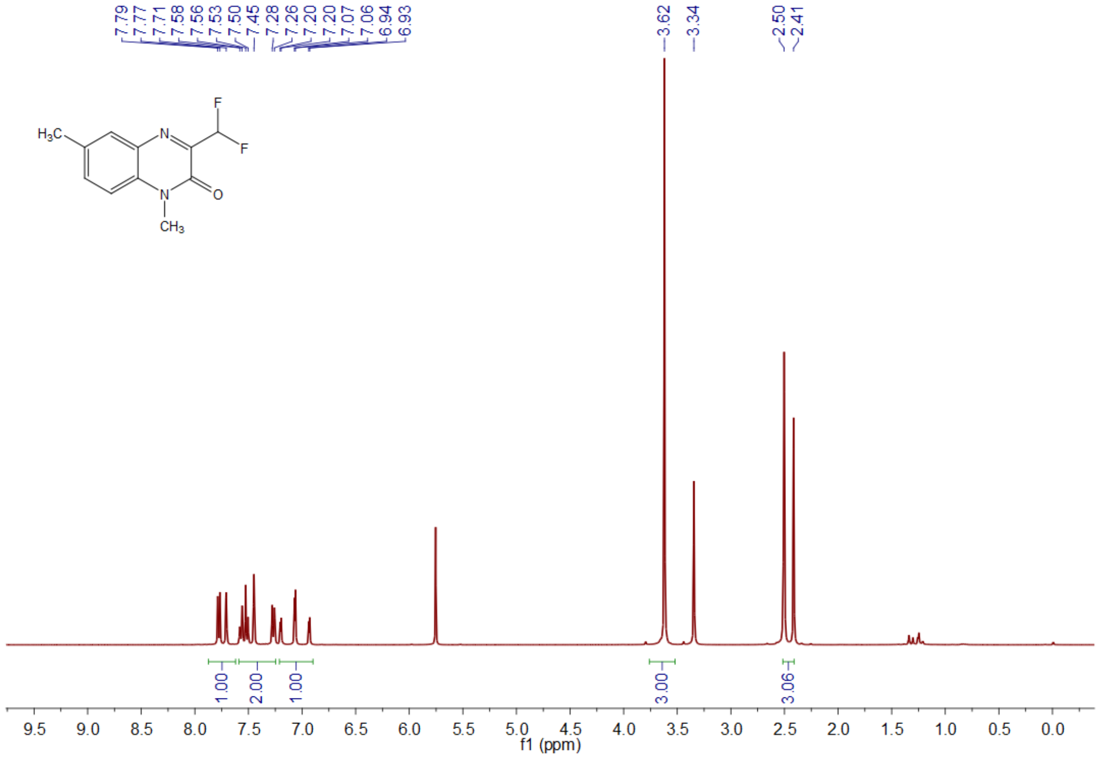

The ^{1}H-NMR Spectrum of **1b**.

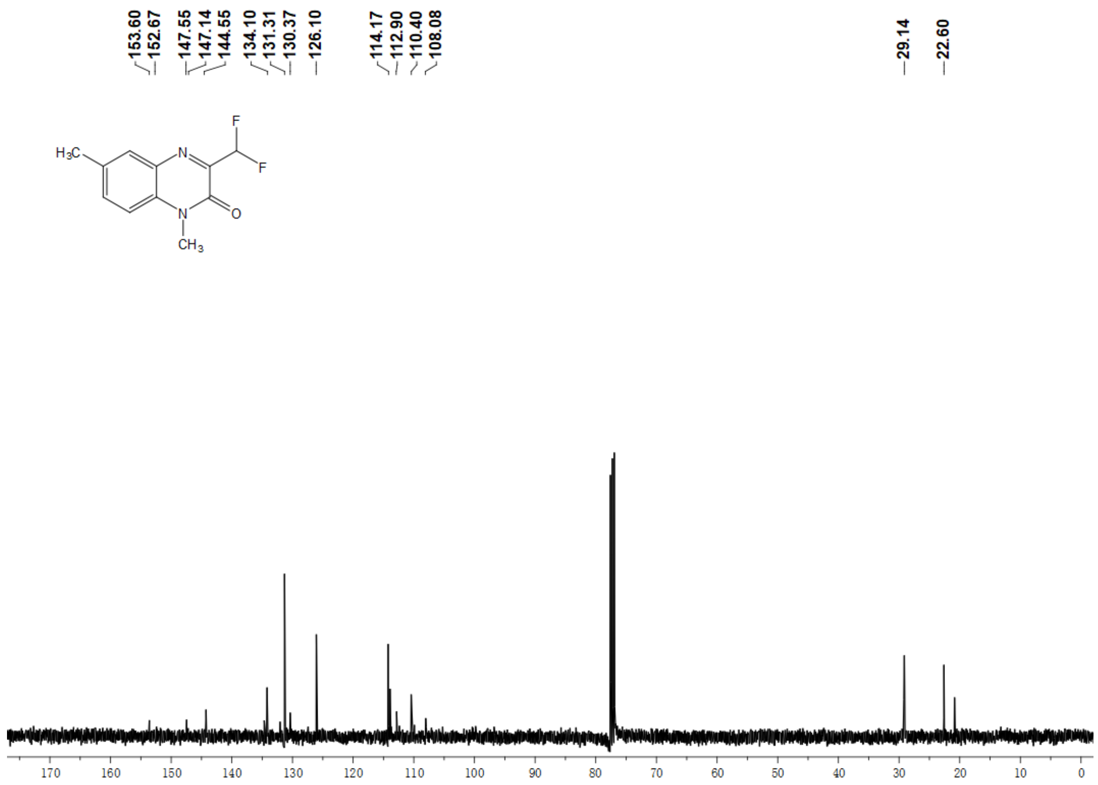

The ^{13}C-NMR Spectrum of **1b**.

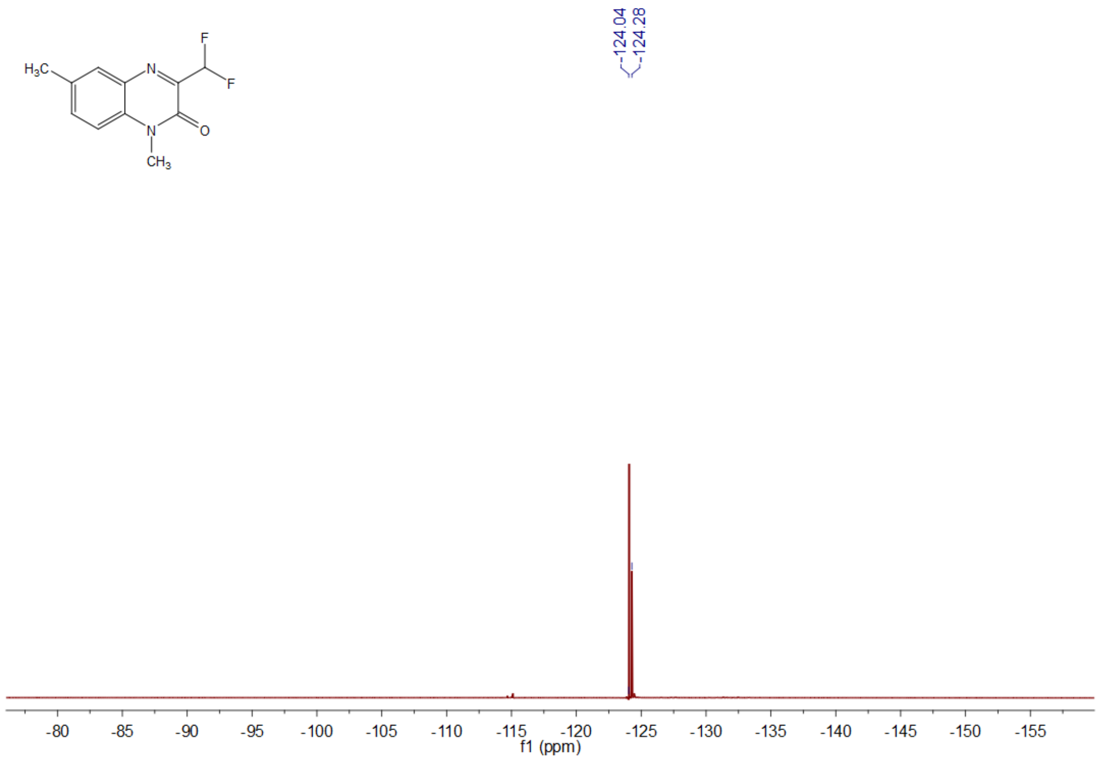

^{19}F NMR Spectrum of **1b**.

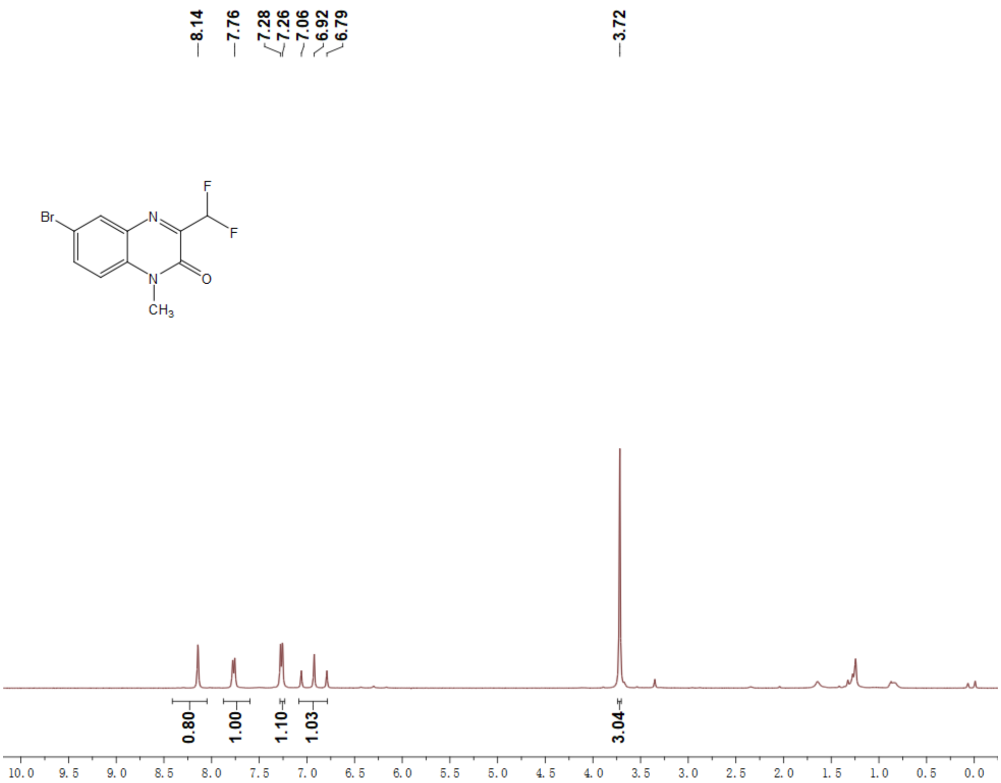

The ^{1}H-NMR Spectrum of **1c**.

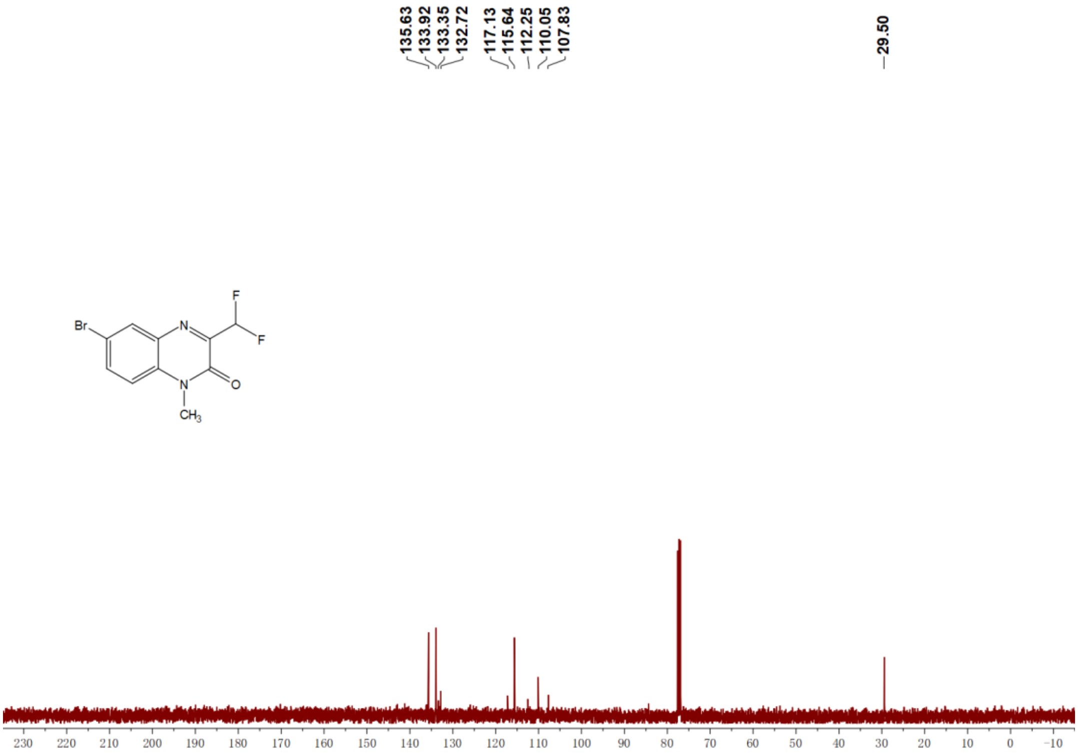

The ^{13}C-NMR Spectrum of **1c**.

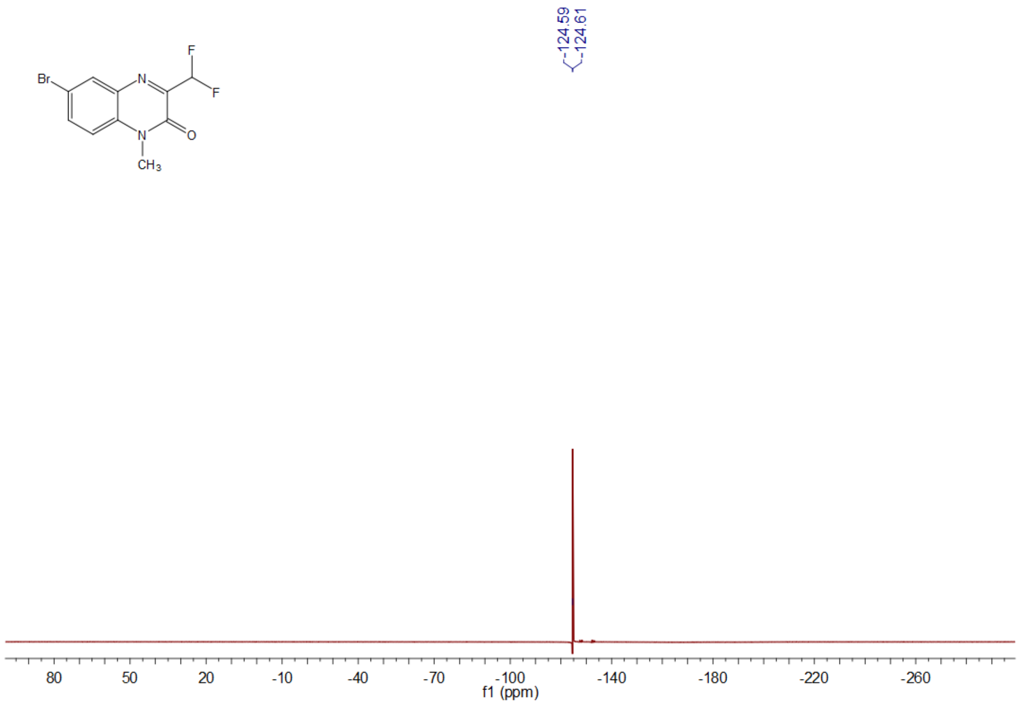

The ^{19}F-NMR Spectrum of **1c**.

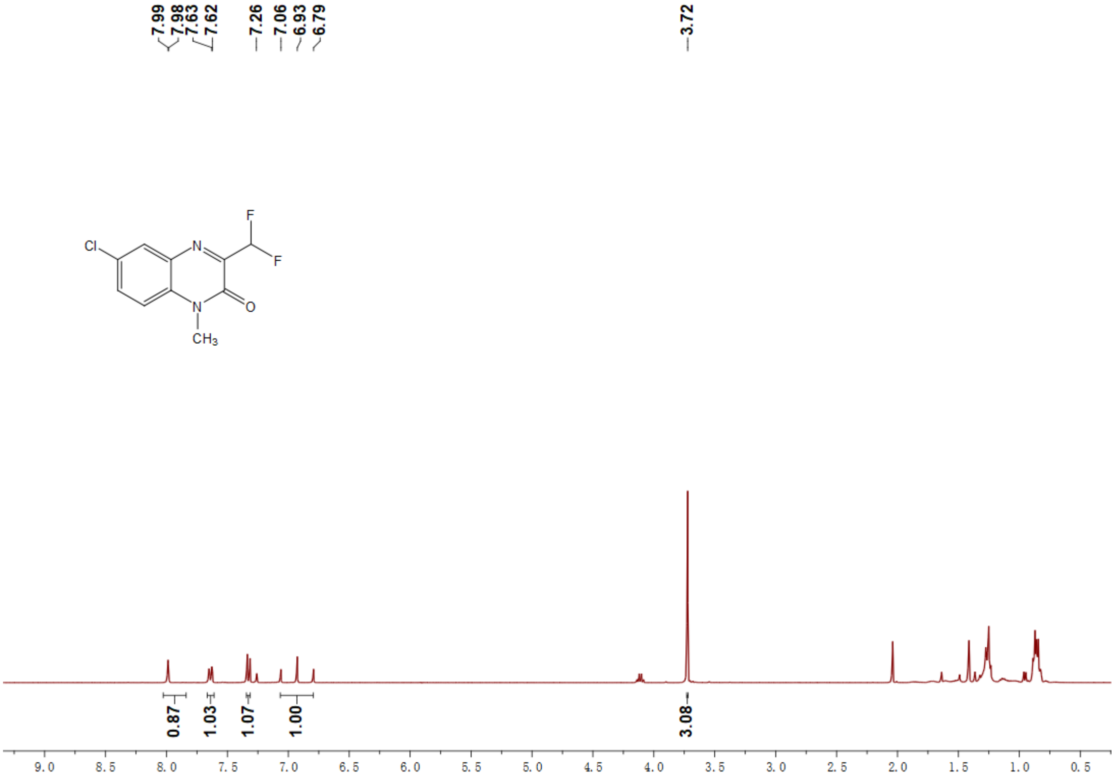

The ^{1}H-NMR Spectrum of **1d**.

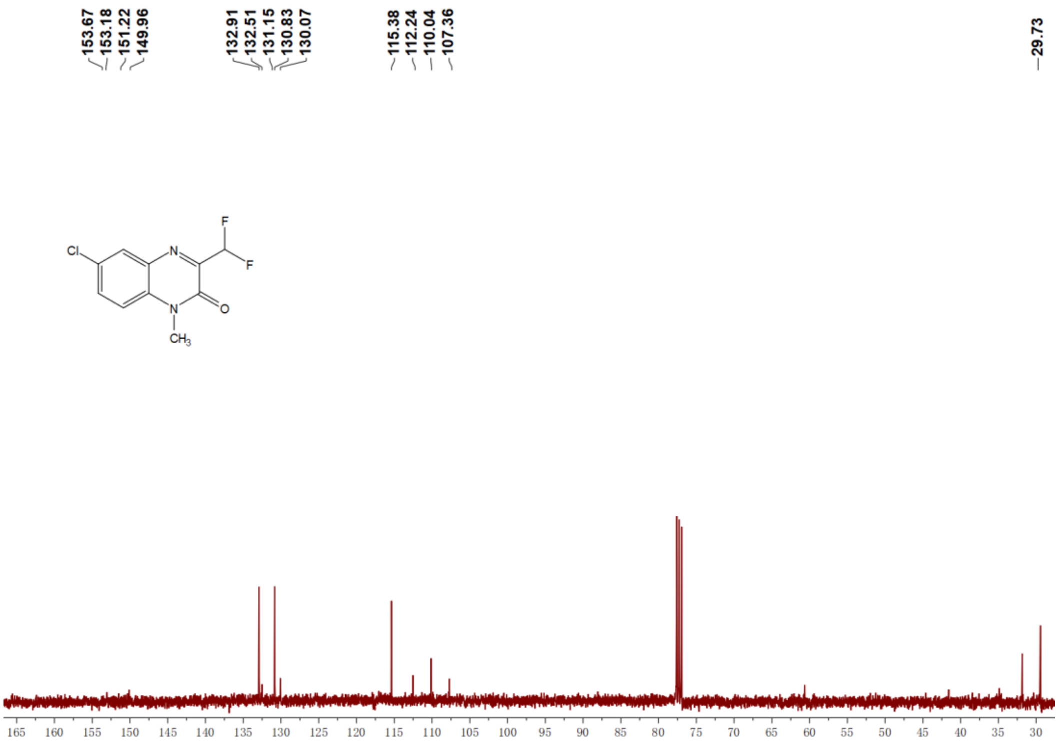

The ^{13}C-NMR Spectrum of **1d**.

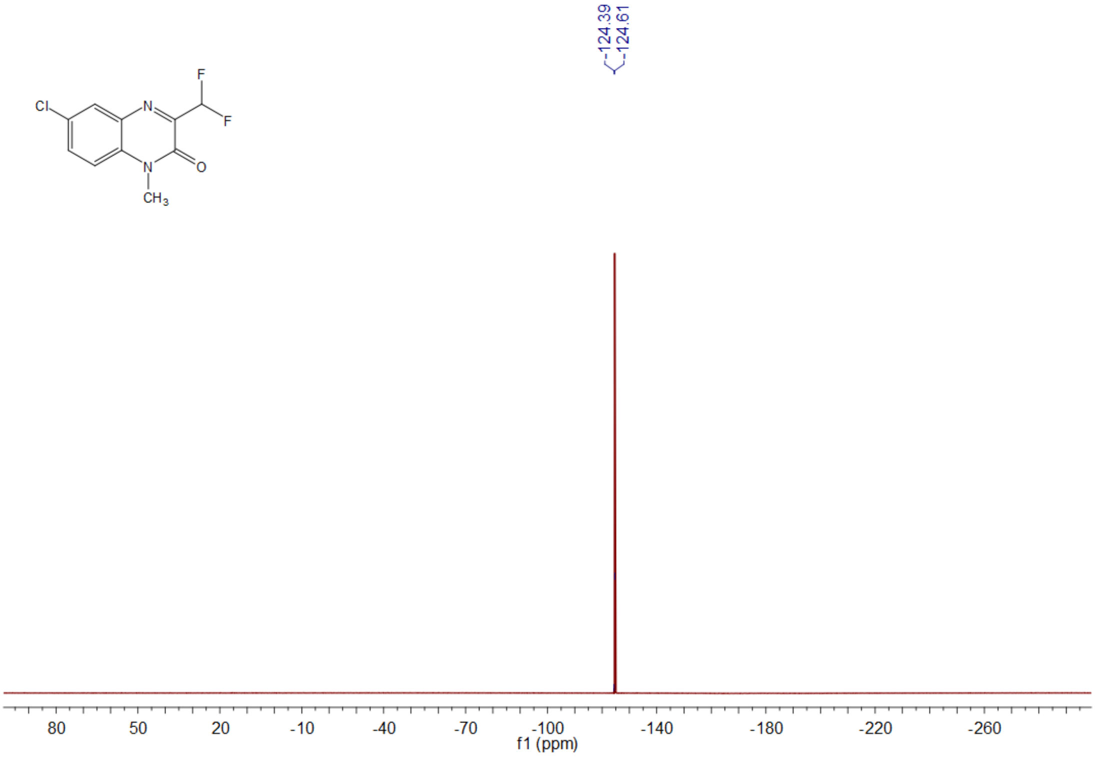

The ^{19}F-NMR Spectrum of **1d**.

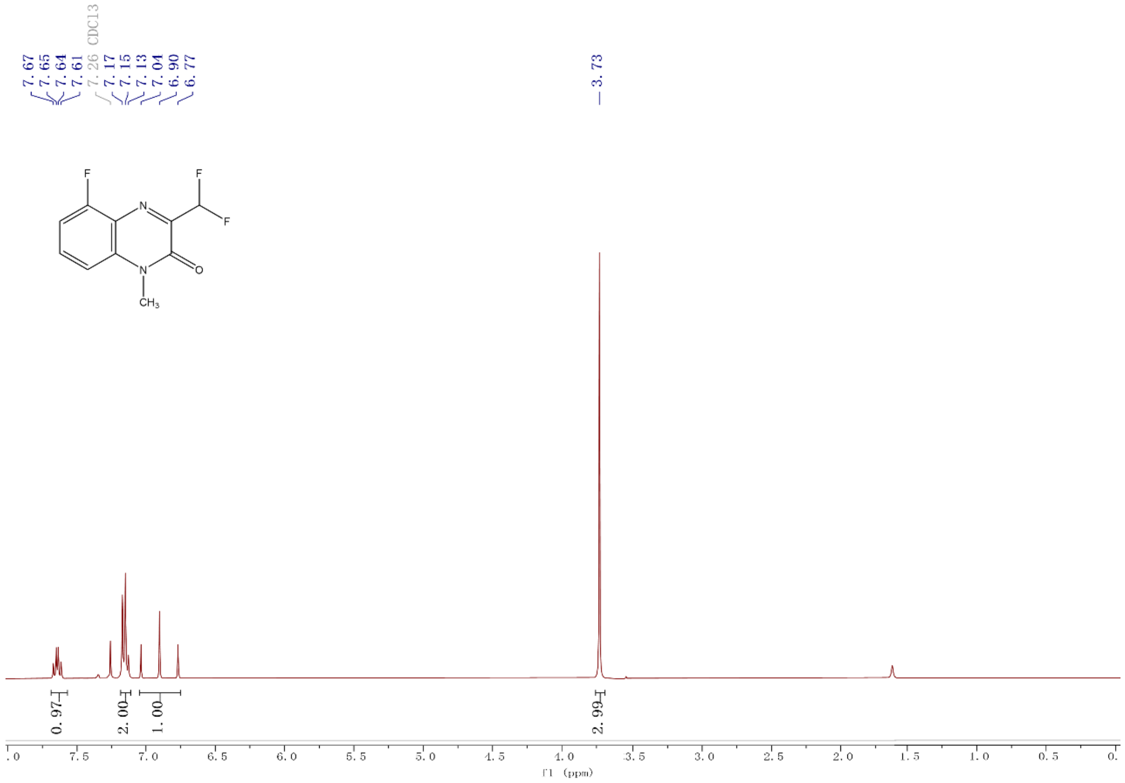

The ^{1}H-NMR Spectrum of **1e**.

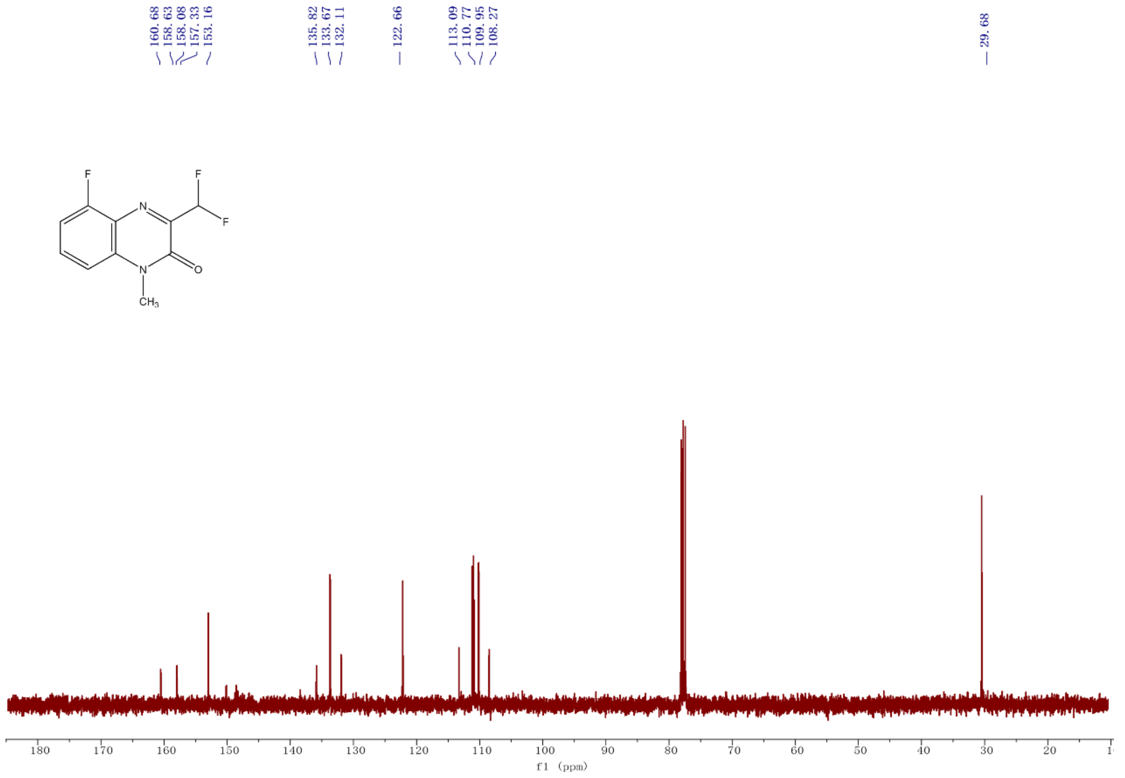

The ^{13}C-NMR Spectrum of **1e**.

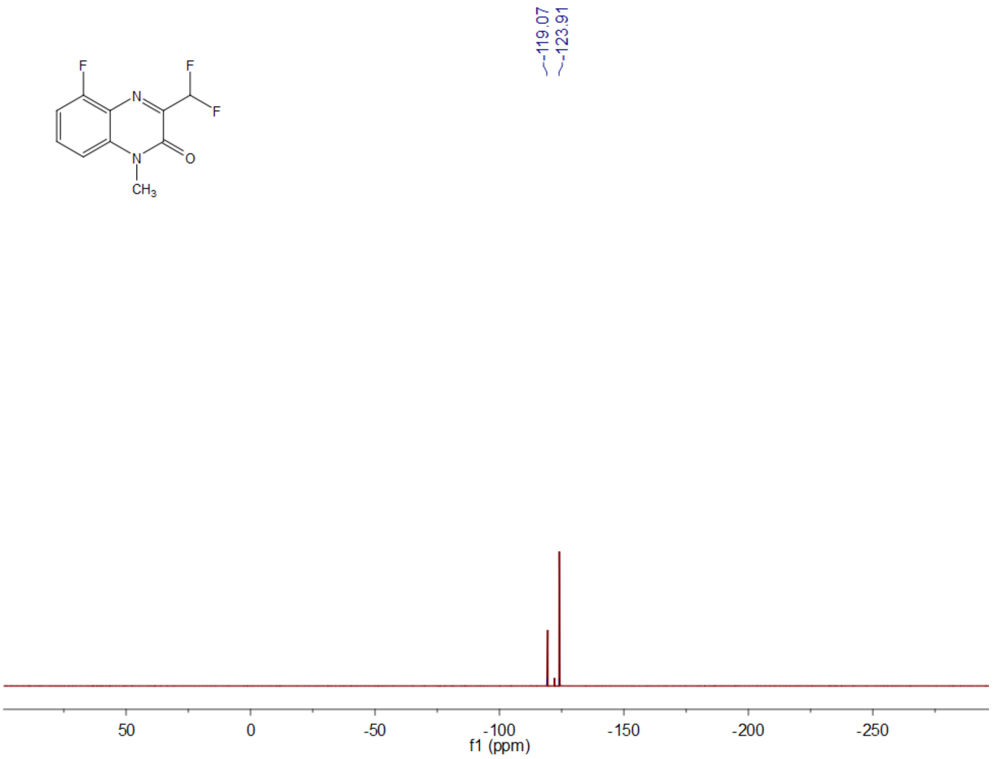

The ^{19}F-NMR Spectrum of **1e**.

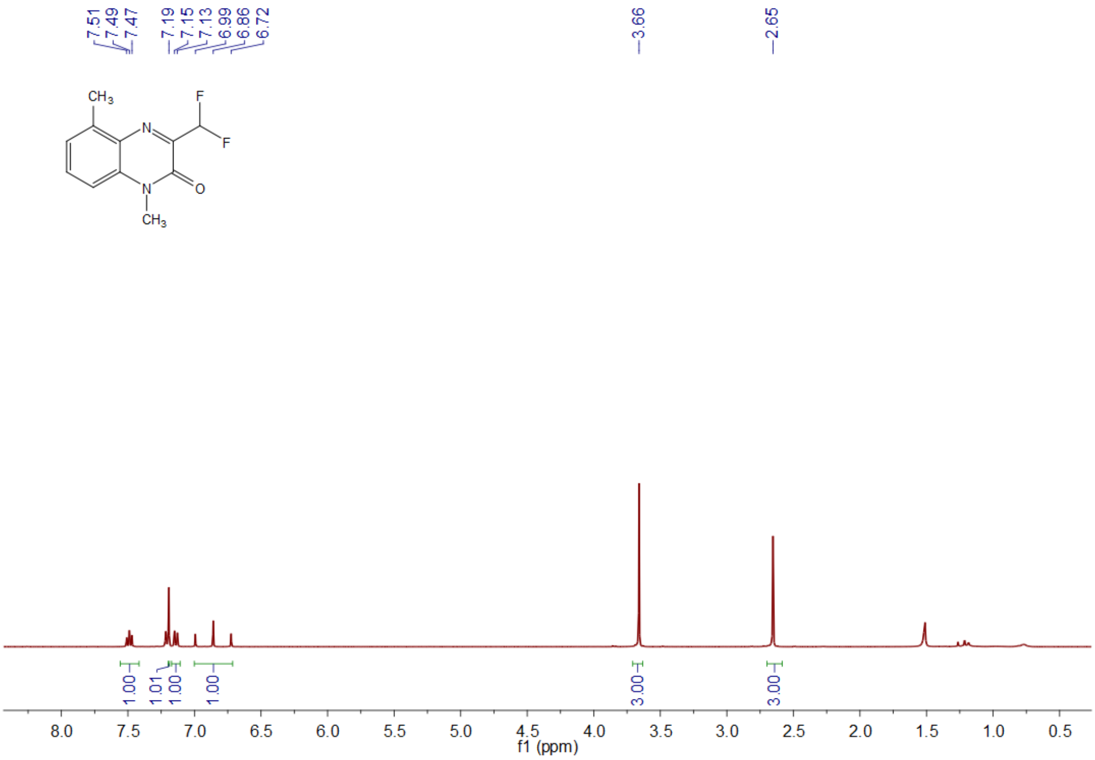

The ^{1}H-NMR Spectrum of **1f**.

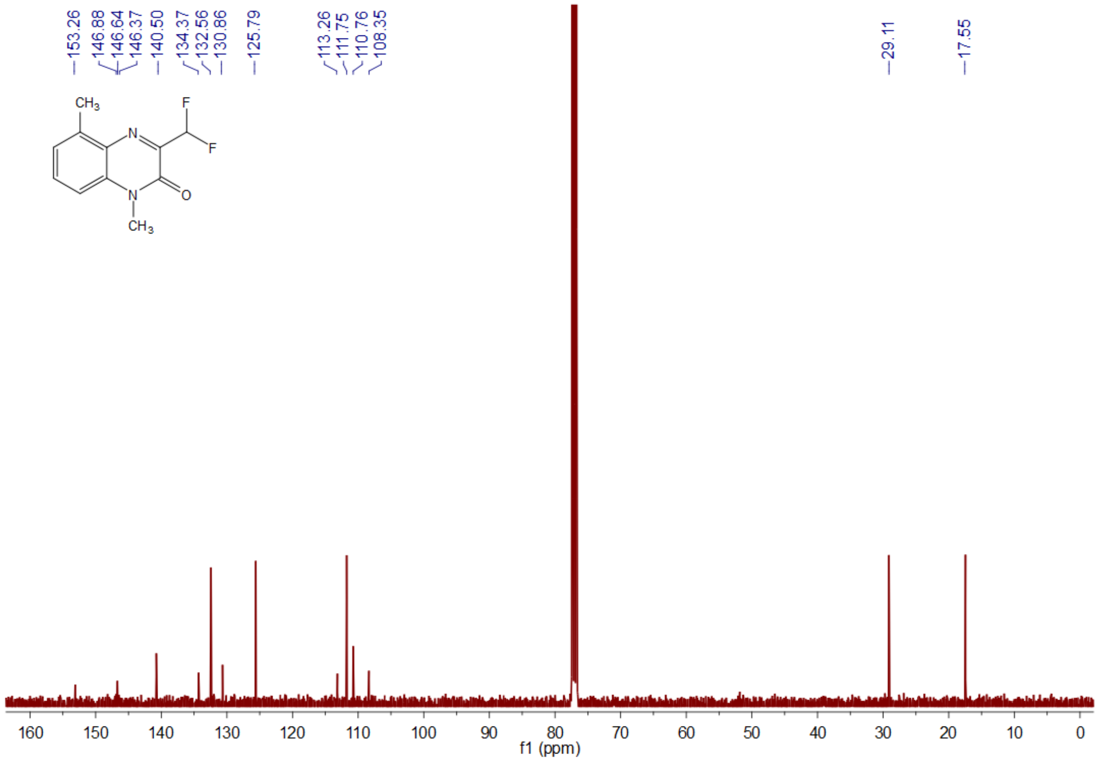

The ^{13}C-NMR Spectrum of **1f**.

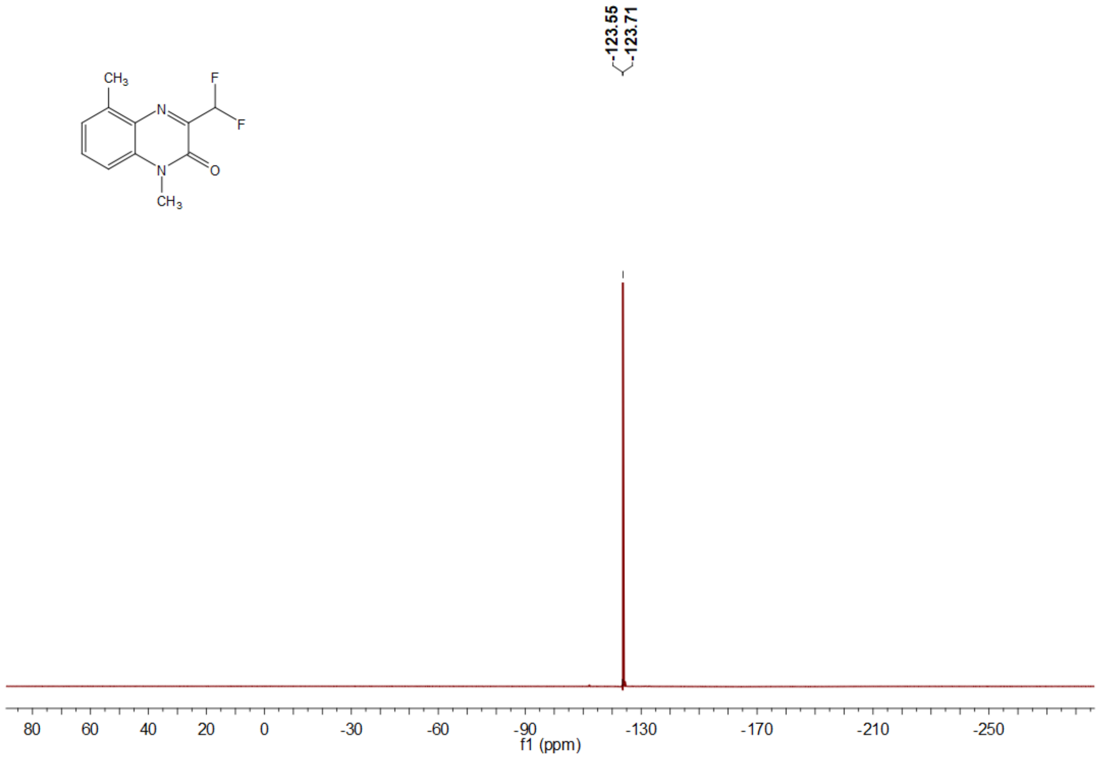

The ^{19}F-NMR Spectrum of **1f**.

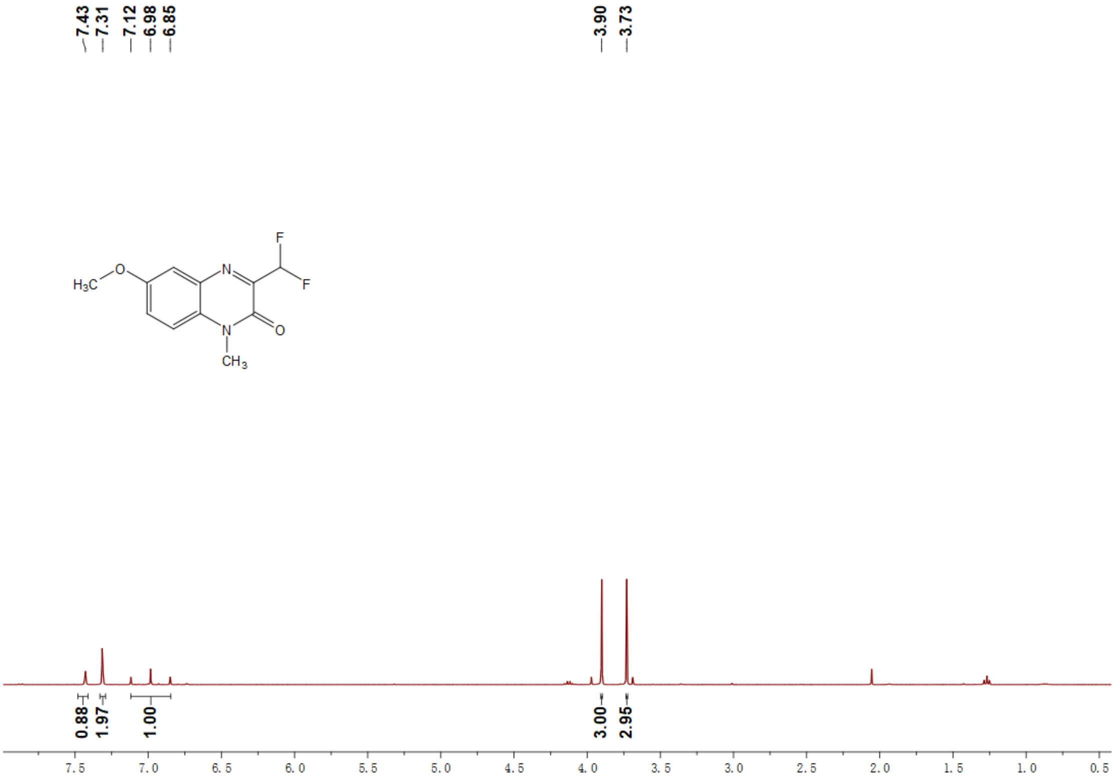

The ^{1}H-NMR Spectrum of **1g**.

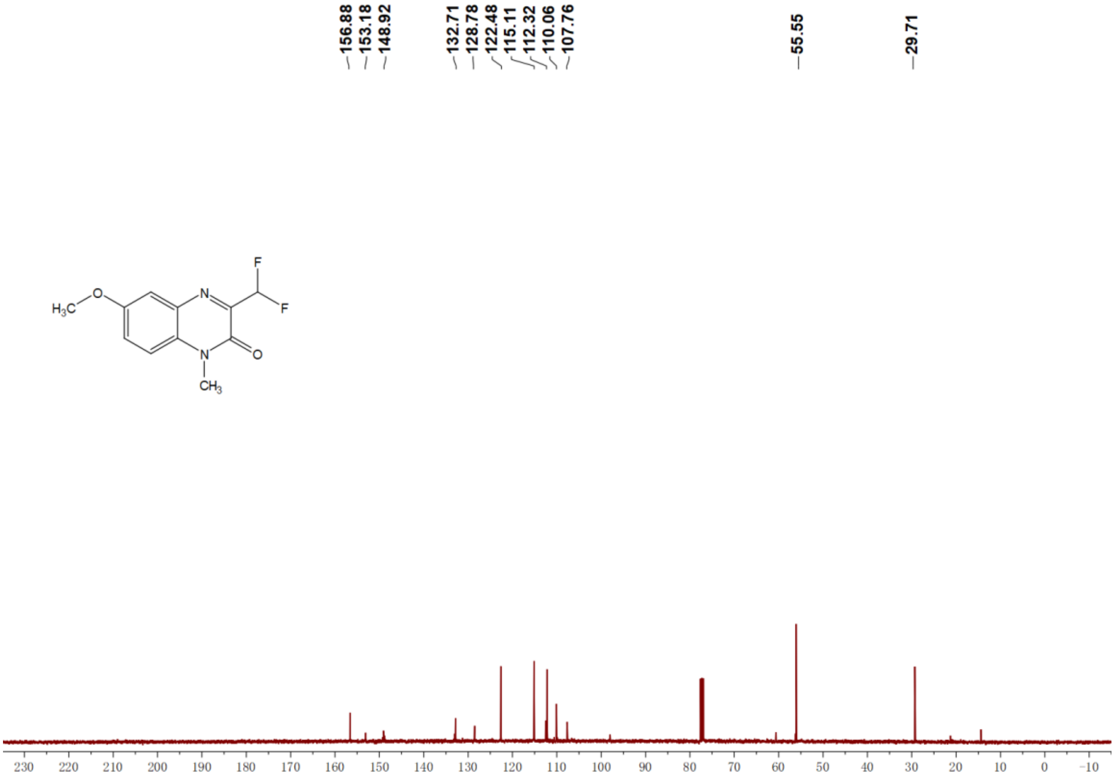

The ^{13}C-NMR Spectrum of **1g**.

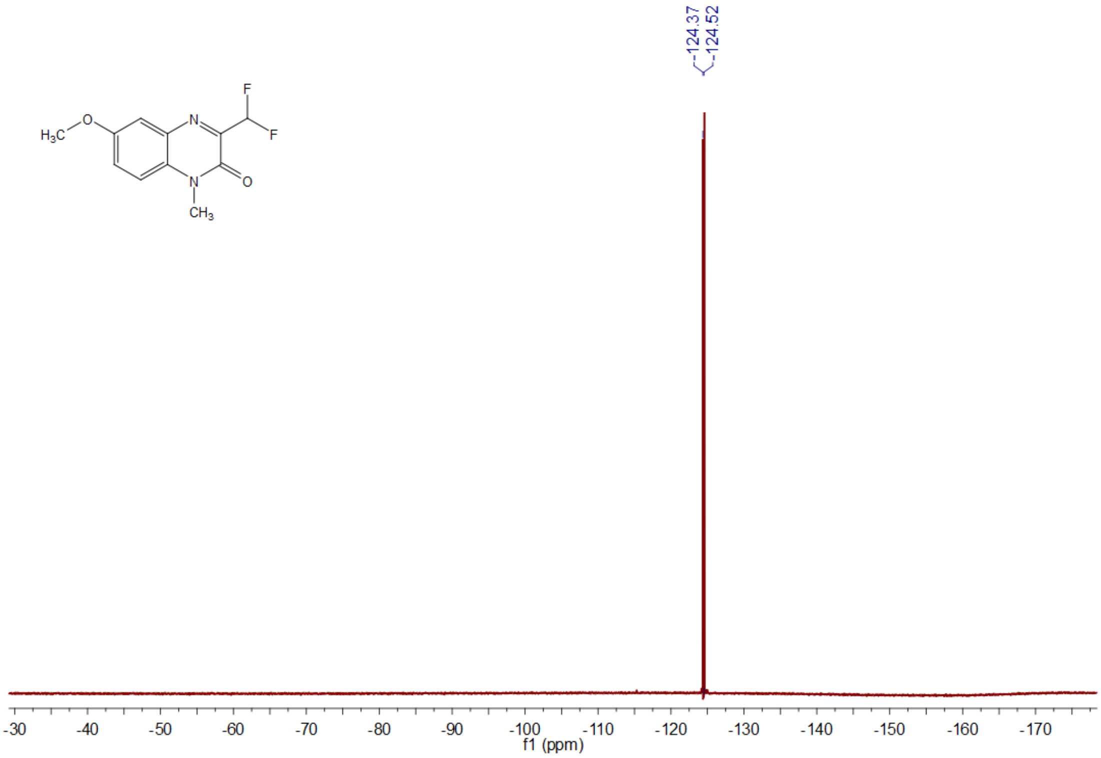

The ^{19}F-NMR Spectrum of **1g**.

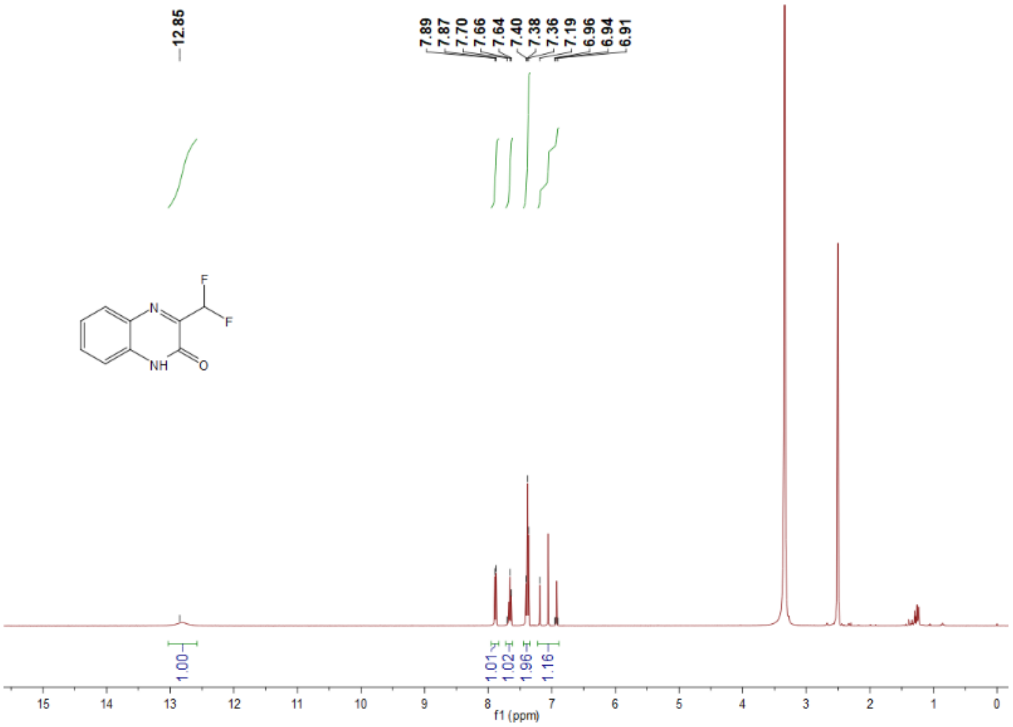

The ^{1}H-NMR Spectrum of **1h**.

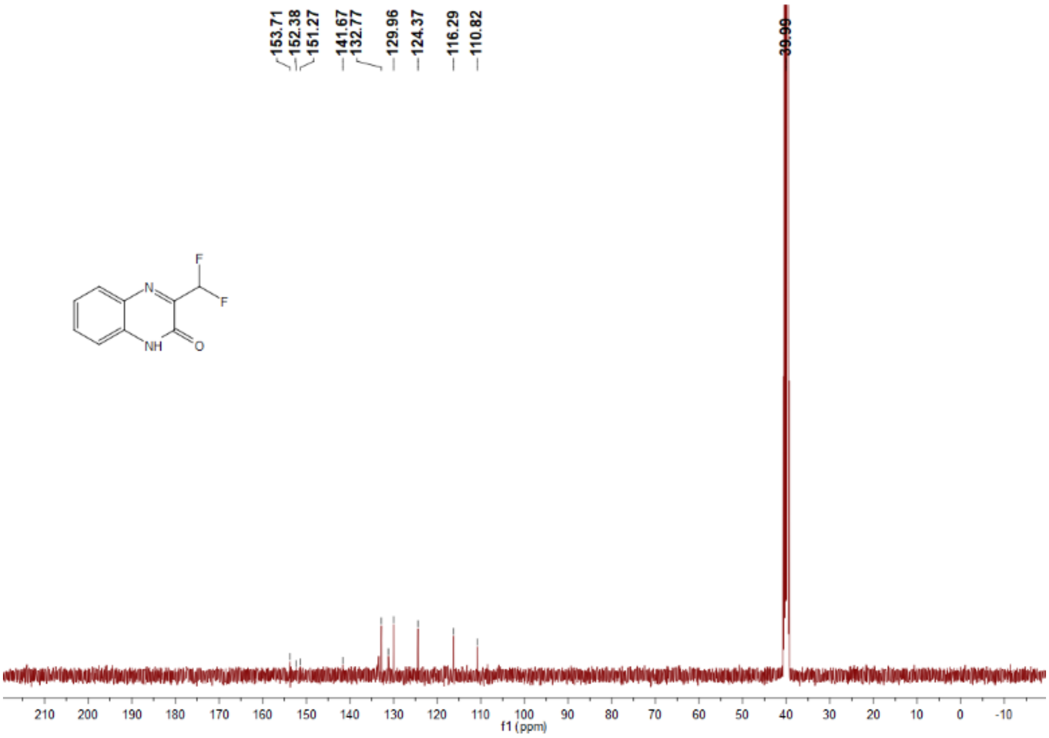

The ^{13}C-NMR Spectrum of **1h**.

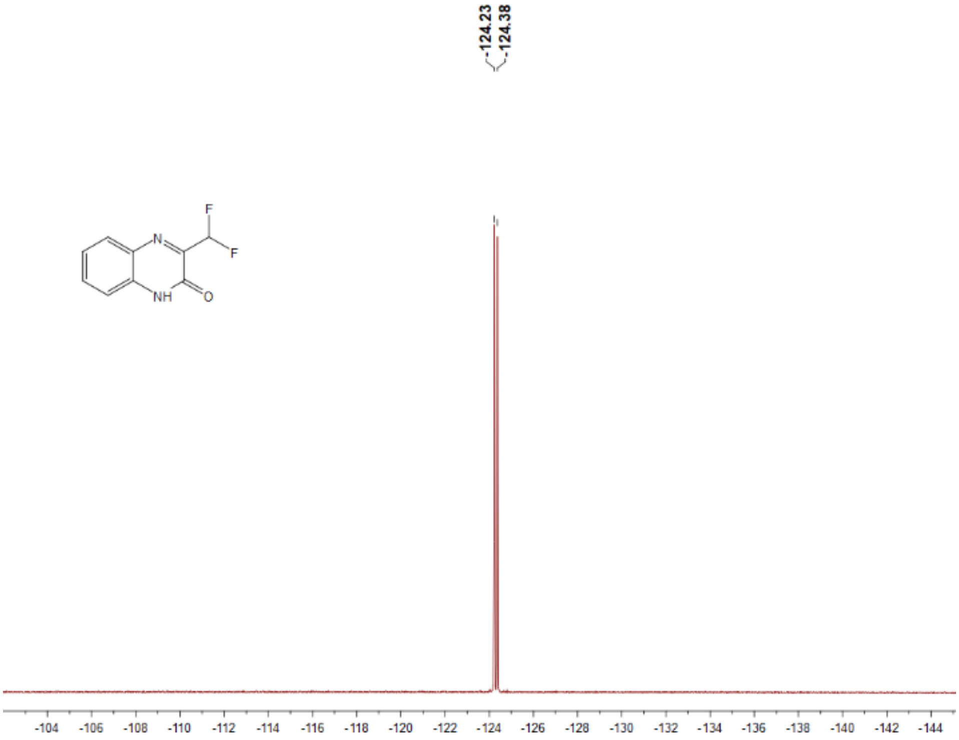

The ^{19}F-NMR Spectrum of **1h**.

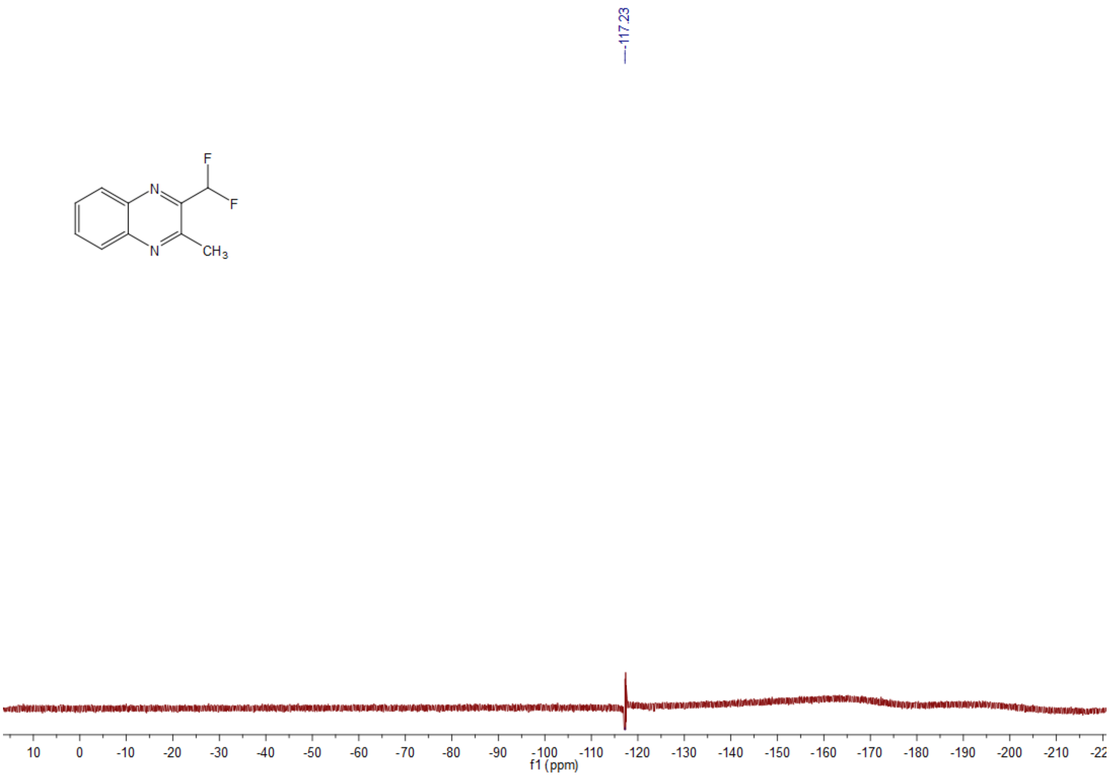

The ^{19}F-NMR Spectrum of **1i**.

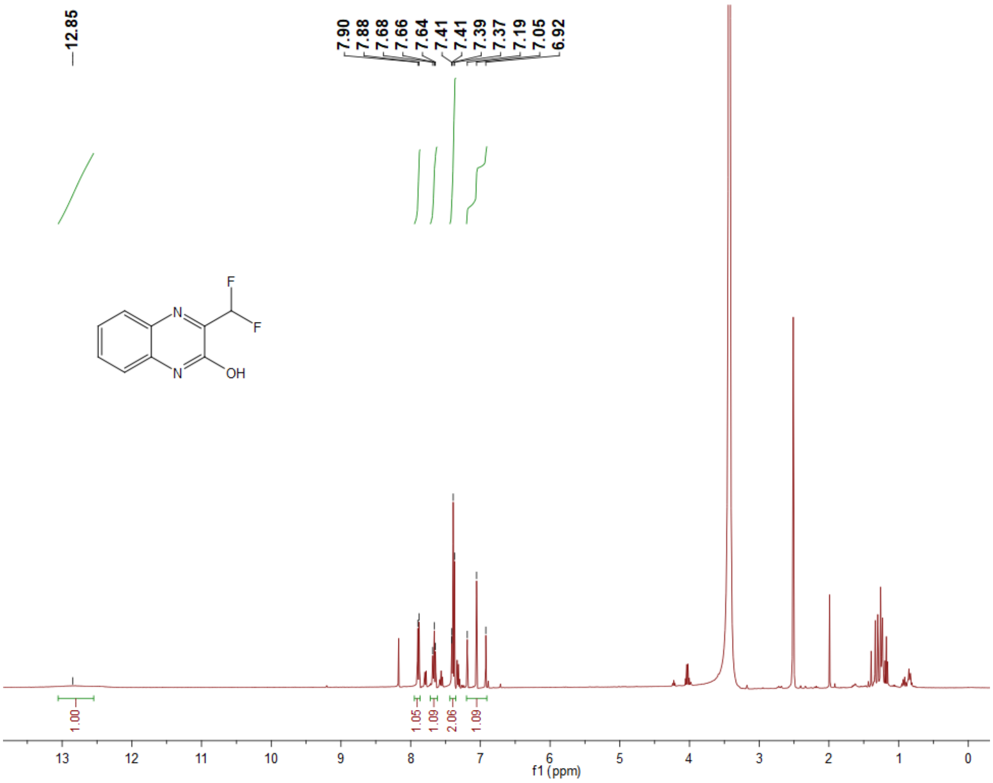

The ^{1}H-NMR Spectrum of **1j**.

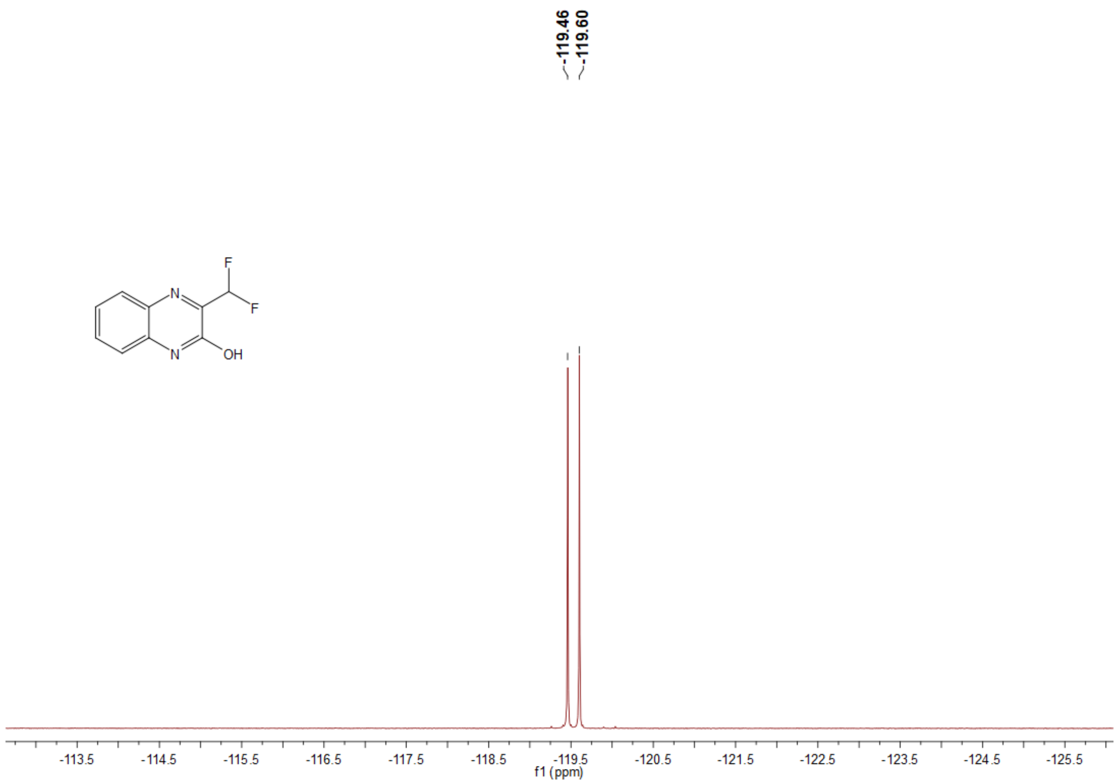

The ^{19}F-NMR Spectrum of **1j**.

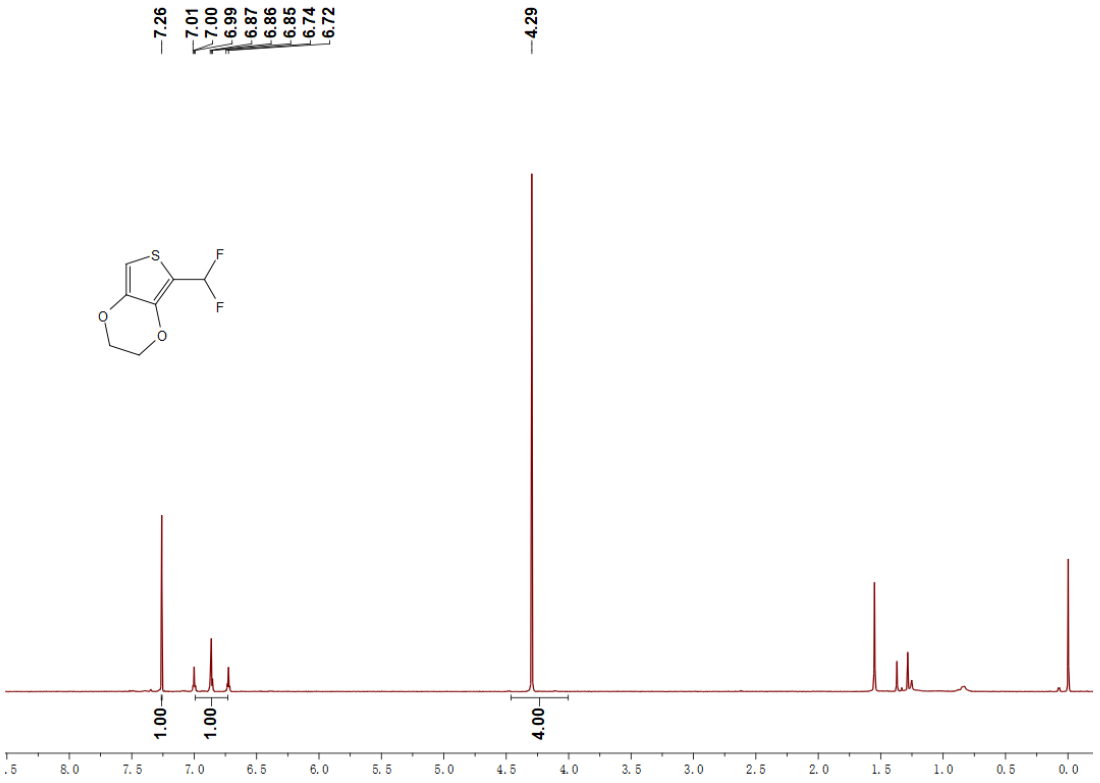

The ^{1}H-NMR Spectrum of **1k**.

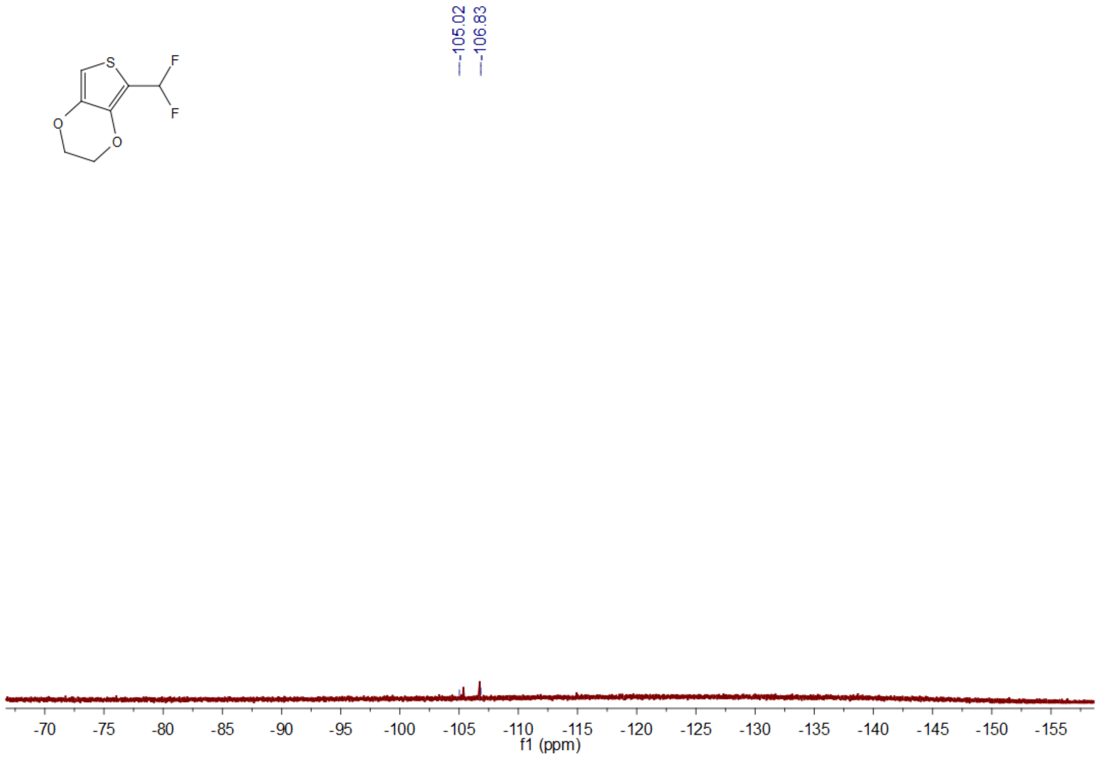

The ^{19}F-NMR Spectrum of **1k**.

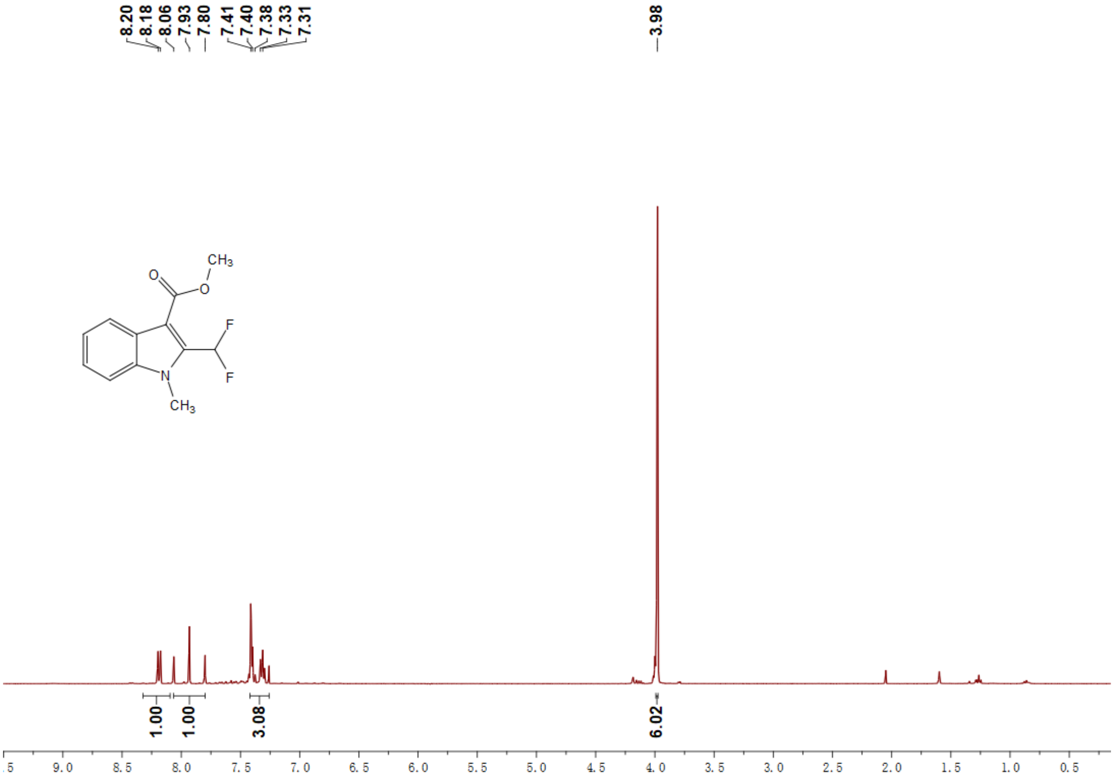

The ^{1}H-NMR Spectrum of **1l**.

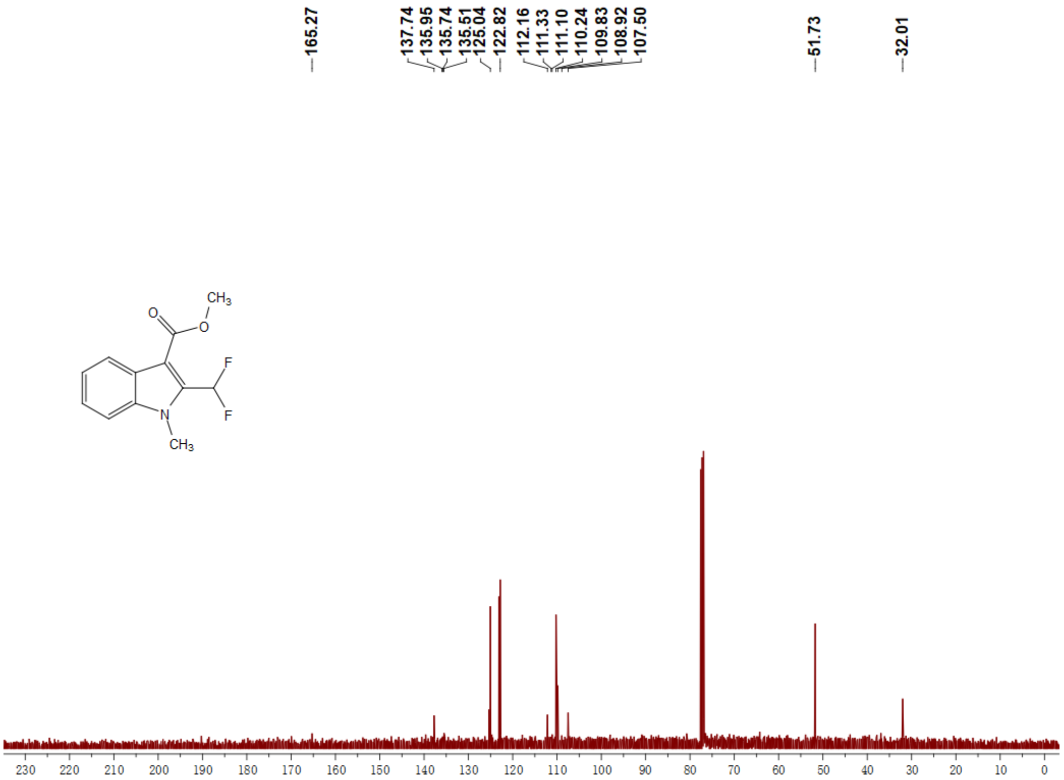

The ^{13}C-NMR Spectrum of **1l**.

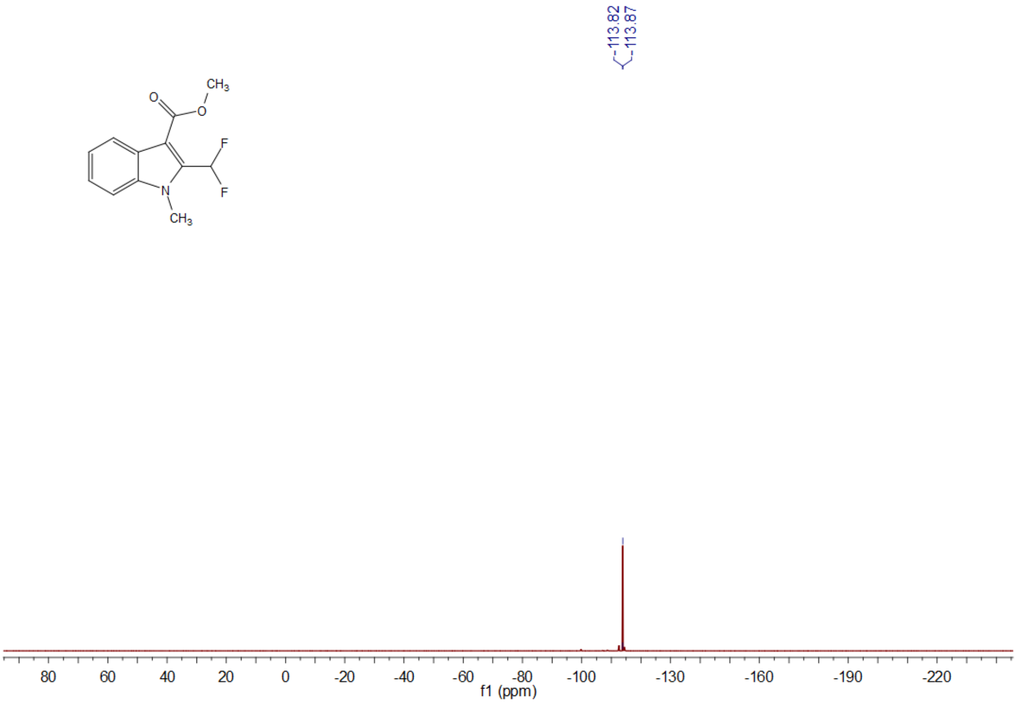

The ^{19}F-NMR Spectrum of **1l**.

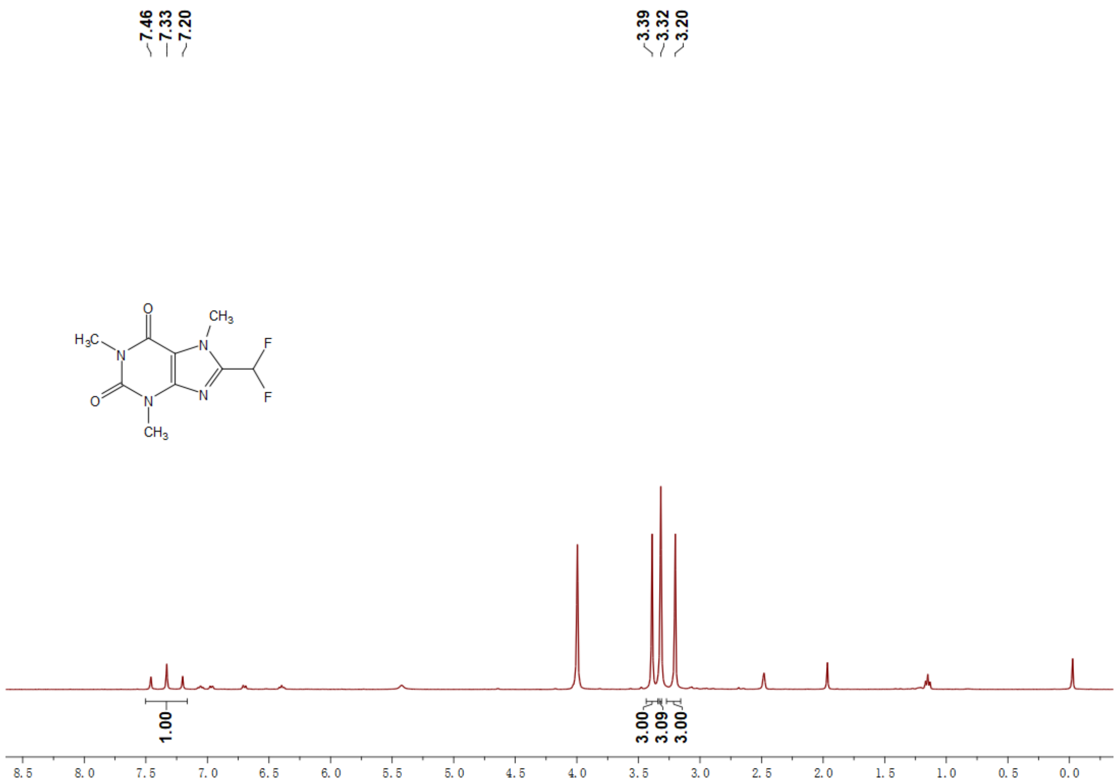

The ^{1}H-NMR Spectrum of **1m**.

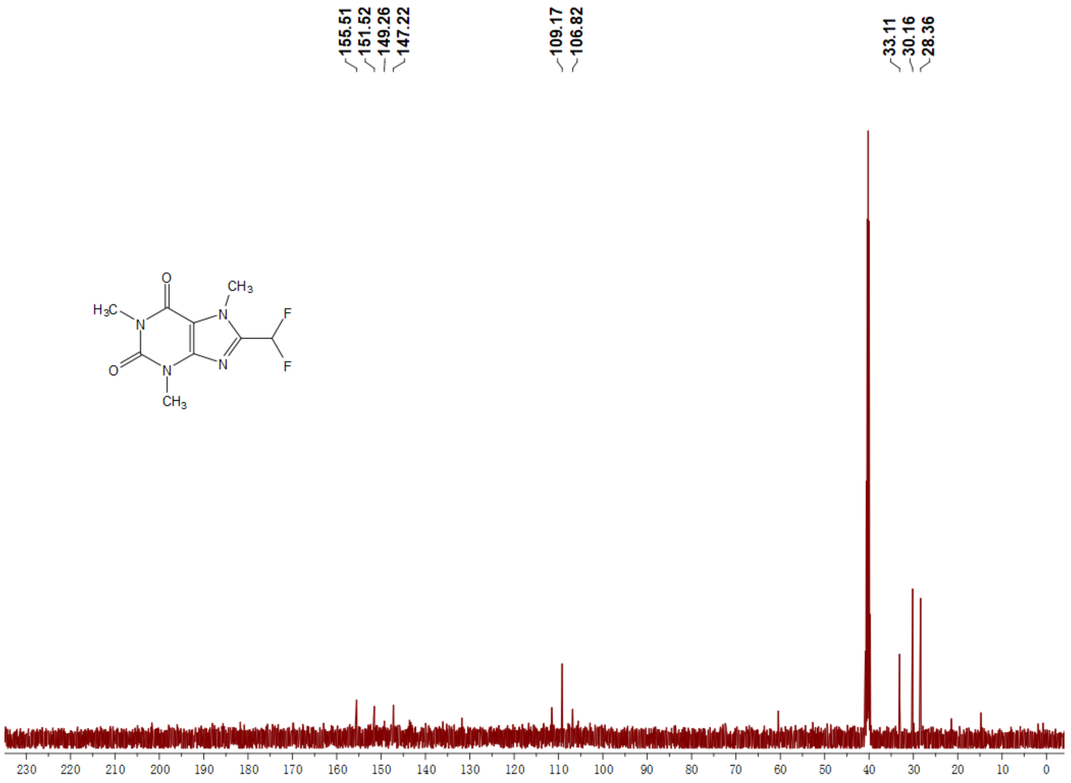

The ^{13}C-NMR Spectrum of **1m**.

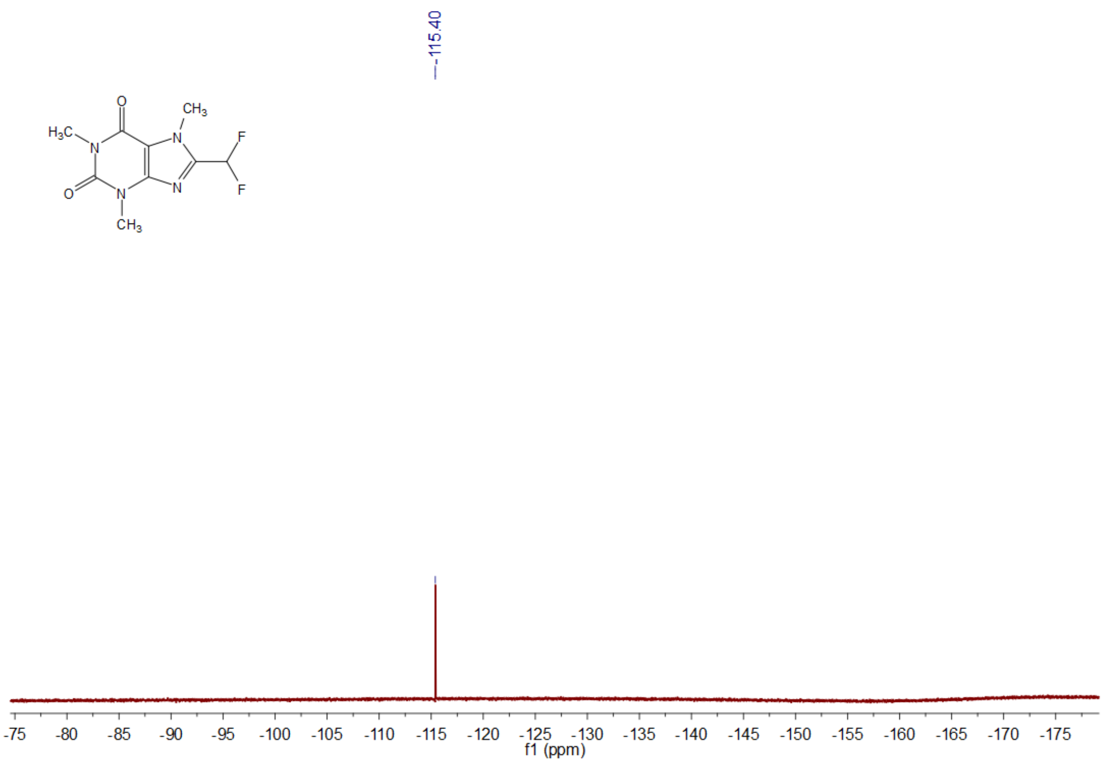

The ^{19}F-NMR Spectrum of **1m**.

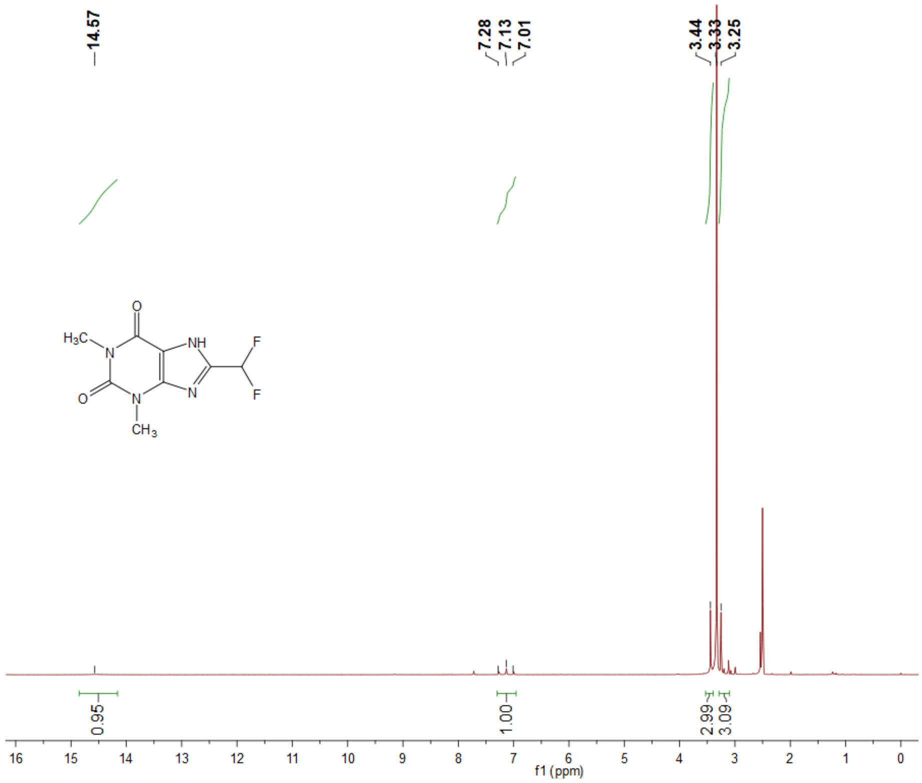

The ^{1}H-NMR Spectrum of **1n**.

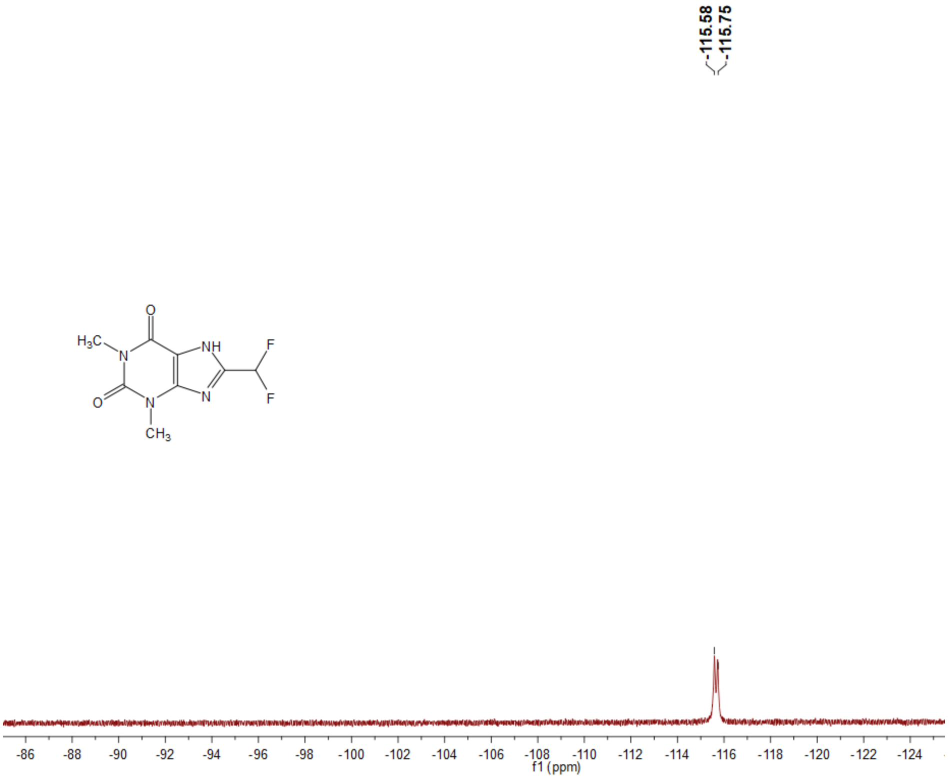

The ^{19}F-NMR Spectrum of **1n**.

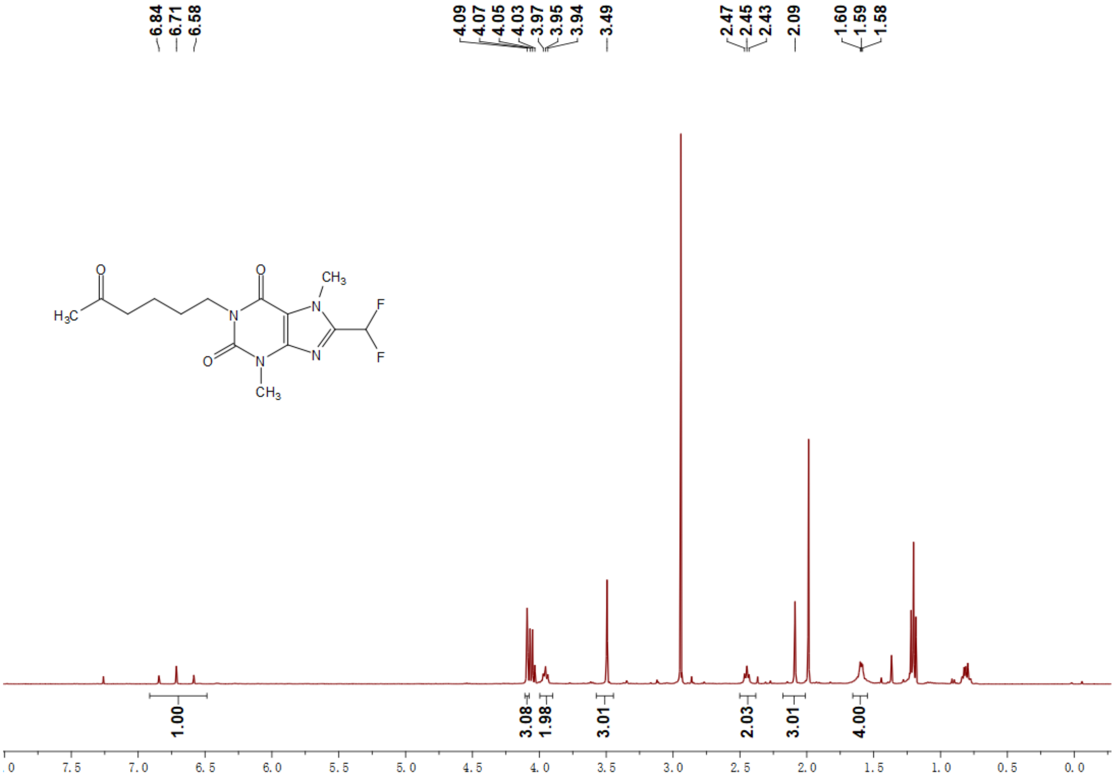

The ^{1}H-NMR Spectrum of **1o**.

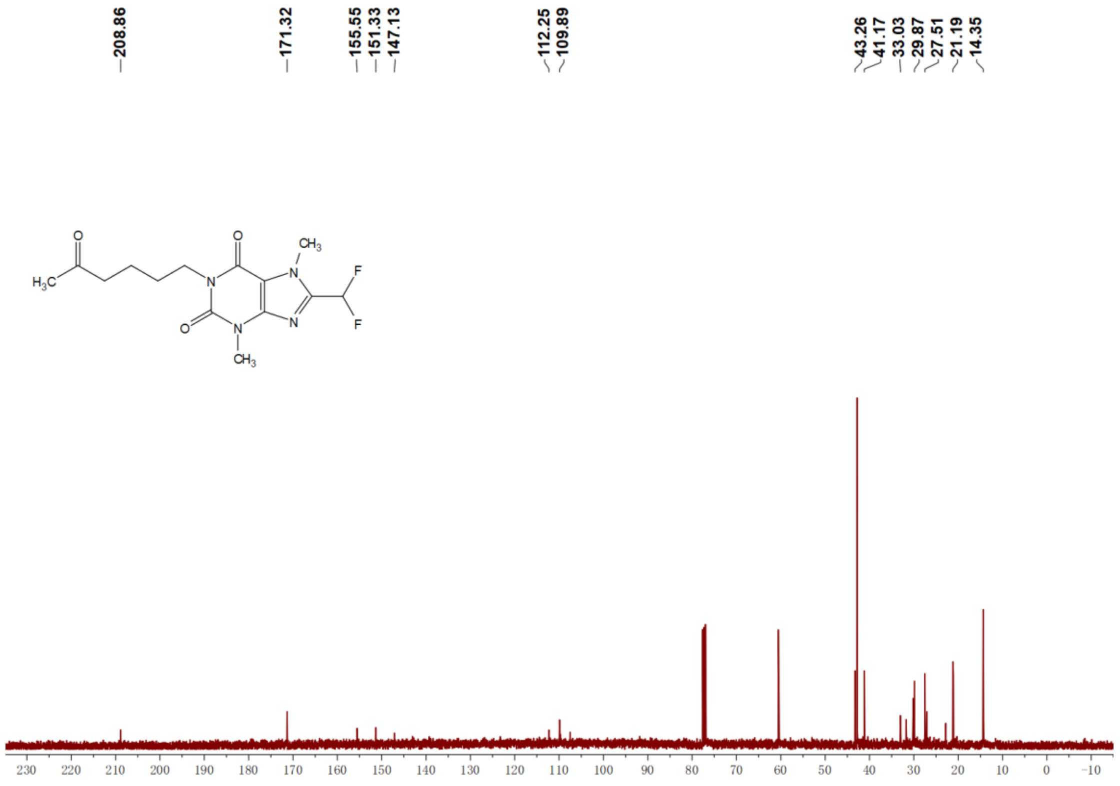

The ^{13}C-NMR Spectrum of **1o**.

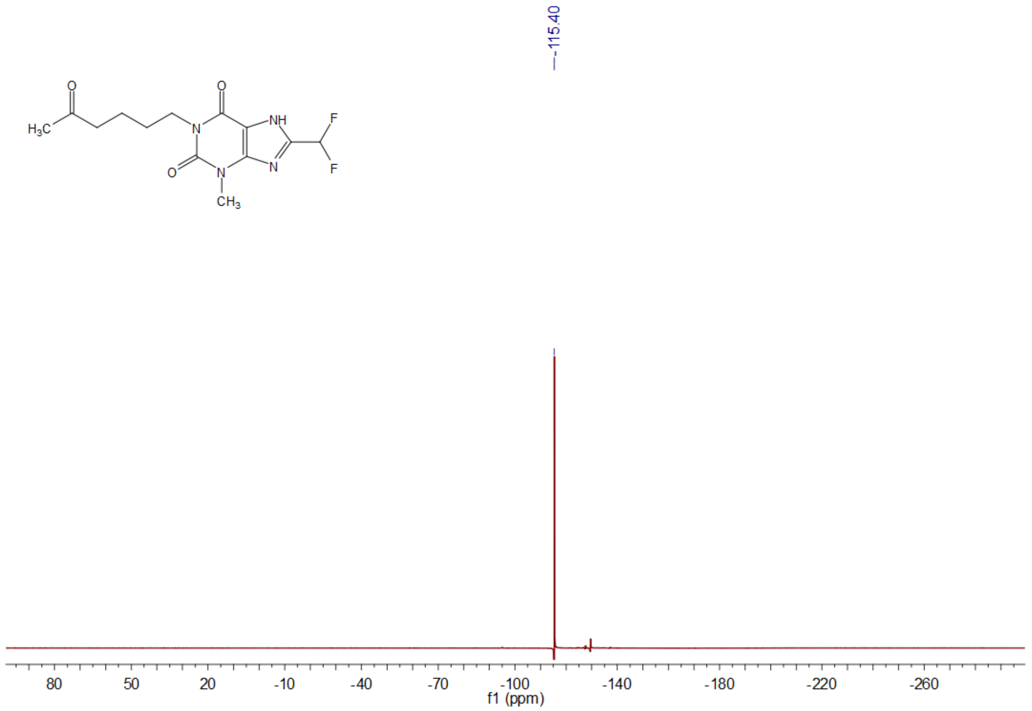

The ^{19}F-NMR Spectrum of **1o**.

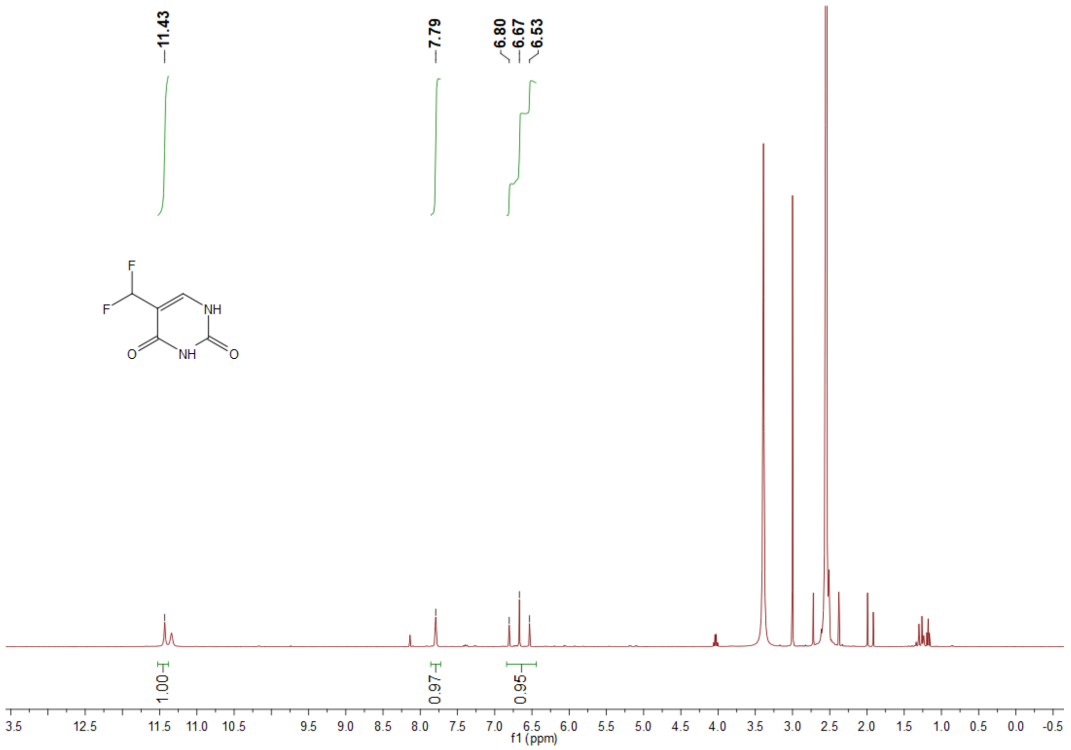

The ^{1}H-NMR Spectrum of **1p**.

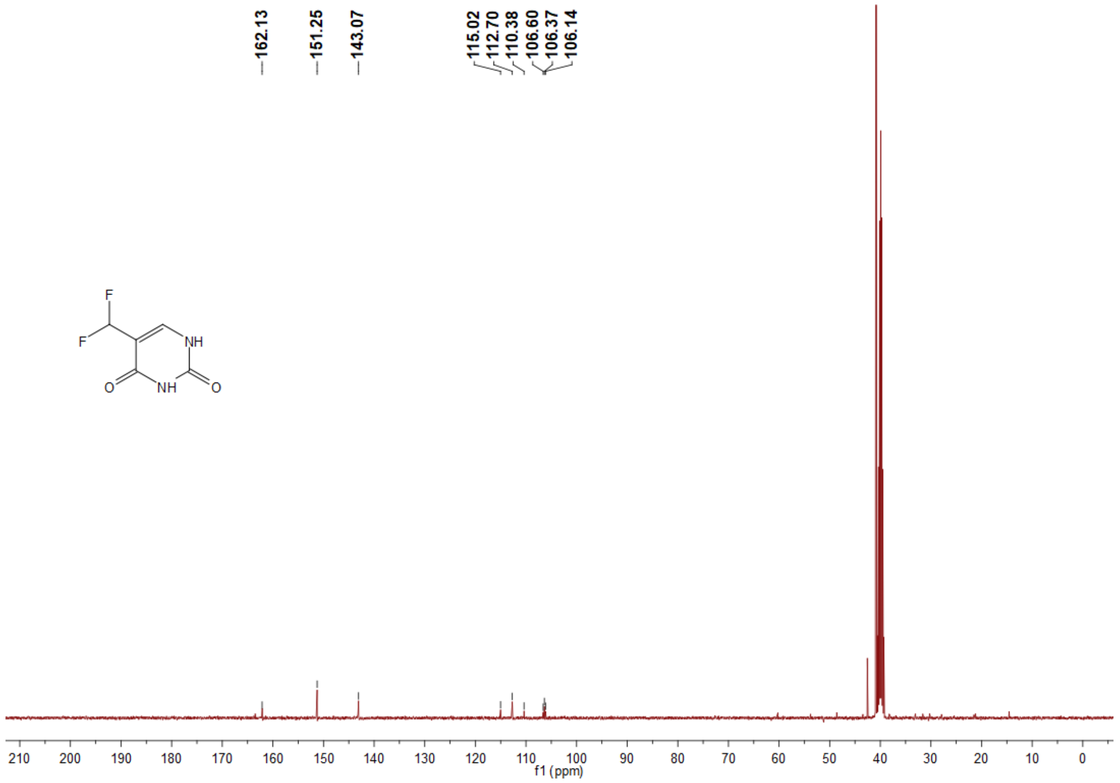

The ^{13}C-NMR Spectrum of **1p**.

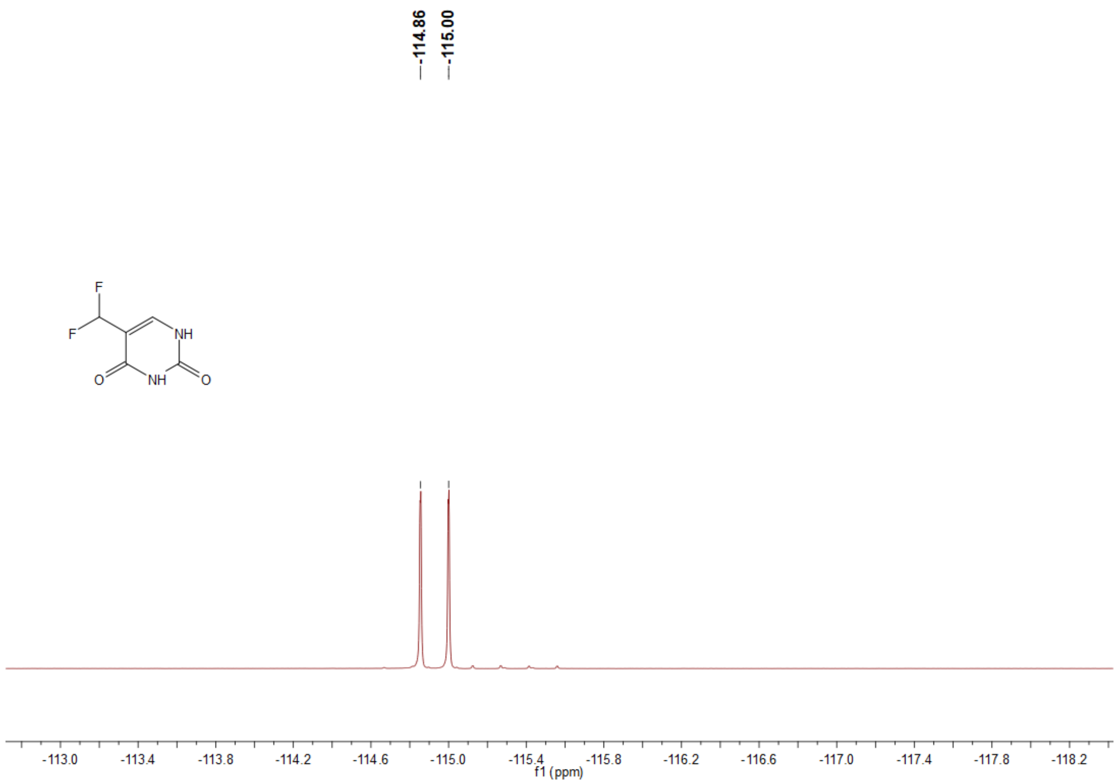

The ^{19}F-NMR Spectrum of **1p**.

REFERENCE

(1) Wei, S.; Zhang, F.; Zhang, W.; Qiang, P.; Yu, K.; Fu, X.; Wu, D.; Bi, S.; Zhang, F. Semiconducting 2D Triazine-Cored Covalent Organic Frameworks with Unsubstituted Olefin Linkages. *J. Am. Chem. Soc.* **2019**, *141* (36), 14272-14279. DOI: 10.1021/jacs.9b06219.

(2) Acharjya, A.; Pachfule, P.; Roeser, J.; Schmitt, F.-J.; Thomas, A. Vinylene-Linked Covalent Organic Frameworks by Base-Catalyzed Aldol Condensation. *Angew. Chem. Int. Ed.* **2019**, *58* (42), 14865-14870. DOI: 10.1002/anie.201905886.

(3) Zhang, W.; Xiang, X.-X.; Chen, J.; Yang, C.; Pan, Y.-L.; Cheng, J.-P.; Meng, Q.; Li, X. Direct C–H difluoromethylation of heterocycles via organic photoredox catalysis. *Nat. Commun.* **2020**, *11* (1), 638. DOI: 10.1038/s41467-020-14494-8.

(4) Jadhav, T.; Fang, Y.; Liu, C.-H.; Dadvand, A.; Hamzehpoor, E.; Patterson, W.; Jonderian, A.; Stein, R. S.; Perepichka, D. F. Transformation between 2D and 3D Covalent Organic Frameworks via Reversible [2 + 2] Cycloaddition. *J. Am. Chem. Soc.* **2020**, *142* (19), 8862-8870. DOI: 10.1021/jacs.0c01990.

(5) Dai, P.; Yu, X.; Teng, P.; Zhang, W.-H.; Deng, C. Visible-Light- and Oxygen-Promoted Direct Csp2-H Radical Difluoromethylation of Coumarins and Antifungal Activities. *Org. Lett.* **2018**, *20* (21), 6901-6905. DOI: 10.1021/acs.orglett.8b02965.

(6) Arroyo, A. D.; Guzmán, A. E.; Kachur, A. V.; Saylor, S. J.; Popov, A. V.; Delikatny, E. J. Development of fluorinated naphthofluoresceins for Cerenkov imaging. *Journal of Fluorine Chemistry* **2019**, *225*, 27-34. DOI: 10.1016/j.jfluchem.2019.05.010.

(7) Mao, S.; Wang, H.; Liu, L.; Wang, X.; Zhou, M.-D.; Li, L. Trifluoromethylation/Difluoromethylation-Initiated Radical Cyclization of o-Alkenyl Aromatic Isocyanides for Direct Construction of 4-Cyano-2-Trifluoromethyl/Difluoromethyl-Containing Quinolines. *Adv. Synth. Catal.* **2020**, *362* (11), 2274-2279. DOI: 10.1002/adsc.202000155.

(8) Ghosh, I.; Khamrai, J.; Savateev, A.; Shlapakov, N.; Antonietti, M.; König, B. Organic semiconductor photocatalyst can bifunctionalize arenes and heteroarenes. *Science* **2019**, *365* (6451), 360-366. DOI: 10.1126/science.aaw3254.

(9) Yuan, X.; Duan, X.; Cui, Y.-S.; Sun, Q.; Qin, L.-Z.; Zhang, X.-P.; Liu, J.; Wu, M.-Y.; Qiu, J.-K.; Guo, K. Visible-Light Photocatalytic Tri- and Difluoroalkylation Cyclizations: Access to a Series of Indole[2,1-a]isoquinoline Derivatives in Continuous Flow. *Org. Lett.* **2021**, *23* (5), 1950-1954. DOI: 10.1021/acs.orglett.1c00476.

(10) Xiong, W.; Qin, W.-B.; Zhao, Y.-S.; Fu, K.-Z.; Liu, G.-K. Direct C(sp3)–H difluoromethylation via radical–radical cross-coupling by visible-light photoredox catalysis. *Org. Chem. Front.* **2022**, *9* (8), 2141-2148, 10.1039/D2QO00192F. DOI: 10.1039/D2QO00192F.

(11) Vytla, D.; Kaliyaperumal, K.; Velayuthaperumal, R.; Shaw, P.; Gautam, R.; Mathur, A.; Roy, A. Visible-Light-Promoted Radical Cyclization of N-Arylvinylsulfonamides: Synthesis of CF3/CHF2/CH2CF3-Containing 1,3-Dihydrobenzo[c]isothiazole 2,2-Dioxide Derivatives. *Synthesis* **2021**, *54* (03), 667-682. DOI: 10.1055/s-0040-1720921.

(12) Rong, J.; Deng, L.; Tan, P.; Ni, C.; Gu, Y.; Hu, J. Radical Fluoroalkylation of Isocyanides with Fluorinated Sulfones by Visible-Light Photoredox Catalysis. *Angew. Chem. Int. Ed.* **2016**, *55* (8), 2743-2747. DOI: 10.1002/anie.201510533.
